# Al(III)/K(I) Heterodinuclear Polymerization Catalysts Showing Fast Rates and High Selectivity for Polyester Polyols

**DOI:** 10.1021/acscatal.3c05712

**Published:** 2024-01-11

**Authors:** Edward J. K. Shellard, Wilfred T. Diment, Diego A. Resendiz-Lara, Francesca Fiorentini, Georgina L. Gregory, Charlotte K. Williams

**Affiliations:** Chemistry Research Laboratory, Department of Chemistry, University of Oxford, 12 Mansfield Road, Oxford OX1 3TA, U.K.

**Keywords:** catalysts, monomers, aluminum, potassium, polyester, ring-opening copolymerization, chain transfer agent, synergy

## Abstract

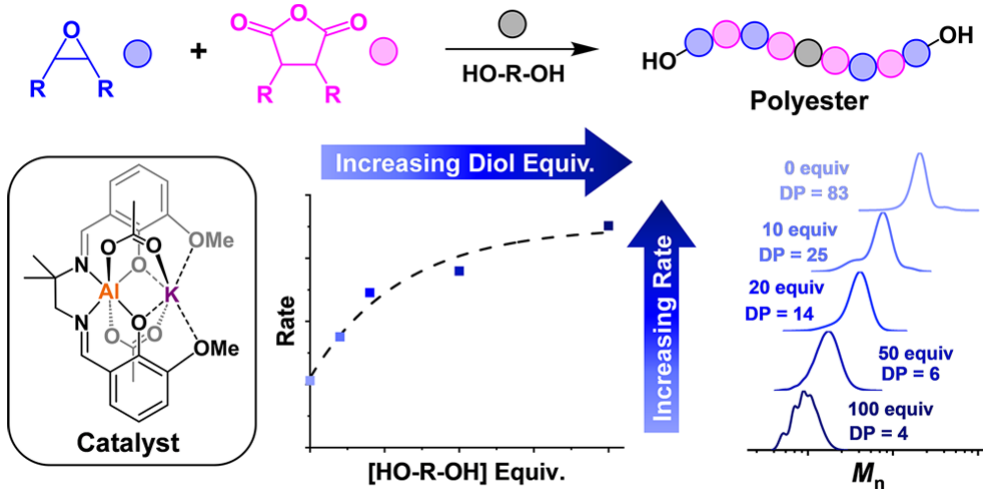

Low molar mass, hydroxyl end-capped polymers, often termed “polyols,” are widely used to make polyurethanes, resins, and coatings and as surfactants in liquid formulations. Epoxide/anhydride ring-opening copolymerization (ROCOP) is a controlled polymerization route to make them, and its viability depends upon catalyst selection. In the catalysis, the polyester polyol molar masses and end-groups are controlled by adding specific but excess quantities of diols (vs catalyst), known as the chain transfer agent (CTA), to the polymerizations, but many of the best current catalysts are inhibited or even deactivated by alcohols. Herein, a series of air-stable Al(III)/K(I) heterodinuclear polymerization catalysts show rates and selectivity at the upper end of the field. They also show remarkable increases in activity, with good selectivity and control, as quantities of diol are increased from 10–400 equiv. The reactions are accelerated by alcohols, and simultaneously, their use allows for the production of hydroxy telechelic poly/oligoesters (400 < *M_n_* (g mol^–1^) < 20,400, *Đ* < 1.19). For example, cyclohexene oxide (CHO)/phthalic anhydride (PA) ROCOP, using the best Al(III)/K(I) catalyst with 200 equiv of diol, shows a turnover frequency (TOF) of 1890 h^–1^, which is 4.4× higher than equivalent reactions without any diol (Catalyst/Diol/PA/CHO = 1:10–400:400:2000, 100 °C). In all cases, the catalysis is well controlled and highly ester linkage selective (ester linkages >99%) and operates effectively using bicyclic and/or biobased anhydrides with bicyclic or flexible alkylene epoxides. These catalysts are recommended for future production and application development using polyester polyols.

## Introduction

Consumer and environmental pressure on the polymer industry to improve sustainability and limit pollution requires a major shift in materials manufacturing, use, and disposal.^1−7^ One obvious challenge is that the term “plastics” covers a multitude of polymer chemistries, application sectors, lifetimes, and disposal options, with varying extents of material recoverability.^1−7^ This work focuses on polymers which are generally “irretrievable” after use, since they are used in formulated products, including for household cleaning, personal care, beauty, medicine, agricultural coatings, and paints, as thickeners, emulsifiers, or stabilizers.^8,9^ One future vision to improve the sustainability of these products is to prepare them from waste biomass and design them to fully degrade to nontoxic metabolites after use.^7,9−11^ Delivery of such products requires improvements to the production of degradable polymers; this work focuses on polyesters.^6,7^ Polyesters are selected because many of the monomers can be derived from renewable feedstocks, and a range of enzymes, both natural and modified, continue to be discovered for ester hydrolysis and, in some cases, for biodegradation.^2,6,7^ Furthermore, the byproducts of such degradations—diols and diacids—are often metabolites and/or show low toxicity.^6^

Current large-scale polyester production is usually accomplished by step-growth polymerizations achieved with carefully managed high-temperature conditions.^12^ Such methods are difficult or impossible to use to prepare well-defined polyesters, i.e., those with targeted degrees of polymerization (DP) and/or narrow molar mass distributions. Well-defined polyesters are useful to inform structure–property relationships and are essential to make more complex materials, including block polymers.^13−15^ Cyclic ester ring-opening polymerization (ROP) provides a well-controlled route to selected polyesters, most usually with aliphatic backbone chemistries, but is somewhat restricted by monomer availability and ring-strain (thermodynamics); it is generally less well suited to making functionalized polyesters.^12,13^ The ring-opening copolymerization (ROCOP) of epoxides and anhydrides could be a very useful controlled polymerization since it shows broader monomer scope, with many epoxides/anhydrides already in commercial use in polymer manufacturing, and it efficiently delivers aliphatic, semiaromatic, functionalized, and/or polar polyesters.^12−15^ The best epoxide/anhydride ROCOP catalysts combine high rates and selectivity while operating at low catalyst loadings and showing high levels of polymerization control, but there is still room for improvement in reactions conducted with alcohols.^14−17^ Many successful catalysts are benchmarked using cyclohexene oxide (CHO)/phthalic anhydride (PA) ROCOP.^13−15^ Literature catalysts at the leading end of the field include complexes or salts of Al(III),^17−20^ Cr(III),^21,22^ Y(III),^23,24^ Zn(II),^25,26^ and K(I),^27^ as well as highly active organocatalysts.^28,29^ Many of these successful systems require PPNX cocatalysts that are expensive and may be toxic (where PPN = bis(triphenylphosphine)iminium, X = halide or phenolate, most commonly chloride).^15^ Recently, heterodinuclear catalysts showed very high activities and operated without any cocatalyst.^16^ For example, in 2021, an Al(III)/K(I) complex showed a turnover frequency (TOF) of 1072 h^–1^ (0.25 mol % vs anhydride, 100 °C, CHO/PA).^16,30^ In 2023, an Fe(III)/K(I) catalyst, coordinated by the same ancillary ligand, showed rates up to 3000 h^–1^ (0.025 mol %, 140 °C).^31^

**Figure 1:**
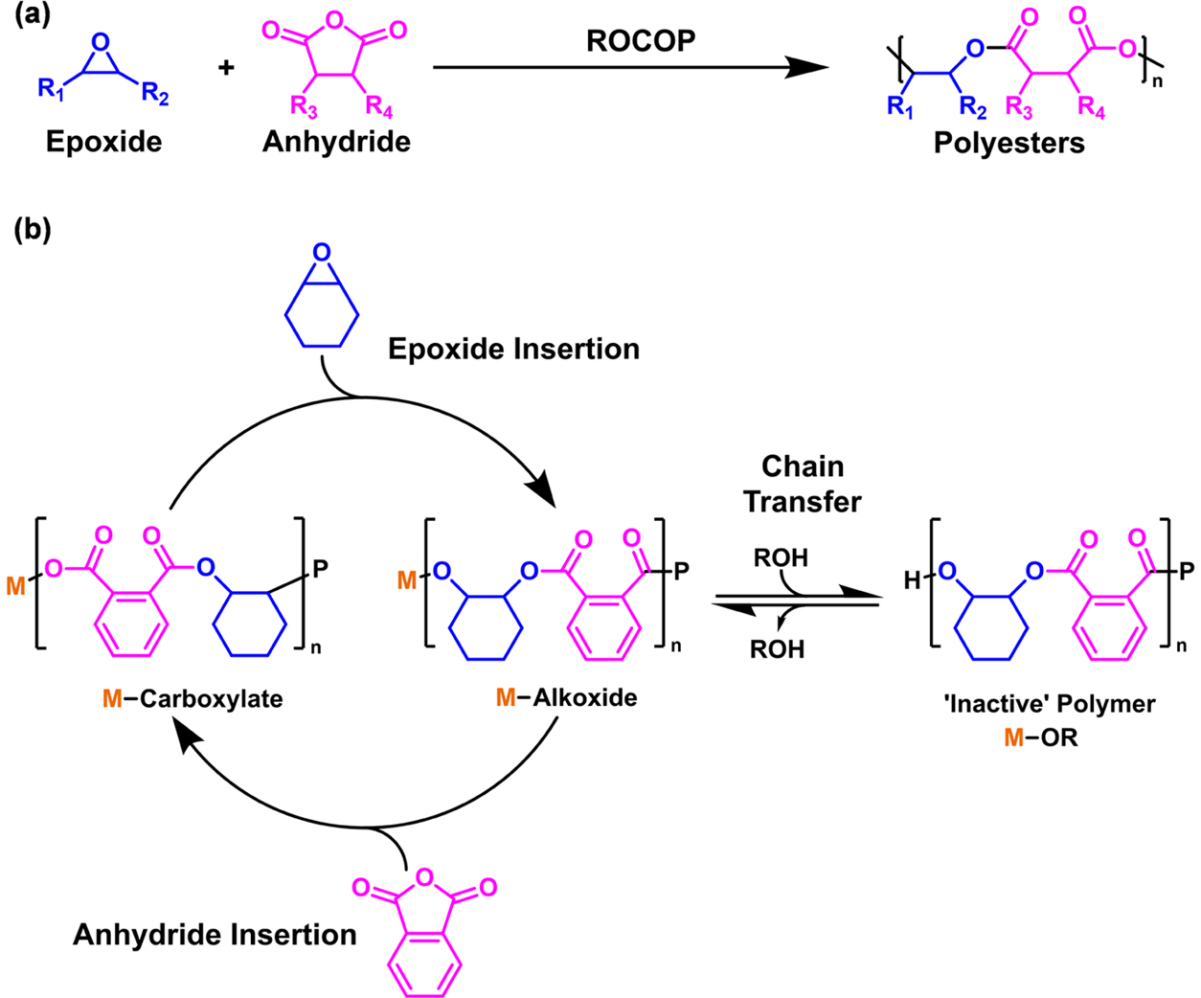
(a) Scheme illustrating epoxide/anhydride ROCOP. (b) Proposed polymerization mechanism illustrating propagation steps for phthalic anhydride and cyclohexene oxide insertions, along with fast and reversible chain transfer reactions with alcohols.

The mechanism for epoxide/anhydride ROCOP catalysis involves the sequential cycling between (metal/ionic) alkoxide (from epoxide insertion) and carboxylate (from anhydride insertion) intermediates (Figure 1).^16^ Using it to produce low molar mass, hydroxyl telechelic polyesters is most effective when conducted at minimal catalyst loading and with a controllable but excess quantity of diol, as a chain transfer agent (CTA) (Figure 1).^32−34^ The diols are proposed to undergo proton exchange, or chain transfer reactions, with the alkoxide-chain intermediates to liberate “free” polymer chains end-capped with alcohols. These chain transfer reactions usually occur faster than chain propagation, producing polyesters where the overall molar mass values depend upon the amount of diol added to the reaction.^35,36^ Unfortunately, many catalysts become inactive or show severely diminished rates when applied with excess diols or other protic chain transfer agents.^13−15^ These issues arise from side reactions between the catalyst and alcohol, including ligand protonolysis, competitive substrate inhibition through noncovalent hydrogen-bonding interactions (with the diol hydroxyl moieties), or by competitive alcohol coordination to the active center.^37^ Few catalysts tolerate excess alcohol being present, i.e., operate at equivalent rates to when it is absent, and performance evaluations are typically conducted at rather low excesses of alcohol, e.g., ∼10 equiv vs catalyst, which is often insufficient for adequate polymer chain end-group control and limits access to the oligomers.^33,38−40^ In 2020, Coates and co-workers reported a significant breakthrough, contrasting a new catalyst with a bicomponent catalyst [(salph)Al(III)(Cl)]/[CyPr]Cl system (**A**) which showed 4× lower activity, from TOF = 49 to 12 h^–1^, when 20 equiv of carboxylic acid was added ([cat]/[CTA]/[carbic anhydride (CPMA)]/[PO] = 1:50:1200:6000, CTA = 1-adamantanecarboxylic acid, *T* = 60 °C).^37^ On the other hand, the new single component catalyst [(salph[CyPr])Al(III)(Cl)](Cl) (**B**), featuring a covalently tethered cocatalyst, maintained its high TOF of 80 h^–1^ even when using 35 equiv carboxylic acid ([cat]/[CTA]/[CPMA]/[PO] = 1:1200:6000, CTA = 1-adamantanecarboxylic acid, *T* = 60 °C).^37^ The authors proposed that the tethering strategy serves to “hold” the anionic propagating chains close to the metal center, preventing deactivation by protonation.^17^ Another significant result from Chen et al. demonstrated that an Al(III)(bipyridine bisphenolate)(Cl)/PPNCl (**C**) catalyst maintained ∼85% of its rate when applied with up to 100 equiv of maleic acid.^41^ In 2022, Fieser and co-workers reported another stand-out catalyst: a hydrated YCl_3_(H_2_O)_6_/PPNCl (**D**) catalyst system showed a pronounced increase in activity over an anhydrous variant (i.e., YCl_3_(THF)_3_/PPNCl), under similar reaction conditions, a useful result since it allowed for use of unpurified monomers and the polymerization to be carried out in air.^24^

**Figure 2:**
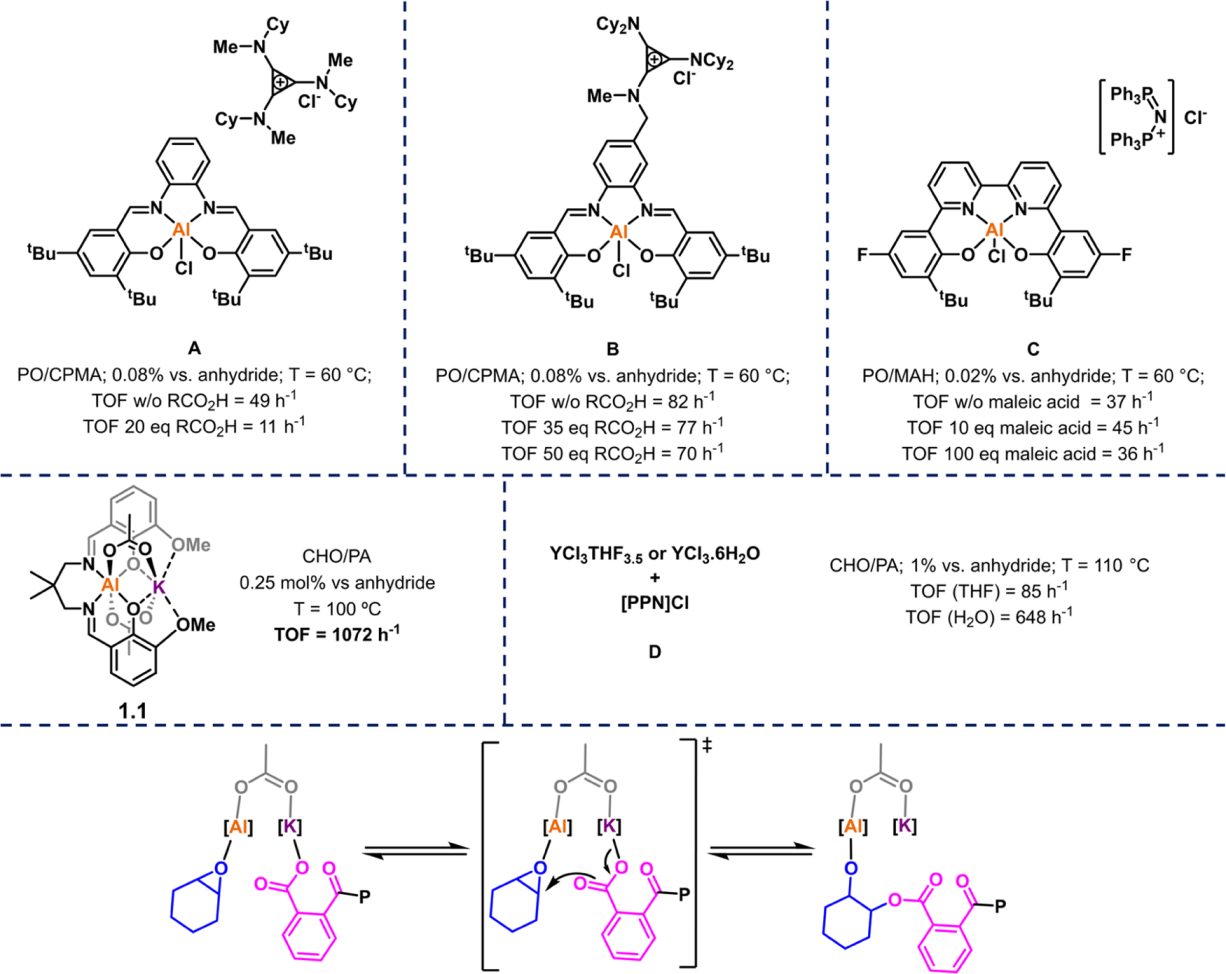
Catalysts (**A**–**D**) and **1.1**. Bottom section illustrates the proposed rate-determining step for epoxide/anhydride ROCOP using dinuclear catalyst **1.1**.

Dinuclear metal catalysts also show promise in epoxide/anhydride ROCOP; these catalysts operate without any additional cocatalysts and, by appropriate metal selection, intermetallic synergy can enhance activity.^16,30,42^ Here, dinuclear catalysts are targeted with the constraints that ligands should be straightforward to synthesize and the metals, Al(III) and K(I), should be earth-abundant, low/nontoxic, and inexpensive.^43^ Our team recently reported the first such Al(III)/M(I) catalyst, coordinated by a diphenolate Schiff base ligand, with the complex where M = K(I), **1.1**, showing high rates and selectivity in cyclohexene oxide (CHO)/phthalic anhydride (PA) ROCOP (Figure 2).^16,30^ The Al(III)/K(I) catalyst, **1.1**, achieved a TOF of 1072 h^–1^ (Catalyst/PA/CHO = 1:400:2000, *T* = 100 °C) and showed a rate law which is first order in catalyst and epoxide concentrations, i.e., Rate = *k*_p_[L_van_Al(III)K(I)(OAc)_2_][CHO]. The rate law, supported by experiments and density functional theory (DFT) calculations, was rationalized by a dinuclear metalate polymerization mechanism.^16^ The rate-determining step (r.d.s.) involves the Al(III)-epoxide intermediate being attacked by a “transient” K-carboxylate intermediate to generate an aluminate-alkoxide resting state after the insertion (Figure 2). This proposed mechanism underpins the current investigation in which a series of Al(III)/K(I) catalysts, applying systematically varied ligands, are selected to understand structural influences over activity, ester linkage selectivity, and polymerization control. Reaction conditions are targeted to test catalyst tolerance to chain transfer agents and, for the best catalysts, to produce hydroxyl telechelic polyester polyols, i.e., polymerizations will be conducted using variable but excess quantities of diol (vs catalyst). Finally, the most active and selective catalysts are tested using a range of monomers to produce diverse polyester polyol structures.

## Results and Discussion

**Figure 3:**
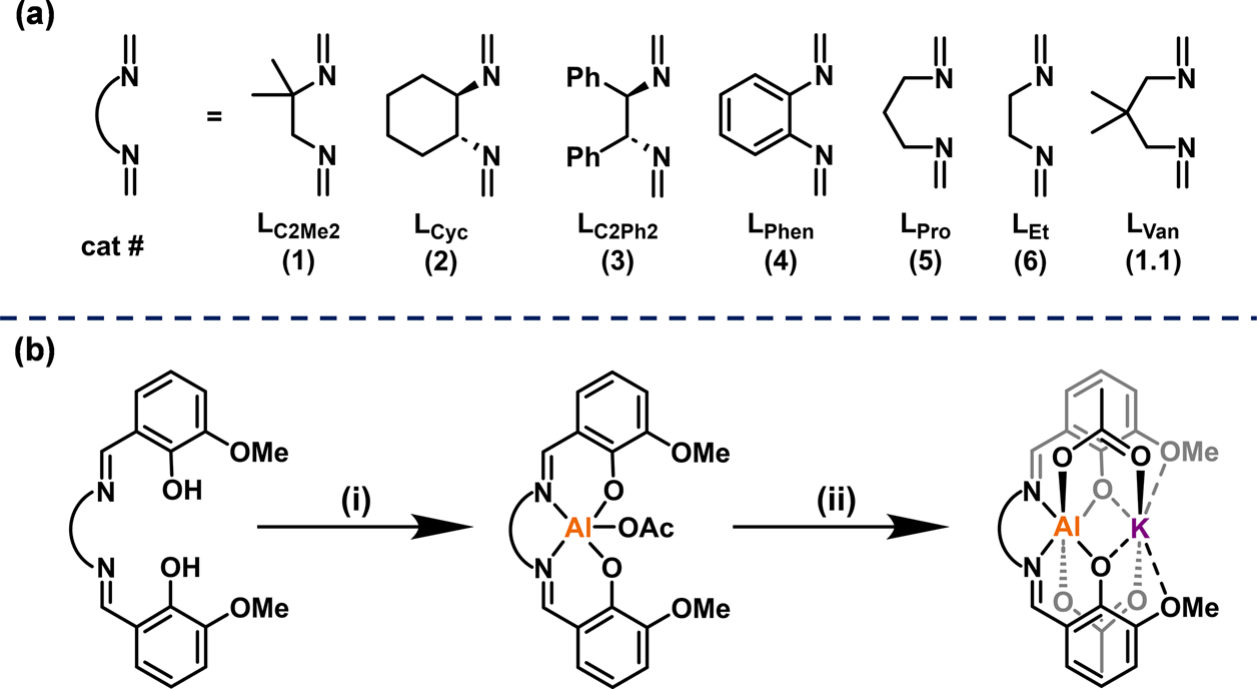
(a) Series of Al(III)/K(I) heterodinuclear catalysts differing by imine linker groups. (b) Catalyst synthesis. Reaction conditions: (i) 1.05 equiv AlEt_3_, toluene, 20 h, 25 °C, then 1.0 equiv AcOH, toluene, 12 h, 100 °C, then 12 h, 25 °C. Yields = 62% (**1**), 80% (**2**), 65% (**3**), 68% (**4**), 80% (**5**), 28% (**6**). (ii) 1.0 equiv of KOAc, CHCl_3_, 20 h, 25 °C. Yields = 99% (**1**), 99% (**2**), 99% (**3**), 93% (**4**), 88% (**5**), 93% (**6**).

To understand the catalyst structure–performance relationships, the ligand backbone was systematically varied. The “imine” linker groups were selected due to their proximity to the active site, Al(III), allowing them to impart electronic and steric influences, which should moderate both ground and transition state energies in the rate-determining step. Catalyst **1.1** features a 2,2-dimethylpropylene linker and serves as a point of comparison with a series of *C*_2_: 2,2-dimethylethylene (**1**), (1*R*,2*R*)–(−)-1,2-cyclohexylene (**2**), (1*R*,2*R*)–(−)-1,2-diphenylethylene (**3**), 1,2-phenylene (**4**), ethylene (**6**), and *C*_3_, 1,3-propylene (**5**), linker groups (Figure 3a).

The ligands were all successfully synthesized by Schiff base condensation reactions between *ortho-*vanillin and the different diamines (see the Supporting Information (SI) for experimental details).^30^ The Al(III)/K(I) catalysts, **1**–**6**, were prepared by first reacting the ligand with triethylaluminum and then adding an equivalent of acetic acid to yield the Al(III) acetate complex which was isolated. Subsequent addition of one equivalent of KOAc yielded the air-stable Al(III)/K(I) heterobimetallic complex (Figure 3b).^30^

Catalysts **1**–**6** were isolated as yellow powders in good yields and were characterized by ^1^H, ^13^C, and ^27^Al NMR spectroscopy and, where relevant, by single-crystal X-ray diffraction (Figures S1–S42). Further confirmation of identity and purity was achieved by infrared (IR) spectroscopy, matrix-assisted laser desorption ionization time-of-flight (MALDI-ToF) spectroscopy, and elemental analysis (Figures S43–S54). All catalysts show NMR chemical shifts consistent with the successful formation of heterodinuclear complexes. For example, the formation of complex **1** (2,2-dimethylethylene) was monitored by the disappearance of the ligand phenol peaks (13.75 and 14.37 ppm) upon reaction with triethylaluminum, followed by a new acetate resonance (1.87 ppm) upon reaction with acetic acid (Figure S2). After the final step, the increased relative acetate peak integral (6H) is consistent with the complex having two acetate ligands. The acetate ligands show a singlet resonance, which indicates that the complex adopts a structure where the acetates are equivalent on the NMR time scale. The acetate peak is also observed at a low chemical shift, as expected for a μ_2_- κ1- binding mode (vide infra). The ^27^Al NMR spectra of each complex show a broad singlet, from 0–10 ppm, consistent with a hexacoordinate Al(III), albeit with some asymmetry in its coordination chemistry (Figures S5, S11, S18, S25, S32, and S39).

**Figure 4:**
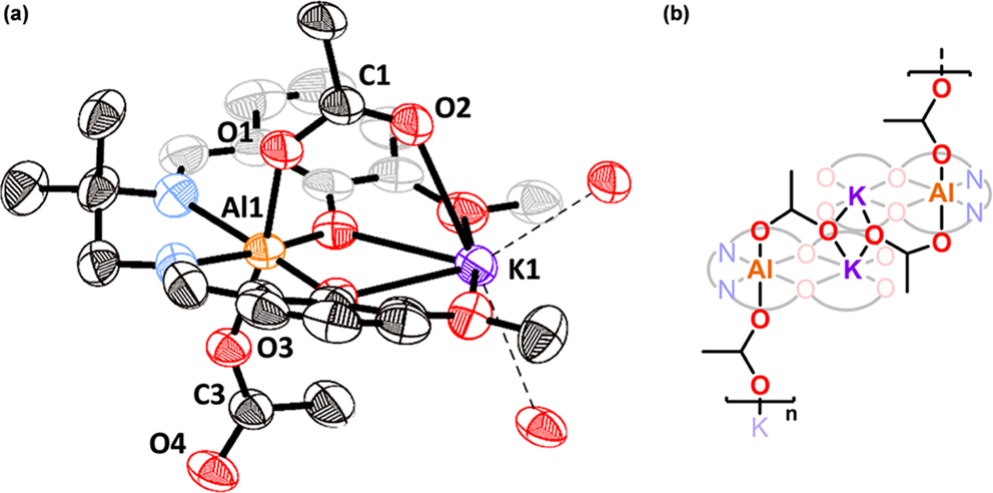
(a) Molecular structure of complex **1** obtained from X-ray diffraction experiments, with thermal ellipsoids presented at 50% probability and H atoms omitted for clarity (atom color scheme: Al (orange), K (purple), O (red), N (blue), and C (black/grayscale)). Structure is polymeric; the repeat unit (monomer) is presented. (b) Schematic showing a repeat unit of the polymeric structure.

X-ray diffraction experiments were conducted using single crystals prepared by vapor diffusion of hexane into chloroform solutions (Table S1). In the solid state, complex **1** forms an extended polymeric structure in which each Al(III) center is hexacoordinate, within a distorted octahedral coordination geometry, consistent with the observed ^27^Al NMR spectrum (Figures 4 and S5). The Al(III) is speciated as an aluminate, with the two acetate oxygen atoms being coordinated anionically to it. The aluminate speciation was confirmed by the different acetate ligand C–O bond lengths [O(1)–C(1) = 1.271(3) Å, O(2)–C(1) = 1.226(3) Å, O(3)–C(2) = 1.273(2) Å, O(4)–C(2) = 1.229(3) Å] (Table S1). The Al–O bonds are considerably shorter than the K–O bonds, consistent with the larger ionic radius of the latter [K(1)–O(2) = 2.6641(18) Å, K(1)–O(4) = 2.9294(18) Å, Al(1)–O(1) = 1.9174(17) Å, and Al(1)–O(3) = 1.8915(15) Å] (Table S1). The Al(1)–K(1) separation is 3.5789(8) Å, similar to that reported for **1.1**, which demonstrated intermetallic synergy in the catalysis.^30^

## Epoxide/Anhydride ROCOP Catalysis

Complexes **1**–**6** were each tested as polymerization catalysts and, to allow comparisons with the broader literature, the commonly used “benchmark monomers” phthalic anhydride (PA) and cyclohexene oxide (CHO) were selected (Table 1).^24,28,44^ Polymerizations were conducted using [cat]/[PA]/[CHO] of 1:100:500 and at 100 °C, allowing comparison to catalyst **1.1**.^30^ The polymerizations were monitored by regular removal of aliquots, which were used to determine the conversion (TON), selectivity (% ester linkages), and molar mass (*M_n_*, *Đ*) vs time relationships. While these tests were run under an N_2_ atmosphere, the catalyst is air-stable, and polymerizations are successful in air.

**Table 1:**
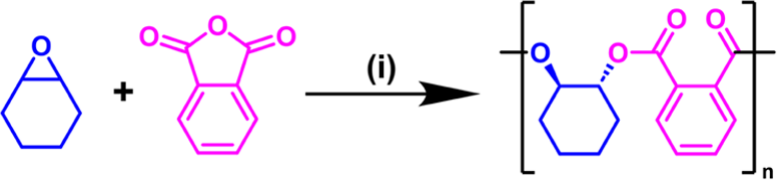
Polymerization Data for CHO/PA ROCOP with Catalysts **1**–**6;** Polymerization Conditions: (i) [Cat]_0_/[PA]_0_/[CHO]_0_ = 1:100:500, *T* = 100 °C

	conditions		polymerization results				
entry	cat	*t* (min)	conv. (%)	TOF (h^–1^)^a^	PE selectivity (%)^b^	*M*_*n*,GPC_ (g mol^–1^) [*Đ*]^c^	*M*_n,theory_ (g mol^–1^)^d^
a	**1** (L_C2Me2_)	10	74	440	>99	6000 [1.13]	9200
b	**2** (L_Cyc_)	15	69	280	>99	6200 [1.13]	8500
c	**3** (L_C2Ph2_)	15	64	260	93	4900 [1.15]	7900
d	**4** (L_Phen_)	90	69	46	59	3500 [1.57]	8500
e	**5** (L_Pro_)	10	60	360	93	2100 [1.13]	4400
f	**6** (L_Et_)	15	83	330	>99	5000 [1.26]	10,200
g^e^^,^^30^	**1.1** (L_Van_)	15	67	1070	>99		

a Turnover frequency = TON/time(h). TON determined via Conversion*[monomer] (where [] is the equivalence). Conversion determined by ^1^H NMR (298 K, 400 MHz, CDCl_3_) spectroscopy; comparison of PA monomer peaks (8.06–8.00 and 7.94–7.88 ppm) to polymer peaks (7.63–7.52 and 7.44–7.34 ppm) (Table S2 and Figure S55).

b Selectivity for polyester over ether linkage formation. Determined by ^1^H NMR spectroscopy (298 K, 400 MHz, CDCl_3_) by comparison of polyester peaks (5.22–5.04 ppm) vs any ether linkages (3.8–3.2 ppm).

c Determined by GPC in THF, 30 °C, calibrated using PS standards.

d Determined according to *M*_*n*,theory_ = (TON × *M*_n,repeatunit_)/(2 × [catalyst]) + *M*_*n*,OAc_.

e Loading at [cat]_0_/[PA]_0_/[CHO]_0_ = 1:400:2000.

Catalysts **1**, **2**, and **6** showed quantitative ester linkage selectivity and high activities, with TOF(**1**)= 440 h^–1^, TOF(**2**) = 280 h^–1^, and TOF(**6**) = 330 h^–1^; catalyst **1** had the highest activity of the series (Table 1a,b). Catalysts **3** and **5** were also active, with TOFs of 260 h^–1^ and 360 h^–1^, respectively; however, both showed slightly lower ester linkage selectivity (93%, Table 1c,e). Catalyst **4** showed the lowest rate and selectivity (Table 1d), and the low ester selectivity was consistent at all conversions. Curiously, heating it in neat epoxide did not yield any polyether. Further, its selectivity was dependent on catalyst loading, but the resulting polymer DOSY NMR spectrum revealed that a single copolymer species was formed; these findings indicate alternative monomer sequences during enchainment (Table S3 and Figure S56).^45^ The more selective catalysts, **1**–**3**, **5**, and **6**, all showed very good polymerization control with reasonable agreement between theoretical and experimental molar mass values; all of the polyesters showed narrow molar mass distributions.

We observe that increasing the backbone linker rigidity reduced the catalytic rate and selectivity, as apparent from comparing the activity for **1**, **2**, and **4** (Table 1a,b,d). A similar conclusion can be drawn when comparing C_2_ and C_3_ backbone activity, where we see **1.1**, the C_3_ backbone, is over twice as fast as **1**, the C_2_ backbone (Table 1a,g).^30^ Indeed, previously in lactide ROP, differences in rates between catalysts were often attributed to ligand rigidity.^46,47^

Comparing the most active Al(III)/K(I) catalysts, **1**, **5**, **6**, and **1.1**, showed that imine linkers featuring two methyl groups improved both polymerization activity and selectivity. Catalyst **1** (1,1-dimethylethylene backbone) showed the highest rates of the complexes featuring C_2_-backbones; it was 1.3 times faster than **6** (ethylene backbone). Further, the methyl substituent rate enhancement was also observed for the C_3_ backbone catalysts, where the catalyst **1.1** (2,2-dimethylpropylene) was 3 times as fast as **5** (1,3-propylene linker) (Table 1a,e–g).^30^ To investigate whether this trend was also related to ligand conformational fluxionality, VT NMR spectroscopy was conducted using catalysts **1.1** and **5** (Figures S57 and S58). Both complexes show average ligand backbone conformations, particularly focused on the ligand-Al(III) six-membered ring at room temperature. On cooling to −80 °C, both samples gave the same response to temperature and still showed broad peaks with no peak splitting that would indicate a freezing out of ligand fluxionality. Thus, it appears more likely that the methyl substituents influence the electron density at the Al(III) active site as a means of mediating the catalytic activity.

**Figure 5:**
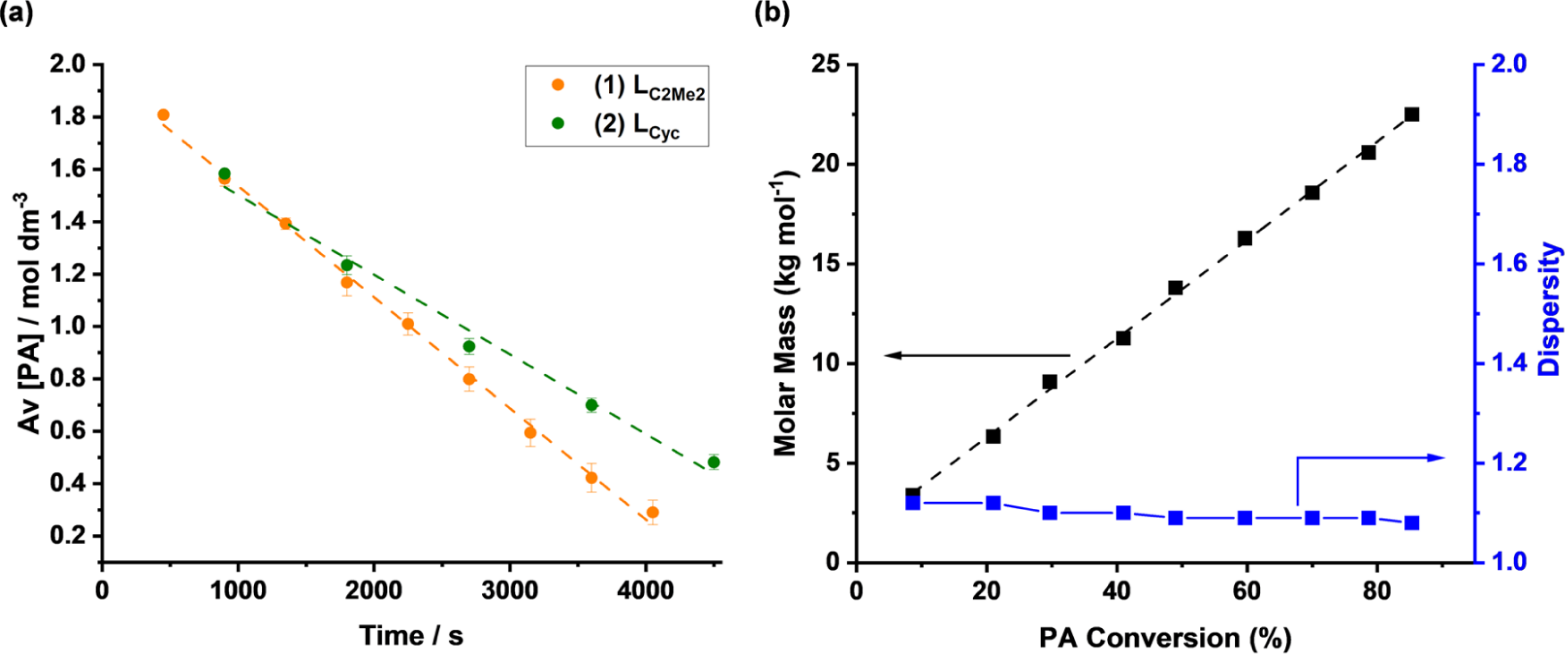
Kinetic analysis of PA/CHO ROCOP with catalysts **1** and **2**. Conditions: [cat]/[PA,]/[CHO] = 1:400:2000, *T* = 100 °C. Reactions carried out in triplicate, with aliquots at regular time intervals. (a) Graph of [PA] against time for CHO/PA ROCOP using catalysts **1** (orange) and **2** (green). (b) Plot of the change in polymer molar mass and dispersity with PA conversion for catalyst **1**.

Complexes **1** and **2** are two of the best-performing catalysts from this series and hence were applied at lower loadings (catalyst/PA/CHO = 1:400:2000, *T* = 100 °C) with conversions and molar mass data vs time correlated (Figures 5, S59, and S60). Catalyst **1** maintained its good performance, and when monitoring the rate of anhydride consumption, showed a constant, high pseudo zero order rate constant, *k*_obs_ (calculated from linear plots of [PA] vs time, *k*_obs_ = 4.3 × 10^–4^ mol dm^–3^ s^–1^, Figure 5a). Complex **2** showed slightly better loading tolerance but was slower than that of **1**, with *k*_obs_ = 2.1 × 10^–4^ mol dm^–3^ s^–1^ under equivalent conditions (Figure S59). The polyester molar mass values, determined from aliquots analyzed by GPC, showed a linear increase vs conversion/time and narrow, monomodal distributions throughout the reaction (Figure 5b). These findings are consistent with well-controlled/living polymerizations.

**Figure 6:**
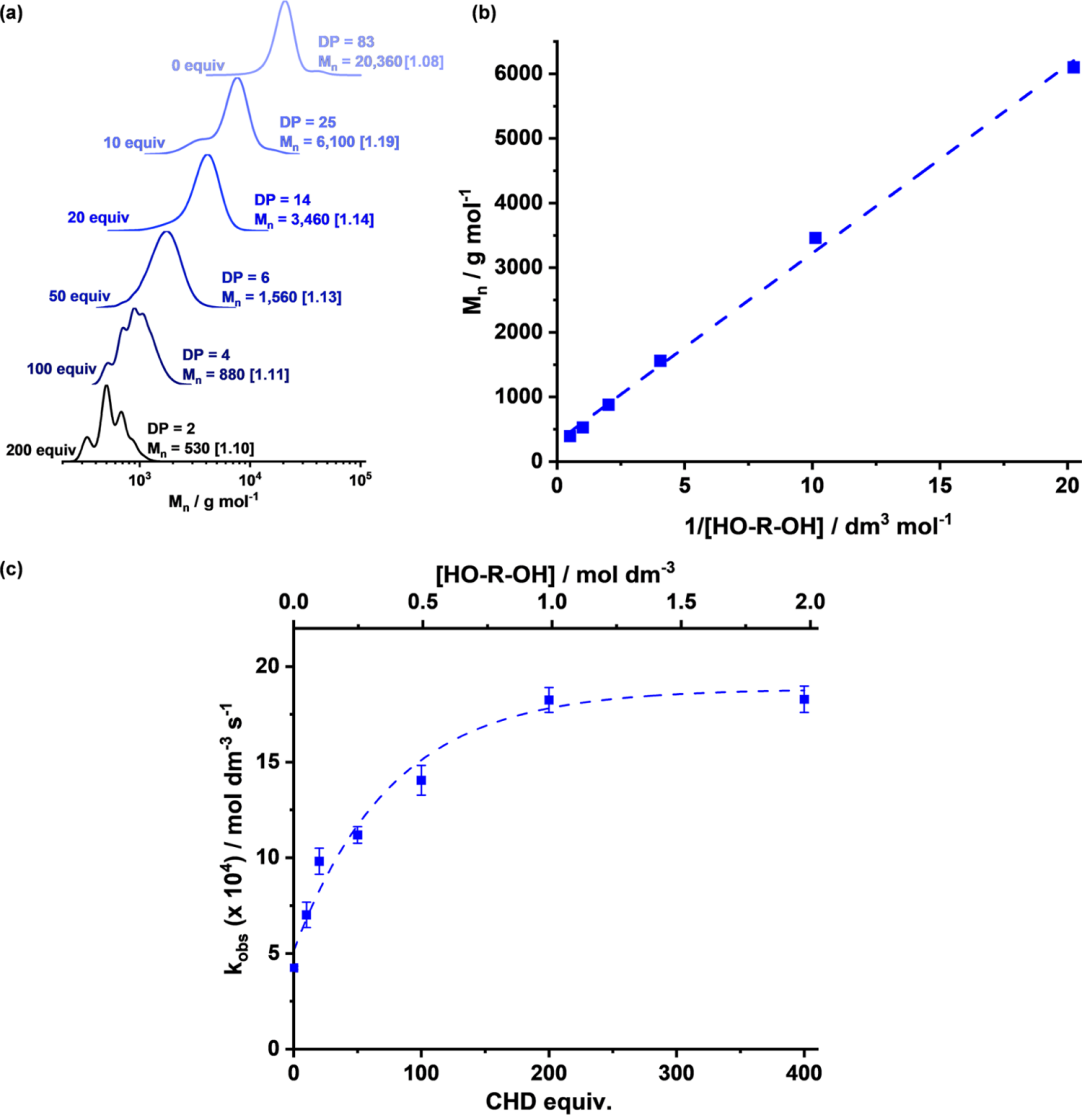
Analysis of polymerization control and activity for CHO/PA ROCOP was conducted using variable quantities of diol (CHD) with catalyst **1**. Conditions: [**1**]/[CHD]/[PA]/[CHO] = 1:10–400:400:2000, at 100 °C. (a) Evolution of molar mass, at full PA conversion, vs the amount of diol added (10–200 equiv) as illustrated by the GPC data. (b) Plot of Polyester *M_n_* at full conversion vs [HO-R–OH]. (c) Plot of *k*_obs_ vs [HO-R–OH] and vs # CHD equivalents. Errors were derived from fits of [PA] (mol dm^–3^) vs *t* (s) data (Table S4).

To target the production of polyester polyols, the lead catalyst (**1**) was tested for PA/CHO ROCOP conducted with controlled quantities of diol, *trans-*cyclohexane-1,2-diol (CHD), as the chain transfer agent. The CHD was added at 10–400 equiv vs catalyst, and all reactions were conducted with the removal of regular reaction aliquots ([**1**]/[CHD]/[PA]/[CHO] = 1:10–400:400:2000, *T* = 100 °C, Figure 6, Tables 2 and S4). All polymerizations were run until complete anhydride consumption, and linear plots of [PA] vs time indicated zeroth orders in anhydride concentration and allowed for determination of both point TOF values and rate constants, *k*_obs_ (Figure S61).

**Table 2:** Data for CHO/PA ROCOP with Variable Loadings of Diol (CHD) Using Catalyst **1^a^**

entry	CHD equiv (#)	*t* (min)	TON^b^	activity, TOF (h^–1^)^c^	*k*_obs_ (mol dm^–3^ s^–1^ × 10^–3^)^d^	*M*_*n*,GPC_ (g mol^–1^) [*Đ*_M_] at full conversion^e^	*M*_*n*,theory_ (g mol^–1^)^f^
a	0	30	213	426	0.425	20,000 [1.09]	49,000
b	10	15	180	730	0.702	6,100 [1.19]	8300
c	20	15	232	926	0.982	3,500 [1.14]	4600
d	50	15	311	1240	1.12	1,600 [1.13]	2000
e	100	10	284	1700	1.41	880 [1.11]	1100
f	200	10	315	1890	1.83	530 [1.12]	600
g	400	5	145	1740	1.83	400 [1.06]	360

a Polymerization conditions are [**1**]/[CHD]/[anhydride] = 1:#:400, using 1 mL CHO such that [**1**] = 4.9 mmol, 100 °C.

b TON = (conversion (%)/100 (%)) × ([anhydride]/[**1**]). Conversion determined by ^1^H NMR spectroscopy (298 K, 400 MHz, CDCl_3_) by comparison of the normalized integrals of PA peaks, at 8.06–8.00 and 7.94–7.88 ppm, vs equivalent polymer peaks, 7.63–7.52 and 7.44–7.34 ppm.

c Turnover frequency = TON/time(h). TON determined by conversion × [monomer].

d Determined as the gradient of fits to plots of [PA] (mol dm^–3^) vs time (s).

e Determined by GPC, in THF at 30 °C, with the instrument calibrated using PS standards.

f Determined using *M*_*n*,theory_ = (TON × *M*_n,repeatunit_)/(2 × [catalyst] + [CHD]) + *M*_*n*,CHD_.

The polymer molar masses were inversely proportional to the quantities of diol added, once again consistent with a well-controlled polymerization (Figure 6a,b).^34^ For polymerizations with only catalyst, initiation occurred primarily from the acetate ligands, but when diol (CHD) was added, initiation also occurred from the alcohol groups. Adding progressively greater quantities of CHD into the polymerizations resulted in the polyester molar mass being reduced, but the low dispersity of the distributions was maintained. After 20 equiv CHD, a single, monomodal distribution was observed, consistent with the majority of chains being diol initiated, as confirmed by MALDI-ToF analysis (Figures 6a, S62, and S63).^40,48−51^ The catalyst showed unusually high tolerance to the chain transfer agent, allowing access to oligomers, with degrees of polymerization (DP) < 10 and with experimental molar mass values in very good agreement with theoretical values. The polymerization catalyst remained active and controlled using 200 equiv of diol, and under these conditions, the resulting oligomer had an average DP of just 2. It is notable that the distinct chain lengths are distinguished by GPC in this molar mass regime (Figure 6a). A plot of polyester molar mass vs 1/[diol] showed a linear fit, consistent with the outstanding polymerization control exhibited by catalyst **1** (Figure 6b). The data demonstrate that the catalyst is very well suited to the production of the low molar mass polyesters required for liquid formulations, in every case showing excellent control and predictable molar mass values.

Surprisingly, the polymerization rates and TOF values also correlated with the quantity of diol, and rates increased as progressively greater quantities of diol were added (Figure 6c and Table 2). Accordingly, adding 50 equiv of alcohol resulted in ∼3× higher activity, and adding 200 equiv of alcohol resulted in ∼4.5 rate enhancement compared to the reaction run without any added alcohol, reaching a TOF of 1890 h^–1^. The ester linkage selectivity remained at >99%, and in all cases, perfectly alternating polyesters were produced (Table S4).

In order to investigate the beneficial effects of diol addition upon the polymerization rate, it is important to consider whether any changes to the overall solution viscosity could be responsible. Generally, during polymerizations, the viscosity increases, and at higher conversions, reactions may become diffusion-controlled. In this work, analysis of the polymerization aliquots showed that rates remained constant throughout polymerizations (from 10–80% conversion), even when producing high molar mass polyesters (Figure S61). In each polymerization, the degree of polymerization increased with conversion; thus, rates were not influenced by any changes in viscosity with increasing chain length. In addition, comparing polymerizations conducted with different diol concentrations but producing polyesters with the same DP showed different rates. For example, a polymerization using **1** without any diol was stopped at 9% conversion, resulting in a polyester with DP = 14 and TOF = 277 h^–1^. The same reaction was conducted using 10 equiv of diol and stopped at 46% conversion, resulting in the same polyesters with DP = 11 but showing a much higher TOF of 730 h^–1^ (Figure S64). Since the polymer solution viscosity should be the same or very similar, in both cases, the rate enhancements when alcohol is added do not relate to viscosity effects (Figure S65).

Catalyst **1** was analyzed by DOSY NMR spectroscopy in methanol-d^4^ at room temperature; the hydrodynamic radius obtained is consistent with it remaining dimeric in solution (Figure S66). The ROCOP reactions are conducted at higher temperatures (100 °C), which may favor (entropically) dissociation to monomers under the conditions of the catalysis, but at this point, we cannot rule out a catalytically active dimer either.

Next, the influences of diol upon the polymerization rate were investigated using another catalyst in the series, **1.1** (featuring a 2,2-dimethylpropylene backbone) (Table S5). Once again, the catalytic activity increased with alcohol addition, although the magnitude of the increase was lower than that for catalyst **1**. In the case of **1.1**, polymerizations were ∼1.6× faster when 100 equiv of diol were added, whereas using **1**, under the same conditions, resulted in a 4× greater TOF (Tables 2a,d and S5a,c). These experiments indicate that a “molecular” rationale for the rate increases since the resulting polymer chains are identical (Tables 2 and S5). Plotting rates vs quantity of alcohol shows that rates increase but eventually plateau (for **1**) or even decrease slightly (for **1.1**), but it is important to appreciate that rates level off when using an equimolar amount of alcohol and anhydride monomer. These differences in rate response to alcohol loading do not reflect a difference in stability to alcohol, since both catalysts were stable at 100 °C for 30 min in the presence of macromolecular triol, glycerol ethoxylate (Figures S67–S70).

This means that the limits of the catalysis occur when only a single ring-opening reaction occurs, yielding materials with DP = 1. Under conditions where useful polyols are produced, i.e., using 10–100 equiv alcohol, the rates increase significantly with alcohol concentration for both catalysts.^37,41^

## Monomer Scope

Catalyst **1** was tested by using different monomer combinations to produce a range of polyester polyols (Table 3). Vinyl cyclohexene oxide (vCHO) and allyl glycidyl ether (AGE) were selected to feature alkene moieties which could, in future, be postfunctionalized to moderate hydrophilicity, pH, and rheology or to install specific functional groups or ions.^52^ All of these features could be relevant to future applications in liquid formulations. Diglycolic anhydride (DGA) is a biobased monomer producing aliphatic polyesters with flexible backbones and low glass-transition temperatures.^25^ Rigid, biobased monomers norbornene anhydride (NBA)^53^ and tricyclic anhydride (TCA)^54^ were selected for the opposite reason—they yield polyesters with more “rigid” backbones and higher *T*_g_ values. Finally, propylene oxide (PO) was used since it is one of the most widely produced epoxides globally, and its polymers are already widely applied in surfactant applications.^55^ In all cases, epoxide/anhydride ROCOP was conducted using 20 equiv of diol (CHD) to produce hydroxyl telechelic polyesters with DP values from 11–22, and molar mass values in the polyol range (2–5 kg mol^–1^), in most cases with narrow distributions.^8^

**Table 3:**
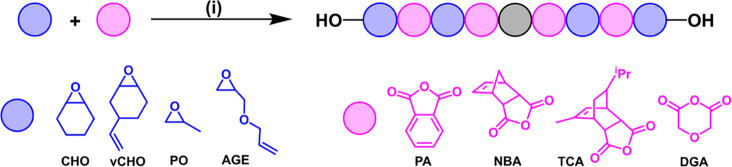
Data for Epoxide/Anhydride ROCOP Using Catalyst **1** (the Structures and Abbreviations for the Monomers are Shown Below)^a^

	conditions		polymerization results				
entry	monomers	time (min)	TON^c^	activity TOF (h^–1^)^d^	polyester selectivity (%)^e^	*M*_*n*,GPC_ (g mol^–1^) [*Đ*_M_] at full conversion^f^	*M*_n,theory_ (g mol^–1^)^g^
a	CHO/PA	15	232	926	>99	3500 [1.14]	4500
b	vCHO/PA	15	225	901	>99	4600 [1.11]	5000
c^b^	PO/PA	420	182	26	>99	4700 [1.13]	3800
d	AGE/PA	30	172	343	>99	4500 [1.15]	4800
e	CHO/NBA	30	121	243	>99	3600 [1.11]	4800
f	CHO/TCA	30	128	256	>99	3700 [1.15]	6000
g	CHO/DGA	15	212	846	>99	2600 [1.32]	3900

a Polymerization conditions: (i) [**1**]/[CHD]/[anhydride] = 1:20:400, 1 mL epoxide such that [**1**] = 4.9 mmol, 100 °C.

b Reaction conducted at 60 °C.

c TON = (conversion (%)/100 (%)) × ([anhydride]/[**1**]). Conversion determined by ^1^H NMR spectroscopy (298 K, 400 MHz, CDCl_3_) by comparison of the normalized integrals for PA peaks, 8.06–8.00 and 7.94–7.88 ppm, and polyester peaks, 7.63–7.52 and 7.44–7.34 ppm.

d Turnover frequency = TON/time (h). TON determined via conversion × [anhydride].

e Determined by ^1^H NMR spectroscopy (298 K, 400 MHz, CDCl_3_) by comparison of resonances due to polyester, 5.22–5.04 ppm, vs any ether linkages, 3.8–3.2 ppm.

f Determined by GPC, in THF at 30 °C, calibrated using PS standards.

g Calculated from (TON × *M*_*n*,repeatunit_)/[catalyst + diol].

All monomer combinations were successfully enchained, producing polyesters with molar mass values in close agreement with the theoretical values; slight variations arise since the values are determined by GPC measurements calibrated using polystyrene standards (Figures S71–S84). All monomer combinations were polymerized to yield perfectly alternating polyesters, without any ether linkages, within the detection limits of NMR spectroscopy (Table 3). Catalyst **1** showed high activity for ROCOP using vCHO/PA or CHO/PA or CHO/DGA, reaching TOF values of ∼900 h^–1^ (Table 3a,b,g). The rates were slightly slower using alkylene/glycidyl ether oxides, particularly where lower temperature conditions were necessary due to monomer volatility (TOF(PO) = 26 h^–1^ at 60 °C; TOF(AGE) = 343 h^–1^ at 100 °C, Table 3c,d). The resulting polyesters showed regiorandom distributions, as determined by quantitative ^13^C NMR spectroscopy (Figure S85). The catalyst was also active for sterically hindered anhydrides, showing activity values within the range expected for such monomers, e.g., CHO/NBA (TOF = 243 h^–1^) and CHO/TCA (TOF = 256 h^–1^, Table 3e,f).

It is also notable that there were, once again, rate enhancements using the different monomer combinations through the addition of the diol. For example, vCHO/PA ROCOP conducted using catalyst **1** with 20 equiv of alcohol showed 5× greater activity compared to the equivalent polymerization conducted without any diol (Table S6a,b). PO/PA ROCOP showed a 1.4× rate increase when 20 equiv of alcohol were used compared to reactions without any diol (Table S6c–e).

## Discussion

The increases in rates when diols are added to the polymerizations were manifested using different catalysts and various monomer combinations. These findings suggest that the rate increments arise from changes at the catalyst active site. It is usually found that catalyst activity is compromised upon addition of chain transfer agents (alcohols or acids), but a few catalysts maintained their activity or even showed a slight rate enhancement for epoxide/anhydride ROCOP (Figure 1).^17,37,38,40,41,51^ For example, Coates and co-workers reported a high-performance [(salph[CyPr])Al(III)(Cl)](Cl) catalyst (**B**) which showed excellent activity in PO/CMPA ROCOP (0.08 mol %, 60 °C) (Figure 1).^37^ Using a loading of 1:1250 = cat/CMPA resulted in a TOF of ∼82 h^–1^, with the activity maintained (77 h^–1^) in the presence of 35 equiv of carboxylic acid as chain transfer agent (Figure 2). In this work, using PA instead of CMPA, at a higher catalyst loading but at equivalent temperature (0.25 mol % catalyst, 60 °C), catalyst **1** was less active with a TOF of 20 h^–1^ increasing to 28 h^–1^ when 20 equiv of alcohol were added. Catalyst **1** was also slightly less active than the impressive Al(III)(bipyridine bisphenolate)(Cl)/PPNCl (**C**) catalyst, reported by Chen et al., which showed a TOF of 36 h^–1^ for PO/CMPA at very low loadings and with 100 equiv of acid (1:5000 = catalyst/CMPA, 60 °C).^41^ Comparatively, catalyst **1** was slower but showed an increase in activity when excess alcohol was added, which is different to catalyst **C**. The YCl_3_(THF/H_2_O)_3_/PPNCl catalyst systems (**D**), reported by Fieser and co-workers, showed high activity values, which increase for the aqua complex compared with the THF adduct.^24^ The best catalyst achieves an activity of 648 h^–1^ for CHO/PA ROCOP (1 mol % catalyst, 110 °C). Using the same monomers (CHO/PA), at lower loading and slightly lower temperature, catalyst **1** showed activity values from 426 h^–1^, without any alcohol, rising to 1890 h^–1^ when 200 equiv of alcohol were added (0.25 mol % catalyst, 100 °C). Overall, the Al(III)K(I) catalysts operate without cocatalyst and show alcohol-enhanced rates with a range of different epoxides. Their absolute performances (activity/selectivity/loading) are equivalent to these “best-in-class” literature catalysts, but the consequences of alcohol additions upon rates appear somewhat distinctive to those known catalysts.

**Figure 7:**
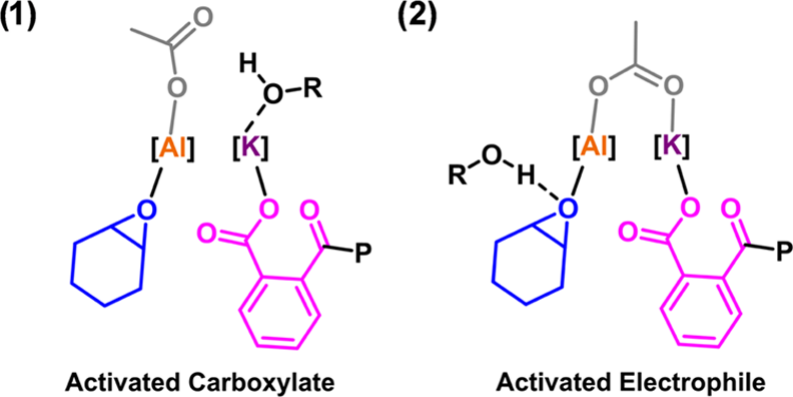
Illustration of the structures of possible catalytic intermediates that may account for the alcohol-enhanced rates of epoxide/anhydride ROCOP catalyzed by the Al(III)/K(I) complexes (the ancillary ligand chemistry is not illustrated for clarity).

The rate law and dinuclear metalate mechanism were previously determined for catalyst **1.1** and are proposed to be relevant to catalyst **1**.^30^ As such, the polymerization rate-determining step is proposed to involve an Al(III)-epoxide complex being attacked and ring-opened by a K-carboxylate species. In order to rationalize the rate enhancements with alcohols, two different “catalytic activation” processes could be responsible: (1) coordination of the alcohol at the K(I) site, which enhances the K(I)-carboxylate nucleophilicity; (2) Al(III)-epoxide species interacting with an “outer sphere” alcohol, which activates it to ring-opening (Figure 7). The K(I) coordination should be favored by its Lewis acidity and large size, which allow the ion to coordinate additional donor molecules. Indeed, X-ray crystallography experiments showed K(I) coordination when using THF as the crystallization solvent.^30^ With the current data, these two activation chemistries are indistinguishable, and both may occur; future investigations will focus on investigating kinetics and isolating adducts to test the hypotheses.

In the literature, there are very few examples of alcohols accelerating the rates of similar polymerizations.^56^ In epoxide ring-opening polymerization (ROP), catalysts featuring either s-block metals or Al(III) are well known, although no catalysts yet combine the two metals, and, in both cases, alcohols decreased rates substantially.^57^ For example, commercially relevant sodium and potassium alkoxide/hydroxide catalysts for ethylene or propylene oxide ROP showed diminished rates when adding excess alcohol.^56,58^ The pioneering Al-porphyrin catalyst systems, reported by Inoue and co-workers, also showed reduced rates upon addition of alcohols.^35^ In the field of cyclic ester ROP, Hamaide and co-workers reported that excess isopropanol added to homoleptic isopropoxide complexes of Al(III), Zn(II), or Y(III) increased rates.^59^ Polymerizations conducted using 10 equiv (vs catalyst) of alcohol resulted in slight increases to activity, but when greater quantities of alcohol were added, reactions were completely inhibited. The data was rationalized by higher alcohol concentrations (above 10 equiv), resulting in competitive metal coordination blocking the active site.^59^

The ring-opening polymerization of epoxides, lactides, lactones, and cyclic carbonates can also be achieved by an activated monomer mechanism, which is reliant on the addition of alcohol.^57,60^ Ground-breaking work from Hedrick and Waymouth and co-workers established that such activated monomer mechanisms typically show rate laws that are first-order dependent upon monomer, catalyst, and alcohol concentrations. Their investigations applied *N*-heterocyclic carbene, thiourea/amine, sterically hindered amines, and Brønstead acid catalysts, among others.^61−64^ In 2016, the team reported highly active yet very controlled K(I) or Na(I) thioimidate lactide ROP catalysts, which operate by a mechanism involving both alcohol and monomer activation by the anionic thioimidate catalysts.^65^ While there may be parallels with epoxide or lactone ROP, there are also critical differences, not least the catalyst resting intermediate is a metal-carboxylate species in epoxide/anhydride ROCOP, whereas the equivalent intermediate is a metal-alkoxide in either epoxide or lactone ROP.

## Conclusions

In summary, a series of heterodinuclear Al(III)/K(I) catalysts showed very high rates, control, and selectivity for cyclohexene oxide/phthalic anhydride ring-opening copolymerizations. The catalysts showed even greater activity and equivalently high polymerization control when alcohols were added as the chain transfer agents. The catalysts and diols were used to selectively produce polyester polyols showing degrees of polymerization from 1–83 in all cases, with predictable molar mass values and monomodal, narrow dispersity, and molar mass distributions. The best catalysts showed significant rate enhancements after alcohol addition, achieving TOF values of ∼1900 h^–1^ when using 200 equiv of alcohol. This corresponds to a 4.5× increase in rates compared with reactions without the alcohol. The acceleration of rates upon the addition of excess alcohol is very unusual and contrasts with those of most other catalysts in this field. The lead catalysts were also fast, selective, and controlled in other epoxide/anhydride polymerizations, producing low molar mass, hydroxyl telechelic polyesters in all cases. These catalysts applied for different epoxide/anhydride ROCOP show significant promise in the preparation of oligomeric polyesters, which are important products for future applications in liquid formulations as well as for polyurethane and resin production.

## Supporting Information Available

Crystal data (CIF)
Experimental methods, characterization data of complexes (NMR, X-ray crystallography, IR, MS, elemental analysis) and polymerization data (GPC chromatograms, MALDI-ToF Spectra, NMR) (PDF)

## Supplementary Material

cs3c05712_si_001.cif
