# Supplementary material for: Al(III)/K(I) Heterodinuclear Polymerization Catalysts Showing Fast Rates and High Selectivity for Polyester Polyols
Source: ACS Catal. 2024 Jan 11;14(3):1363–74. doi: 10.1021/acscatal.3c05712 (PMC10845108; doi:10.1021/acscatal.3c05712)
Supplement: Supplementary file 2 — cs3c05712_si_002.pdf [file cs3c05712_si_002.pdf]

Electronic Supplementary Information

For

Al(III)/K(I) Heterodinuclear Polymerization Catalysts Showing Fast Rates and High Selectivity For Polyester Polyols

Edward J. K. Shellard[a], Wilfred T. Diment[a], Diego A. Resendiz-Lara[a], Francesca Fiorentini[a],

*Georgina L. Gregory[a], Charlotte K. Williams[a]**

[a]Chemistry Research Laboratory, Department of Chemistry, University of Oxford, 12

Mansfield Road, Oxford, OX1 3TA, U.K.

*Email: Charlotte.williams@chem.ox.ac.uk

1. General Procedures and Materials 5
2. Methods 6
3. Polymerization methods 7
3.1 Epoxide/Anhydride polymerisation 7
3.2 Polymer isolation 7
4. Synthesis of complexes 7
4.1 Synthesis of ligand (LH_2) 7
4.2 Synthesis of complex **1**, [$L_{C2Me2}AlK(OAc)_2$] 7
4.3 Synthesis of complex **2**, [$L_{Cyc}AlK(OAc)_2$] 8
4.4 Synthesis of complex **3**, [$L_{C2Ph2}AlK(OAc)_2$] 9
4.5 Synthesis of complex **4**, [$L_{Phen}AlK(OAc)_2$] 9
4.6 Synthesis of complex **5**, [$L_{Pro}AlK(OAc)_2$] 10
4.7 Synthesis of complex **6**, [$L_{Et}AlK(OAc)_2$] 10
5. Supplementary Figures and Tables 12

Figures:

Figure S1. ^{1}H NMR Spectrum of $L_{C2Me2}H_2$ ($CDCl_3$(#), 298 K, 400 MHz) 12
Figure S2 ^{1}H NMR Spectra of synthesis route for complex 1. 12
Figure S3. ^{1}H NMR Spectrum of complex 1 ($CDCl_3$(#), 298 K, 400 MHz). 13
Figure S4. ^{13}C NMR Spectrum of complex 1 ($CDCl_3$(#), 298 K, 151 MHz). 13
Figure S5. ^{27}Al NMR Spectrum of complex 1 ($CDCl_3$, 298 K, 104 MHz). 14
Figure S6. ^{1}H COSY NMR Spectrum of complex 1 ($CDCl_3$, 298 K, 400 MHz) 14
Figure S7. ^{1}H-^{13}C HSQC NMR Spectrum of complex 1 ($CDCl_3$, 298 K) 15
Figure S8. ^{1}H-^{13}C HMBC NMR Spectrum of complex 1 ($CDCl_3$, 298 K) 15
Figure S9. ^{1}H NMR Spectrum of complex 2 ($CDCl_3$(#), 298 K, 400 MHz). 16
Figure S10. ^{13}C NMR Spectrum of complex 2 ($CDCl_3$(#), 298 K, 151 MHz) 16
Figure S11. ^{27}Al NMR spectrum of complex 2 ($CDCl_3$, 298 K, 104 MHz). 17
Figure S12. ^{1}H COSY NMR Spectrum of complex 2 ($CDCl_3$, 298 K, 400 MHz) 17
Figure S13. ^{1}H-^{13}C HSQC NMR Spectrum of complex 2 ($CDCl_3$, 298 K) 18
Figure S14. ^{1}H-^{13}C HMBC NMR Spectrum of complex 2 ($CDCl_3$, 298 K) 18
Figure S15. ^{1}H NMR Spectrum of $LC_{C2Ph2}H_2$ ($CDCl_3$(#), 298 K, 400 MHz) 19
Figure S16. ^{1}H NMR spectrum of complex 3 ($CDCl_3$(#), 298 K, 400 MHz). 19
Figure S17. ^{13}C NMR Spectrum of complex 3 ($CDCl_3$(#), 298 K, 151 MHz) 20
Figure S18. ^{27}Al NMR spectrum of complex 3 ($CDCl_3$, 298 K, 104 MHz). 20
Figure S19. ^{1}H COSY NMR Spectrum of complex 3 ($CDCl_3$, 298 K, 400 MHz) 21
Figure S20. ^{1}H-^{13}C HSQC NMR Spectrum of complex 3 ($CDCl_3$, 298 K) 21
Figure S21. ^{1}H-^{13}C HMBC NMR Spectrum of complex 3 ($CDCl_3$, 298 K) 22
Figure S22. ^{1}H NMR Spectrum of $L_{Phen}H_2$ ($CDCl_3$(#), 298 K, 400 MHz). 22
Figure S23. ^{1}H NMR spectrum of complex 4 (DMSO (#), 298 K, 400 MHz)). 23

Figure S24. ^{13}C NMR Spectrum of complex 4 (DMSO(#), 298 K, 151 MHz) 23
Figure S25. ^{27}Al NMR spectrum of complex 4 (DMSO, 298 K, 104 MHz). 24
Figure S26. ^{1}H COSY NMR Spectrum of complex 4 (DMSO, 298 K, 400 MHz) 24
Figure S27. ^{1}H-^{13}C HSQC NMR Spectrum of complex 4 (DMSO, 298 K) 25
Figure S28. ^{1}H-^{13}C HMBC NMR Spectrum of complex 4 (DMSO, 298 K) 25
Figure S29. ^{1}H NMR Spectrum of $L_{Pro}H_2$ ($CDCl_3$(#), 298 K, 400 MHz) 26
Figure S30. ^{1}H NMR spectrum of complex 5 (CDCl3(#), 298 K, 400 MHz). 26
Figure S31. ^{13}C NMR Spectrum of complex 5 (CD_2Cl_2 (#), 298 K, 151 MHz) 27
Figure S32. ^{27}Al NMR spectrum of complex 5 ($CDCl_3$, 298 K, 104 MHz). 27
Figure S33. ^{1}H COSY NMR Spectrum of complex 5 (CD_2Cl_2, 298 K, 400 MHz) 28
Figure S34. ^{1}H-^{13}C HSQC NMR Spectrum of complex 5 (CD_2Cl_2, 298 K) 28
Figure S35. ^{1}H-^{13}C HMBC NMR Spectrum of complex 5 (CD_2Cl_2, 298 K) 29
Figure S36. ^{1}H NMR Spectrum of $L_{Et}H_2$ (CD_3CN(#), 298 K, 400 MHz). 29
Figure S37. ^{1}H NMR spectrum of complex 6 (MeOD-d^4(#), 298 K, 400 MHz) 30
Figure S38. ^{13}C NMR Spectrum of complex 6 (MeOD-d^4(#), 298 K, 151 MHz) 30
Figure S39. ^{27}Al NMR spectrum of complex 6 (MeOD-d^4, 298 K, 104 MHz). 31
Figure S40. ^{1}H COSY NMR Spectrum of complex 6 (MeOD-d^4, 298 K, 400 MHz) 31
Figure S41. ^{1}H-^{13}C HSQC NMR Spectrum of complex 6 (MeOD-$d^4{}_2$, 298 K) 32
Figure S42. ^{1}H-^{13}C HMBC NMR Spectrum of complex 6 (MeOD-d^4, 298 K) 32
Figure S43. IR spectrum of complex 1 33
Figure S44. MALDI-TOF spectrum of complex 1. 33
Figure S45. IR spectrum of complex 2 34
Figure S46. MALDI-ToF spectrum of complex 2. 34
Figure S47. IR spectrum of complex 3 35
Figure S48. MALDI-ToF spectrum of complex 3. 35
Figure S49. IR spectrum of complex 4 36
Figure S50. MALDI-ToF spectrum of complex 4 36
Figure S51. IR spectrum of complex 5 37
Figure S52. MALDI-ToF spectrum of complex 5. 37
Figure S53. IR spectrum of complex 6 38
Figure S54. MALDI-ToF spectrum of complex 6. 38
Figure S55. Excerpt of ^{1}H NMR spectrum (400 MHz, $CDCl_3$, 298 K) of reactant and products of CHO/PA copolymerization. 40
Figure S56. DOSY ^{1}H NMR spectrum ($CDCl_3$) of polymer formed by PA/CHO ROCOP using catalyst 4.41
Figure S57. VT ^{1}H NMR spectra (400 MHz, CD_2Cl_2) of 1.1. 42
Figure S58. VT ^{1}H NMR spectra (400 MHz, CD_2Cl_2) of 5 42
Figure S59. Graph of [PA] against time for CHO/PA ROCOP catalysed by complex 2. 43
Figure S60. Plot of change in polymer molar mass and dispersity with increasing PA conversion for complex 2. 43
Figure S61. Graph showing the linear correlation between [PA] (mol dm^{-3}) and time (s) for all different [CTA] 44
Figure S62. MALDI-ToF of PCHPE obtained with complex 1. Conditions: [1] : [CHD] : [PA] : [CHO] = 1 : 20 : 400 : 2000 45
Figure S63. MALDI-ToF of PCHPE obtained with complex 1. Conditions: [1] : [CHD] : [PA] : [CHO] = 1 : 100 : 400 : 2000. 45
Figure S64. Graph of TOF against Degree of Polymerisation (DP). 46
Figure S65. A pictorial representation of DP = 10 at two different CTA concentrations 46

Figure S66. DOSY NMR spectrum (500 MHz, MeOD-d^4, 298 K) of complex 1.. 47
Figure S67. TGA of 100 °C isotherm of 1. 48
Figure S68. TGA of 100 °C isotherm of 1 + glycerol ethoxylate (GEO). 49
Figure S69. TGA of 100 °C isotherm of 1.1. 49
Figure S70. TGA of 100 °C isotherm of 1.1 + glycerol ethoxylate (GEO). 50
Figure S71. ^{1}H NMR spectrum (400 MHz, $CDCl_3$, 298 K) of isolated polymer from the polymerisation of CHO/PA. 50
Figure S72. ^{1}H NMR spectrum (400 MHz, $CDCl_3$, 298 K) of isolated polymer from the polymerisation of vCHO/PA. 51
Figure S73. ^{1}H NMR spectrum (400 MHz, $CDCl_3$, 298 K) of PO/PA polymerisation reaction mixture (at 99 % conversion). 51
Figure S74. ^{1}H NMR spectrum (400 MHz, $CDCl_3$, 298 K) of isolated polymer from the polymerisation of AGE/PA. 52
Figure S75. ^{1}H NMR spectrum (400 MHz, $CDCl_3$, 298 K) of isolated polymer from the polymerisation of CHO/NBA. 52
Figure S76. ^{1}H NMR spectrum (400 MHz, $CDCl_3$, 298 K) of isolated polymer from the polymerisation of CHO/TGA. 53
Figure S77. ^{1}H NMR spectrum (400 MHz, $CDCl_3$, 298 K) of isolated polymer from the polymerisation of CHO/DGA. 53
Figure S78. GPC plot of CHO/PA polymerisation at full conversion. 54
Figure S79. GPC plot of vCHO/PA polymerisation at full conversion. 54
Figure S80. GPC plot of PO/PA polymerisation at full conversion. 54
Figure S81. GPC plot of AGE/PA polymerisation at full conversion. 55
Figure S82. GPC plot of CHO/NBA polymerisation at full conversion. 55
Figure S83. GPC plot of CHO/TCA polymerisation at full conversion. 55
Figure S84. GPC plot of CHO/DGA polymerisation at full conversion. 56
Figure S85. Quantitative $^{13}C\{^1H\}$ NMR spectrum (100 MHz, $CDCl_3$, 298 K) of isolated PO/PA polymer to find head-tail regioselectivity. 56

Tables:

Table S1. X-ray crystallography data for $L_{C2Me2}AlK(OAc)_2$ (1) 39
Table S2. ^{1}H Chemical Shifts for the reactant and products of CHO/PA copolymerization in $CDCl_3$. ... 40
Table S3. Rates for CHO/PA ROCOP by complex 4. 41
Table S4. Rates for the ROCOP of CHO/PA using complex 1 with different CHD loadings. 44
Table S5. Rates for the ROCOP of CHO/PA using complex 1.1 with different CHD loadings. 48
Table S6. Rates for the ROCOP of epoxide/PA using complex 1 with different CHD loadings. 57

1. General Procedures and Materials

All manipulations were carried out in a nitrogen filled glovebox, or dual manifold nitrogen-vacuum Schlenk line. Solvents used for synthesis were collected from a solvent purification system (SPS), degassed with freeze-pump-thaw cycles and stored over 3 Å molecular sieves under an inert atmosphere, unless otherwise stated. All materials were stored under a nitrogen atmosphere, in a glovebox. Triethyl aluminium (93%) was purchased from Sigma-Aldrich and was used as received. Group 1 acetate salts were purchased from Sigma Aldrich or Acros Organics and were dried at 100 °C for 16 h before use.

All epoxides (cyclohexene oxide (CHO, Acros Organics), propylene oxide (PO, Sigma Aldrich), vinyl-cyclohexene oxide (vCHO, Sigma Aldrich), allyl glycidyl ether (AGE, Sigma Aldrich)) were purified by stirring over calcium hydride followed by fractional distillation. Purification of phthalic anhydride (PA, Sigma Aldrich) was achieved though stirring in dry toluene. The supernatant was filtered, and the toluene subsequently removed *in vacuo*. The resultant white powder recrystallised from hot (60 °C) chloroform and subsequently sublimed under vacuum at 80 °C. Norbornene anhydride (NBA, Acros Organics) was purified through recrystallisation from dry ethyl acetate, followed by sublimation at 80 °C. Tricyclic anhydride (TCA) was synthesised according to a literature procedure, and was purified through crystallisation from hexane followed by sublimation at 80 °C.[1] Diglycolic anhydride (DGA, Alfa Aesar) was purified by recrystalising twice from acetic anhydride followed by two sublimations.

2. Methods

NMR Spectroscopy: ^{1}H MNR and ^{27}Al spectra were obtained using a Bruker Avance III HD 400 NMR spectrometer. $^{13}C\{H\}$ NMR spectra were obtained with either Bruker AV III HD 500 MHz NMR, Bruker NEO 600 MHz with broadband helium cryoprobe, or Bruker AVIII 700 MHz with inverse TCI $^{1}H/^{13}C/^{15}N$ cryoprobe spectrometers.

IR Spectroscopy: Carried out on a Shimadzu IRSpirit FT-IR spectrometer equipped with a single reflection ATR (attenuated total reflectance) accessory, in a nitrogen filled glovebox.

GPC analysis: Carried out using a Shimadzu LC-20AD instrument, equipped with a Refractive Index (RI) detector and two PSS SDV 5 µm linear M columns. The eluent used was HPLC-grade THF, heated to 30 °C, and with a flow rate of 1.0 mL min^{-1}.

Elemental Analysis: These were performed by the London Metropolitan University Elemental Analysis Service, 29 Hornsey Road, London, N7 7DD, UK

MALDI-ToF: Carried out on a Bruker Autoflex Speed MALDI-ToF spectrometer. Polymer samples were prepared by a 1:4:1 solution of polymer (10 mg mL^{-1} in THF), dithranol (10 mg mL^{-1} in THF) and KTFA (10 mg mL^{-1} in THF). Catalyst samples were prepared by a 1:4:1 solution of catalyst (10 mg mL^{-1} in $CHCl_3$ (MeCN for **4**, MeOH for **6**)), trans-2-[3-(4-tert-Butylphenyl)-2-methyl-2-propenylidene]malononitrile (10 mg mL^{-1} in THF) and KTFA or KI (10 mg mL^{-1} in THF). This solution was spotted three times on a MALDI plate and allowed to dry completely before analysis was undertaken.

X-Ray Crystallography: Crystallographic data were collected, and structures solved, by Francesca Fiorentini. Air sensitive crystalline samples were isolated in a nitrogen filled glovebox and immersed in fluorinated oil. Crystalline samples were mounted on a MiTeGen Micromount, and cooled to 150 K with dry nitrogen using an Oxford Cryosystem.[2] Data was collected using an Oxford Diffraction Supernova diffractometer using Cu K_α (λ = 1.5417 Å) or Mo K_α (λ = 0.7107 Å) radiation. The resulting reflection data was processed with CrysAlis Pro.[3] The crystal structures were solved using the SHELXT program and least-square refined using the SHELXL program within the Olex2 system suite.[4]

Thermal gravimetric analysis (TGA): were collected on a TGA5500 System (TA Instruments), equipped with the TRIOS software package.

3. Polymerization methods

3.1 Epoxide/Anhydride polymerisation

The reaction was set up inside a nitrogen-filled glovebox. The catalyst and monomers were added with their desired weights to a 4mL vial, with a rare-earth magnetic stirrer bar inside. A melamine cap containing a Teflon inlay, was used to seal the vial, with an additional layer of parafilm and then electrical insulation tape wrapped around the cap. The reaction mixture was heated at 100 °C, with stirring at 1400 rpm. At the times recorded, the reaction mixture was removed from heating and immediately cooled in an ice bath to slow the reaction rate such that it was effectively paused. Aliquots were taken under inert atmosphere in a glovebox, but the ^{1}H NMR spectroscopy (2mg crude product in 0.6mL $CDCl_3$) and GPC (2mg crude product in 1mL THF and filtered) samples could be prepared in air.

3.2 Polymer isolation

DCM (1 mL) was added to the polymerisation reaction mixture to dissolve the polymer. This was then added dropwise to 200 mL MeOH, with high stirring (creating a vortex), forming a white precipitate that could be isolated by reduced pressure filtration.

4. Synthesis of complexes

4.1 Synthesis of ligand (LH_2)

The synthesis of the *ortho*-vanillin and diamine ligand followed general literature procedure. [5] [6] Formation of a Schiff base LH_2 was used to produce the ligand by addition of the desired diamine and 2 equiv *ortho*-vanillin in MeOH, at room temperature and pressure. ^{1}H NMRs of LH_2 were in agreement with previously reported literature. [6] [7] [8] Crude yields: $L_{C2Me2}H_2$ = 50 %, $L_{Cyc}H_2$ = 82 %, $L_{C2Ph2}H_2$ = 98 %, $L_{Pro}H_2$ = 78 % & $L_{Et}H_2$ = 79 %.

Synthesis of $L_{Phen}H_2$ was achieved by addition of *o*-phenylene diamine to *ortho*-vanillin in EtOH with reflux at 80 °C for 4h.[9]

4.2 Synthesis of complex **1**, [$L_{C2Me2}AlK(OAc)_2$]

The synthesis of complex **1** was achieved through a two-step procedure, following previous literature work.[10] $AlEt_3$ (0.12 g, 0.9 mmol) was added to a solution of $L_{C2Me2}H_2$ (0.32 g, 0.9 mmol), in toluene (10

mL), under N_2 atmosphere, with stirring for 16 h, yielding [L_{C2Me2}AlEt] as a yellow precipitate (crude yield = 50 %). Acetic acid (0.02 g, 0.3 mmol) was added, under N_2 flow, to a stirring mixture of [L_{C2Me2}AlEt] (0.12 g, 0.3 mmol), in toluene (10 mL) and heated at 100 °C, for 12 h. [L_{C2Me2}AlOAc] was isolated as a light yellow precipitate (crude yield = 75 %) and was washed 2x with Toluene and 1x with hexane. KOAc (13 mg, 0.13 mmol) was then added to [L_{C2Me2}AlOAc] (57 mg, 0.13 mmol), in chloroform (10 mL), with stirring under N_2 atmosphere for 16 h at room temperature. The solvent was removed under vacuum leaving [L_{C2Me2}AlK(OAc)$_2$] (**1**), a pale yellow powder. **^{1}H NMR** ($CDCl_3$, 400 MHz, 298 K): 8.16 (s, 1H, **H**C=N), 8.08 (s, 1H, **H**C=N), 6.81 (d, ^3J = 8.00 Hz, 2H, *o/p*-Ph**H**), 6.69 (d, ^3J = 8.00 Hz, 2H, *o/p*-Ph**H**), 6.57 (br. m, 2H, *m*-Ph**H**), 3.88 (br. s, 2H, **H**C-N), 3.37 (br. 2s, 6H, , -OMe**H**), 1.60 (s, 6H, -C((C**H**$_3$*)$_2$/OAc**H**), 1.56 (s, 6H, -C((C**H**$_3$*)$_2$/OAc**H**). **^{13}C NMR** (151 MHz, $CDCl_3$, 298 K): δ 174.19, 156.13, 155.29, 151.72, 151.50, 125.59, 124.85, 119.07, 118.95, 114.62, 114.11, 113.94, 66.27, 60.48, 55.00, 27.88, 24.66. **IR:** 349, 353, 357, 360, 372, 380, 453, 465, 490, 505, 538, 568, 612, 632, 658, 731, 779, 806, 859, 935, 969, 985, 1011, 1075, 1099, 1165, 1185, 1218, 1234, 1310, 1371, 1387, 1444, 1467, 1475, 1545, 1595, 1608, 1627, 1650, 2819, 2911, 2948, 3042 cm^{-1}. **MALDI-ToF:** 479 m/z [L_{C2Me2}AlK(OAc)]$^+$

4.3 Synthesis of complex **2**, [L_{Cyc}AlK(OAc)$_2$]

This followed an analogous procedure to that outlined above for complex **1**. Firstly, the monometallic [L_{Cyc}AlOAc] was targeted. $AlEt_3$ (0.16 g, 1.4 mmol) was added to a solution of $L_{Cyc}H_2$ (0.50 g, 1.3 mmol), in toluene (10 mL), under N_2 atmosphere, with stirring and heated for 2 h at 100 °C, producing [L_{Cyc}AlEt] *in situ* as a light brown solution. Acetic acid (0.08 g, 1.3 mmol) was added, under N_2 flow, and stirring, with the reaction mixture heated for a further 1.5 h at 100 °C and before being left stirring at room temperature for 2 days. [L_{Cyc}AlOAc] was isolated as a yellow precipitate (crude yield = 77 %) and was washed 2x with Toluene and 1x with hexane. KOAc (13 mg, 0.13 mmol) was then added to [L_{Cyc}AlOAc] (60 mg, 0.13 mmol), in chloroform (10 mL), with stirring under N_2 atmosphere for 24 h at room temperature. The solvent was removed under vacuum leaving [L_{Cyc}AlK(OAc)$_2$] (**2**), a cream/yellow powder (72 mg, 0.13 mmol, 99 %). **^{1}H NMR** ($CDCl_3$, 400 MHz, 298 K): 7.99 (s, 2H, **H**C=N), 6.80 (dd, ^3J = 4.00 Hz ^3J = 4.00 Hz, 2H, *m*-Ph**H**), 6.56 (m, 4H, *o*/*p*-Ph**H**), 3.97 (s, 2H, **H**C-N), 3.11 (s, 6H, -OMe**H**), 2.51 (s (d?), 2H, -C**H**$_2$*), 2.06 (s, 2H, -C**H**$_2$*), 1.63 (s, 6H, OAc**H**), 1.50 (s, 4H, -C**H$_2$**). **^{13}C NMR** (151 MHz, $CDCl_3$, 298 K): δ 174.21, 160.08, 154.96, 151.27, 125.45, 118.92, 114.26, 113.39, 63.22, 54.45, 27.11, 24.68, 24.15. **IR:** 349, 356, 360, 367, 386, 392, 455, 598, 612, 658, 731, 779, 808, 859, 969, 1011, 1076, 1099, 1164, 1185, 1218, 1234, 1312, 1371, 1387, 1448, 1468, 1477, 1545, 1608, 1627, 1644, 2821, 2915, 2949, 3042 cm^{-1}. **MALDI-ToF:** 505 m/z [L_{Cyc}AlK(OAc)]$^+$

4.4 Synthesis of complex **3**, [L_{C2Ph2}AlK(OAc)$_2$]

This followed an analogous procedure to that outlined above for complex **1**. $AlEt_3$ (0.13 g, 1.1 mmol) was added to a solution of $L_{C2Ph2}H_2$ (0.50 g, 1.0 mmol), in toluene (10 mL), under N_2 atmosphere, with stirring for 24 h. [L_{C2Ph2}AlEt] was isolated from solution by removal of solvent under vacuum, giving a yellow powder (crude yield = 98 %). Acetic acid (0.04 g, 0.6 mmol) was added, under N_2 flow, to a stirring mixture of [L_{C2Ph2}AlEt] (0.32 g, 0.6 mmol), in toluene (10 mL). This was left for 24 h at room temperature before being heated at 65 °C for 3 h. [L_{C2Ph2}AlOAc] was isolated as a yellow gel that had precipitated out of solution. This was then washed 2x with toluene and 1x with hexane before being dried, yielding a yellow powder (crude yield = 36 %). KOAc (21 mg, 0.22 mmol) was then added to [L_{C2Ph2}AlOAc] (121 mg, 0.22 mmol), in chloroform (10 mL), with stirring under N_2 atmosphere for 24 hat room temperature. The solvent was removed under vacuum leaving [L_{C2Ph2}AlK(OAc)$_2$] (**3**), a yellow powder (151 mg, 0.23 mmol, crude yield = 99 %). **^{1}H NMR** ($CDCl_3$, 400 MHz, 298 K): 7.61 (s, 2H, **H**C=N), 7.28 (s, 10H, -Ph**H**), 6.55 (d, ^3J = 8.00 Hz, 4H, *o/p*-Ph**H**), 6.48 (t/dd, ^3J = 6.00 Hz, 2H, *m*-Ph**H**), 5.61 (s, 2H, **H**C-N), 3.12 (s, 6H, -OMe**H**), 1.84 (s, 6H, OAc**H**). **^{13}C NMR** (151 MHz, $CDCl_3$, 298 K): δ 174.12, 164.08, 155.24, 151.31, 136.83, 130.34, 129.10, 128.60, 125.83, 118.90, 114.40, 113.76, 72.31, 54.59, 25.25. **IR:** 347, 355, 359, 372, 397,465, 523, 545, 598, 609, 643, 698, 731, 779, 806, 859, 925, 981, 1001, 1038, 1075, 1101, 1164, 1188, 1215, 1235, 1312, 1378, 1444, 1467, 1545, 1593, 1615, 1644, 1675, 1691, 1708, 1724, 2819, 2914, 2945, 2986, 3011, 3042 cm^{-1}. **MALDI-ToF:** 603 m/z [L_{C2Ph2}AlK(OAc)]$^+$

4.5 Synthesis of complex **4**, [L_{Phen}AlK(OAc)$_2$]

This followed an analogous procedure to that outlined above for complex **1**. $AlEt_3$ (0.13 g, 1.3 mmol) was added to a solution of $L_{Phen}H_2$ (0.50 g, 1.3 mmol) in THF (10 mL), under N_2 atmosphere, with stirring for 2 h, yielding [L_{Phen}AlEt] as a precipitate (crude yield = 68 %). Acetic acid (0.04 g, 0.6 mmol) was added, under N_2 flow, to a stirring mixture of [L_{Phen}AlEt] (0.27 g, 0.6 mmol) in THF (10 mL). This was left, for 20 h, at room temperature before heating at 65 °C, for 1.5 h. [L_{Phen}AlOAc] was then washed 2x with toluene and 1x with hexane before being dried, yielding a yellow precipitate (crude yield = 25 %). KOAc (13 mg, 0.13 mmol) was then added to [L_{Phen}AlOAc] (60 mg, 0.13 mmol), in acetonitrile (10 mL), with stirring and under N_2 atmosphere. This was left stirring at room temperature, for 3 day, before the solvent was removed under vacuum leaving [L_{Phen}AlK(OAc)$_2$] (**4**), a yellow powder (53 mg, 0.09 mmol, crude yield = 73 %). **^{1}H NMR** (DMSO, 400 MHz, 298 K): 9.01 (s, 2H, **H**C=N), 8.05 (m, 2H, *o/p*-Ph**H**), 7.45 (br. s, 2H, *o/p*-Ph**H**), 7.15 (d, ^3J = 8.00 Hz, 2H, Ph**H**), 7.02 (d, ^3J = 8.00 Hz, 2H, Ph**H**), 6.63

(br. s, 2H, *m*-Ph**H**), 3.84 (s, 6H, -OMe**H**), 1.29 (s, 6H, OAc**H**). **^{13}C NMR** (151 MHz, DMSO, 298 K): δ 171.36, 158.51, 156.13, 150.74, 138.29, 127.69, 126.44, 118.79, 116.47, 114.51, 113.84, 55.18, 24.54. **IR:** 349, 360, 369, 383, 403, 415, 440, 459, 495, 515, 545, 578, 586, 598, 613, 632, 649, 735, 741, 763, 781, 806, 842, 859, 896, 911, 934, 972, 982, 1008, 1041, 1075, 1101, 1164, 1174, 1189, 1234, 1315, 1324, 1368, 1440, 1474, 1498, 1541, 1573, 1607, 1624, 1675, 1691, 1708, 1724, 2825, 2919, 2951, 3044 cm^{-1}. **Anal. Calc.** $C_{26}H_{24}AlKN_2O_8$: C, 55.90; H, 4.33; N, 5.02 %. Found: C, 55.58; H, 4.04; N, 5.00 %. **MALDI-ToF:** 499 m/z [L_{Phen}AlK(OAc)]$^+$

4.6 Synthesis of complex **5**, [L_{Pro}AlK(OAc)$_2$]

This followed an analogous procedure to that outlined above for complex **1**. Firstly, the monometallic [L_{Pro}AlOAc] was targeted. 1.05 equiv. of $AlEt_3$ (188 mg, 1.53 mmol) was added to a solution of $L_{Pro}H_2$ (500 mg, 1.46 mmol), in THF (10 mL), under N_2 atmosphere with stirring, for 48 h, producing [L_{Pro}AlEt] as an orange solution. The solvent was removed under vacuum and was isolated as an orange-yellow solid (crude yield = 99 %) and used without further purification. Acetic acid (38 mg, 0.63 mmol) was added, under N_2 flow, to a stirring solution of [L_{Pro}AlEt] (250 mg, 0.63 mmol), in THF (10 mL), with the reaction mixture heated to 100 °C for 3.5 h before being left stirring at room temperature for 3 days. [L_{Pro}AlOAc] was isolated from the yellow solution by removing the solvent under vacuum, giving a yellow powder (crude yield = 81 %) and used without further purification. KOAc (20 mg, 0.21 mmol) was then added to [L_{Pro}AlOAc] (88 mg, 0.21 mmol), in chloroform (10 mL), with stirring and under N_2 atmosphere. This was left at room temperature, for 48 h, before the solvent was removed under vacuum leaving [L_{Pro}AlK(OAc)$_2$] (**5**), a pale yellow powder (96 mg, 0.18 mmol, 88 %). **^{1}H NMR** ($CDCl_3$, 400 MHz, 298 K): 7.96 (s, 2H, **H**C=N), 6.75 (dd, ^3J = 8.00 Hz, ^4J = 4.00 Hz, 2H, Ph**H**), 6.60 (dd, ^3J = 8.00 Hz, ^4J = 4.00 Hz, 2H, Ph**H**), 6.50 (t/dd, ^3J = 8.00 Hz, 2H, Ph**H**), 3.76 (t, ^3J = 6.00 Hz, 4H, **H**C-N), 3.35 (s, 6H, -OC**H**$_3$), 2.26 (br. s, 2H, -C**H**$_2$), 1.56 (s, 6H, -OC(=O)C**H**$_3$). **^{13}C NMR** (151 MHz, CD_2Cl_2, 298 K): δ 173.62, 166.08, 156.42, 151.50, 125.38, 119.90, 114.46, 113.97, 63.07, 55.34, 29.15, 24.74. **IR:** 343, 349, 372, 390, 396, 417, 445, 492, 532, 568, 609, 631, 649, 729, 781, 805, 858, 936, 952, 968, 985, 1011, 1038, 1069, 1098, 1164, 1219, 1235, 1312, 1380, 1448, 1467, 1480, 1550, 1608, 1675, 1691, 1708, 1724, 282, 2908, 2942, 2982, 3042 cm^{-1}. **Anal. Calc.** $C_{23}H_{26}AlKN_2O_8$: C, 52.66; H, 5.00; N, 5.34 %. Found: C, 52.53; H, 5.22; N, 5.48 %. **MALDI-ToF:** 465 m/z [L_{Pro}AlK(OAc)]$^+$

4.7 Synthesis of complex **6**, [L_{Et}AlK(OAc)$_2$]

This followed an analogous procedure to that outlined above for complex **1**. $AlEt_3$ (0.20 g, 1.6 mmol) was added to a solution of $L_{Et}H_2$ (0.50 g, 1.5 mmol) in toluene (10 mL), under N_2 atmosphere, with

stirring at room temperature for 24 h, yielding [L_{Et}AlEt] as a yellow precipitate (crude yield = 29 %). Acetic acid (0.01 g, 0.2 mmol) was added, under N_2 flow, to a stirring mixture of [L_{Et}AlEt] (0.08 g, 0.2 mmol) in toluene (10 mL). This was heated to 100 °C for 1.5 h, before being left stirring at room temperature for 48 h. [L_{Et}AlOAc] was then washed 2x with toluene and 1x with hexane before being dried, yielding a yellow precipitate (yield = 98 %). KOAc (15 mg, 0.16 mmol) was then added to [L_{Et}AlOAc] (65 mg, 0.16 mmol), in chloroform (10 mL), with stirring and under N_2 atmosphere. This was left stirring at room temperature for 20 h, before the solvent was removed under vacuum leaving [L_{Et}AlK(OAc)$_2$] (**6**), a yellow powder that was then purified by 3x toluene and 2x hexane washes (75 mg, 0.15 mmol, crude yield = 93 %). . **^{1}H NMR** ($CDCl_3$, 400 MHz, 298 K): 8.47 (s, 2H, **H**C=N), 7.09 (dd, ^3J = 8.00 Hz , ^4J = 4.00 Hz, 2H, *o/p*-Ph**H**), 7.01 ((dd, ^3J = 8.00 Hz , ^4J = 4.00 Hz, 2H, *o/p*-Ph**H**), 6.74 (t , ^3J = 8.00 Hz, 2H, *m*-Ph**H**), 4.03 (s, 2H, **H**C-N), 3.91 (s, 6H, -OMe**H**), 1.79 (s, 6H, -OAc**H**). **^{13}C NMR** (151 MHz, $CDCl_3$, 298 K): 178.34, 169.06, 156.00, 152.36, 126.61, 120.42, 116.79, 116.08, 55.89, 54.56, 23.67. **IR:** 350, 355, 360, 366, 373, 380, 386, 390, 398, 405, 416, 473, 496, 515, 539, 576, 600, 613, 625, 639, 733, 746, 783, 861, 911, 939, 962, 982, 1014, 1042, 1076, 1101, 1159, 1215, 1232, 1311, 1325, 1384, 1445, 1467, 1545, 1593, 1607, 1633, 1650, 2818, 2914, 2981, 3042 cm^{-1}. **MALDI-ToF:** 451 m/z [L_{Et}AlK(OAc)]$^+$

5. Supplementary Figures and Tables

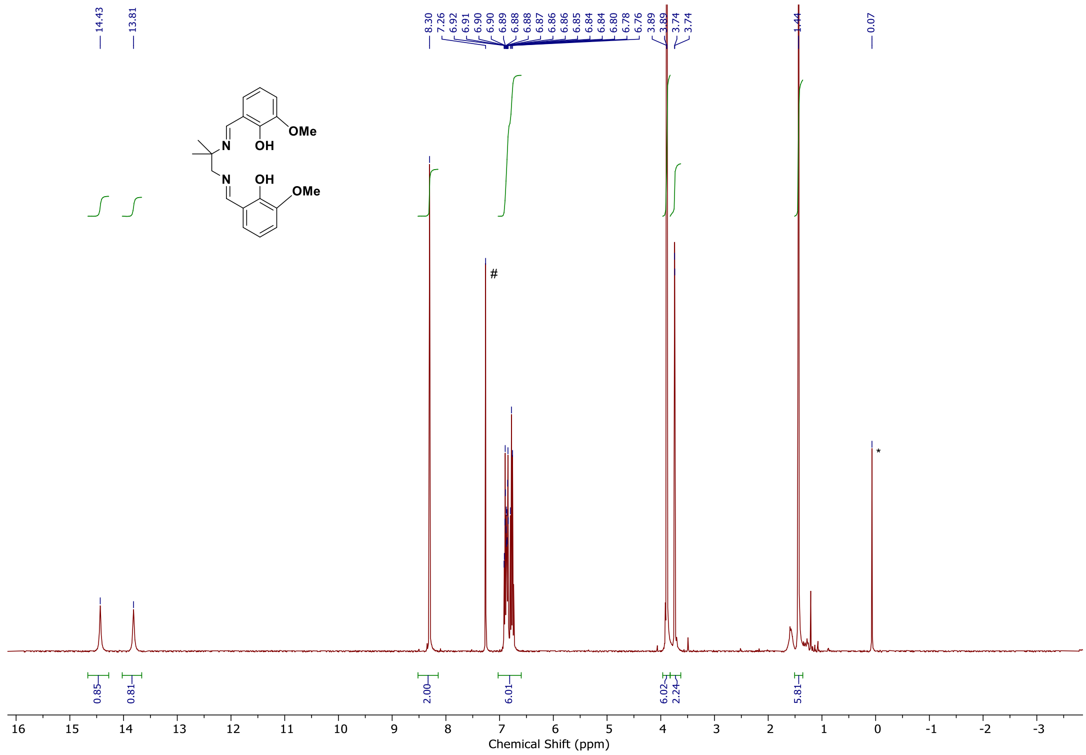

Figure S1. ^{1}H NMR Spectrum of $L_{C2Me2}H_2$ ($CDCl_3$(#), 298 K, 400 MHz) *: impurity from grease.

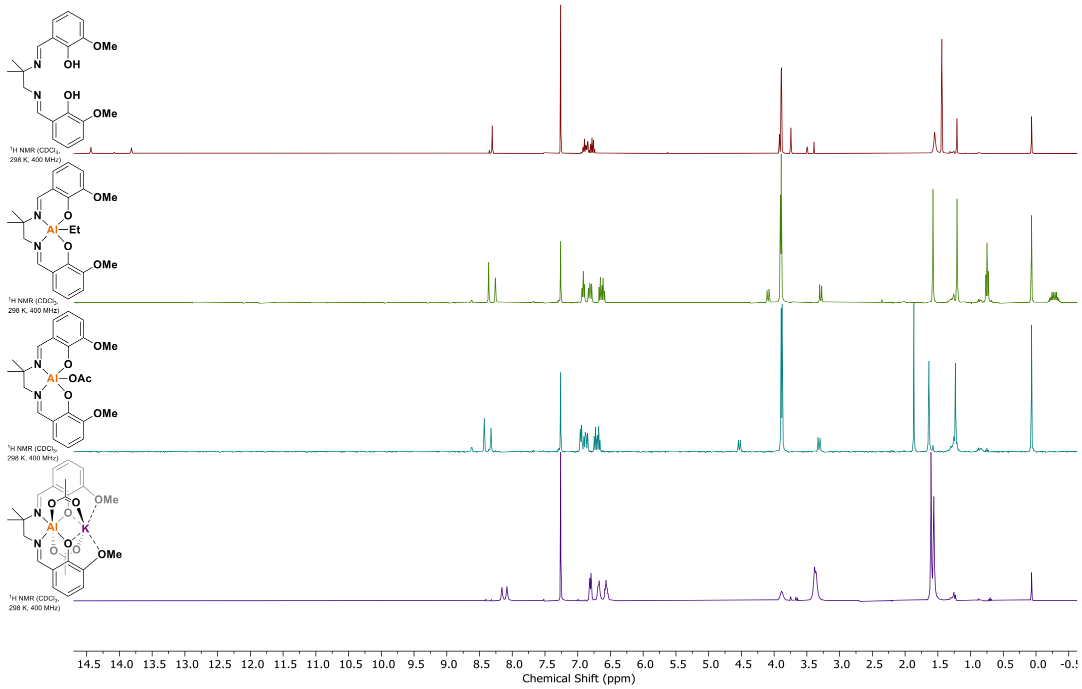

Figure S2 ^{1}H NMR Spectra of synthesis route for complex **1**.

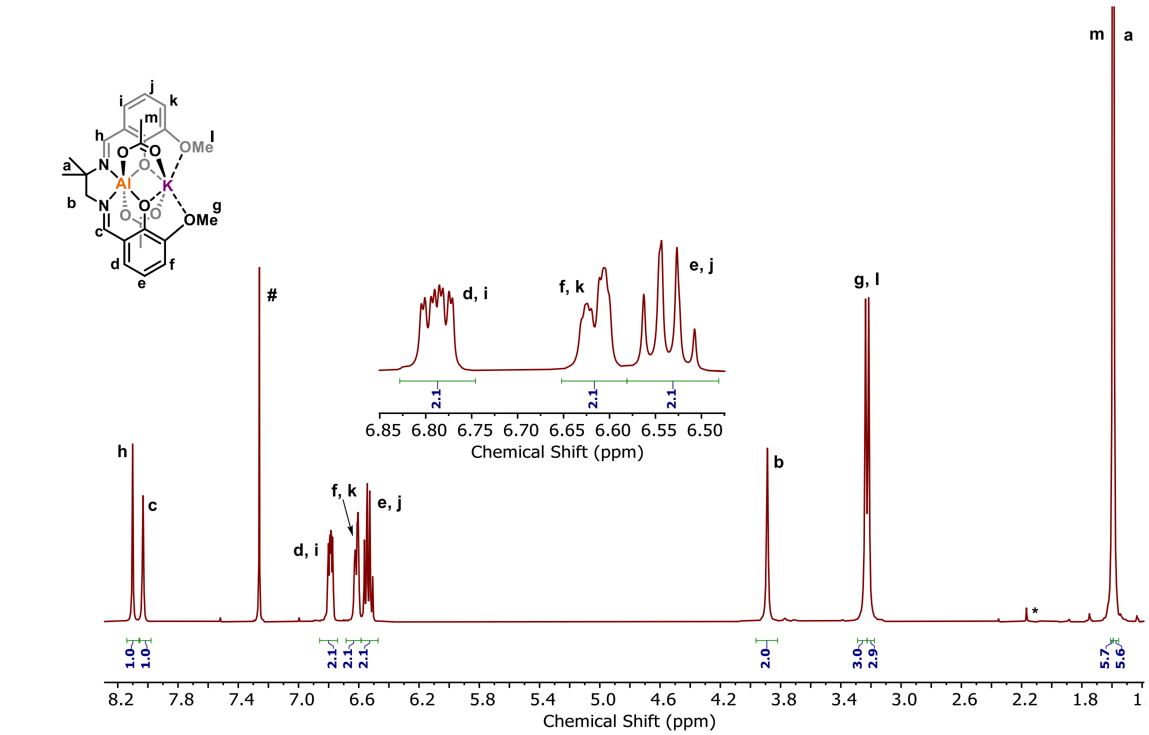

Figure S3. ^{1}H NMR Spectrum of complex **1** ($CDCl_3$(#), 298 K, 400 MHz) *: impurity from acetone.

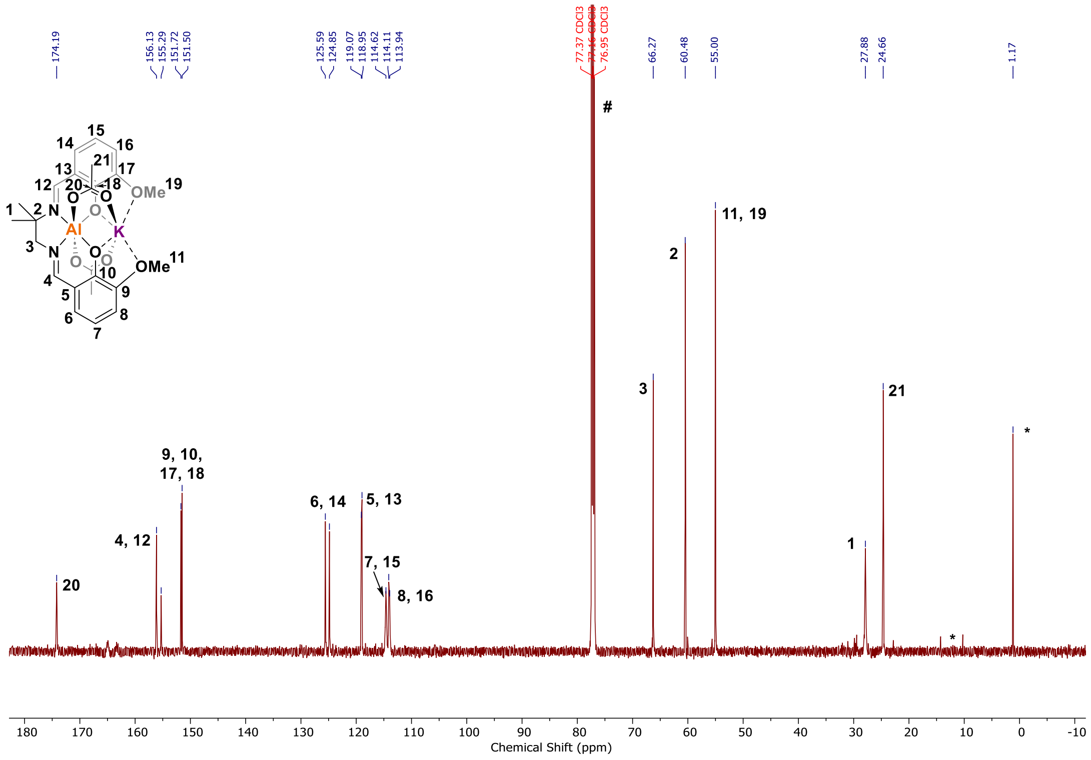

Figure S4. ^{13}C NMR Spectrum of complex **1** ($CDCl_3$(#), 298 K, 151 MHz) *: impurities from grease and hexane (in glovebox atmosphere).

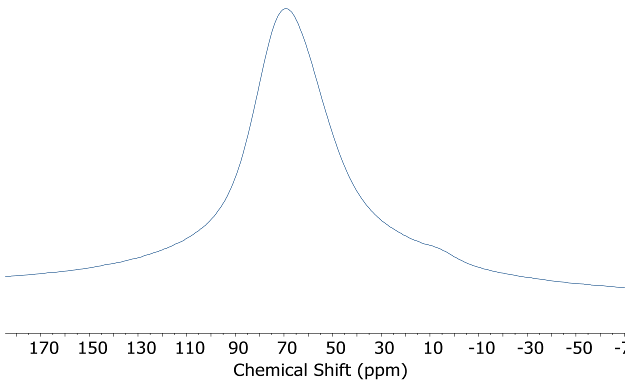

Figure S5. ^{27}Al NMR Spectrum of complex **1** ($CDCl_3$, 298 K, 104 MHz).

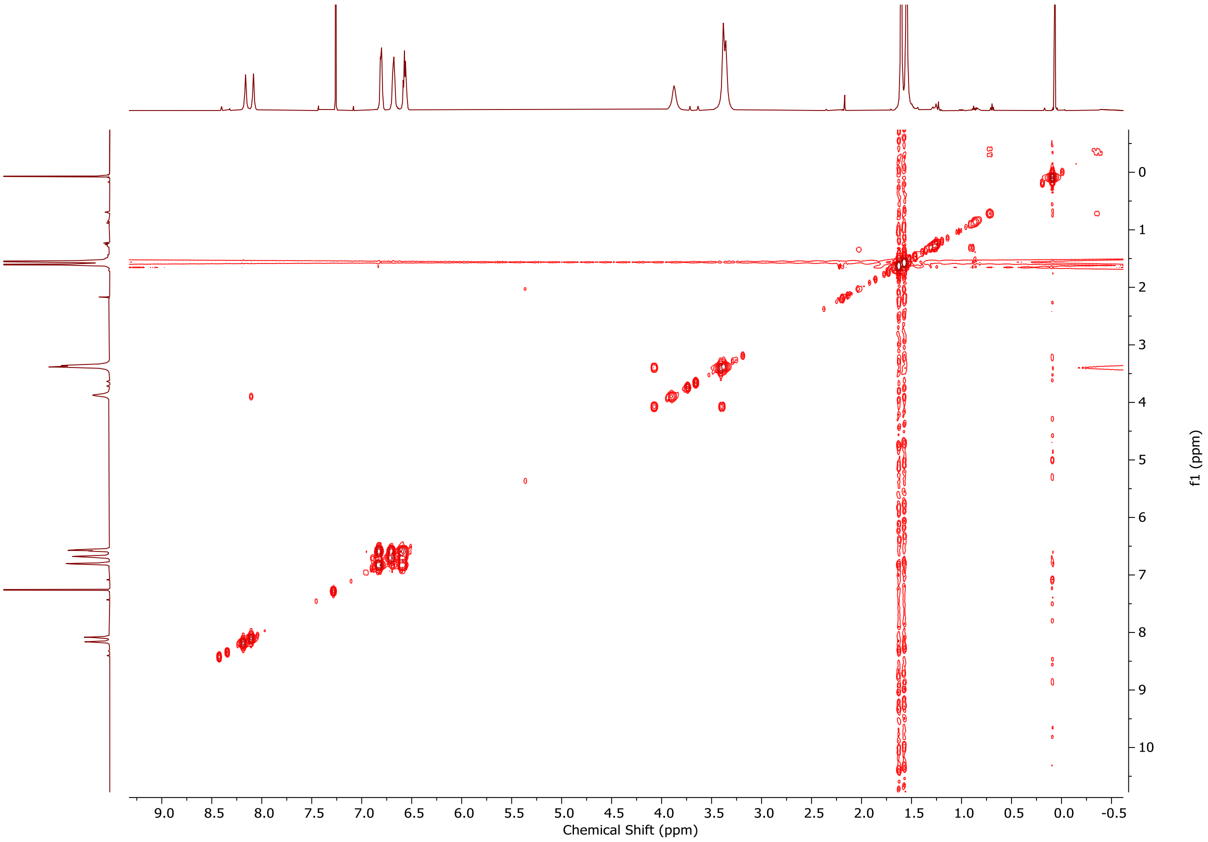

Figure S6. ^{1}H COSY NMR Spectrum of complex **1** ($CDCl_3$, 298 K, 400 MHz)

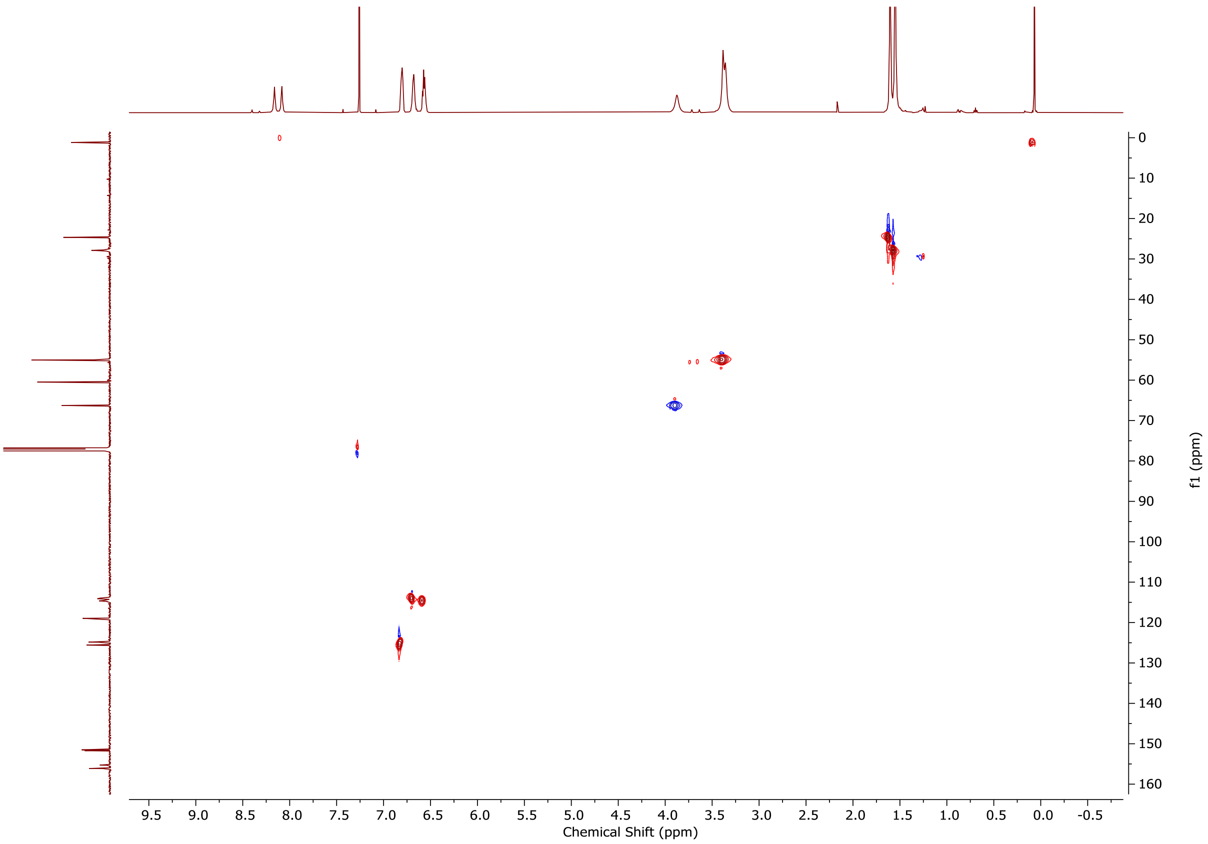

Figure S7. ^{1}H-^{13}C HSQC NMR Spectrum of complex **1** ($CDCl_3$, 298 K)

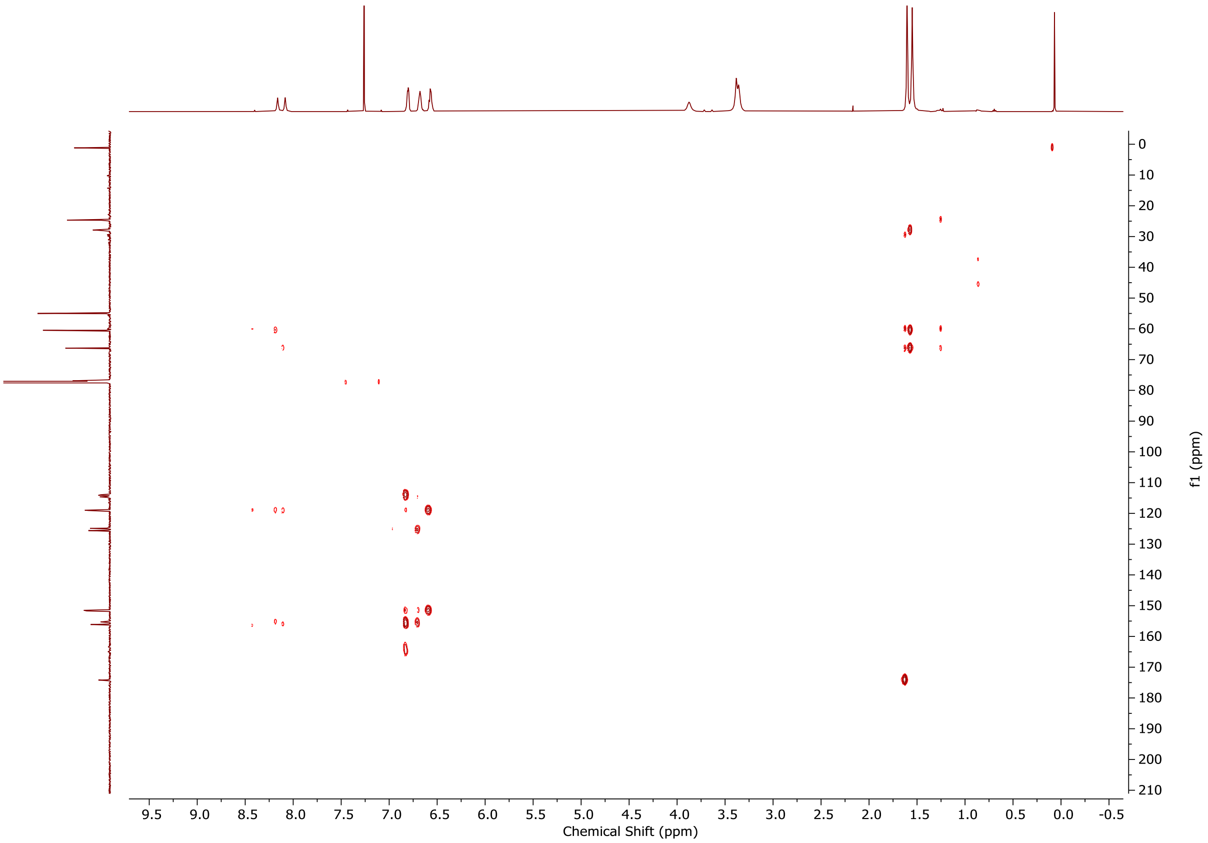

Figure S8. ^{1}H-^{13}C HMBC NMR Spectrum of complex **1** ($CDCl_3$, 298 K)

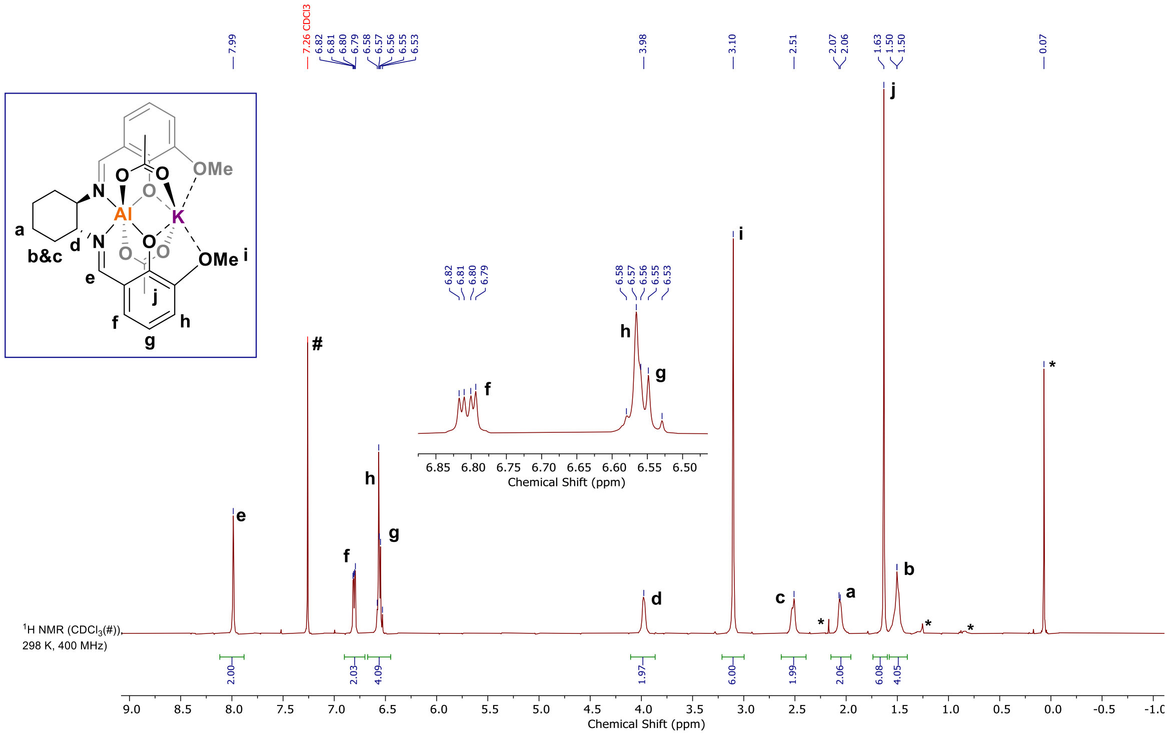

Figure S9. ^{1}H NMR Spectrum of complex **2** ($CDCl_3$(#), 298 K, 400 MHz) *: impurities from grease and hexane (in glovebox atmosphere).

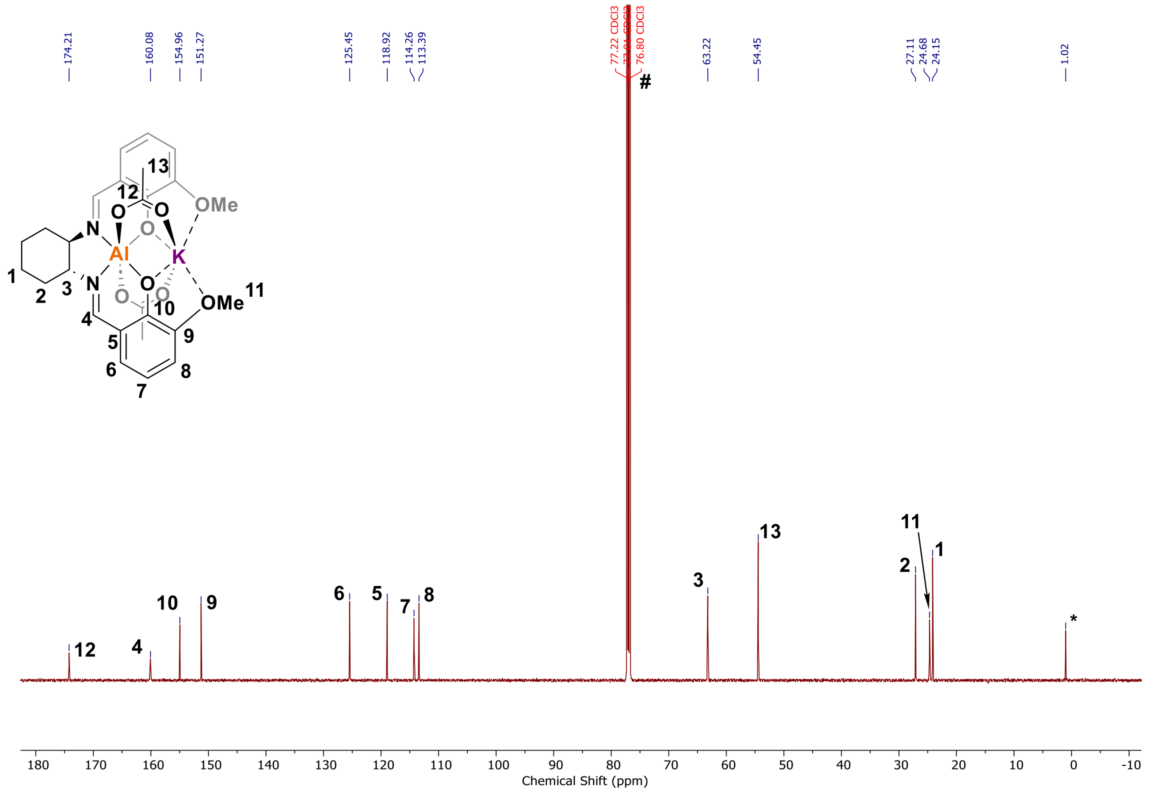

Figure S10. ^{13}C NMR Spectrum of complex **2** ($CDCl_3$(#), 298 K, 151 MHz) *: impurity from grease.

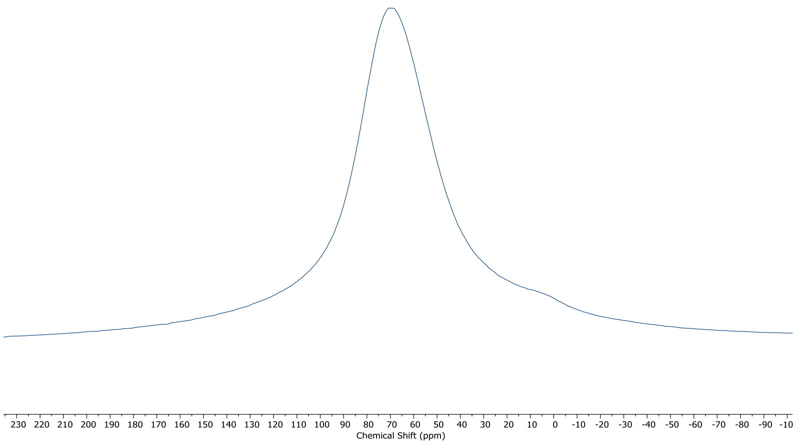

Figure S11. ^{27}Al NMR spectrum of complex **2** ($CDCl_3$, 298 K, 104 MHz).

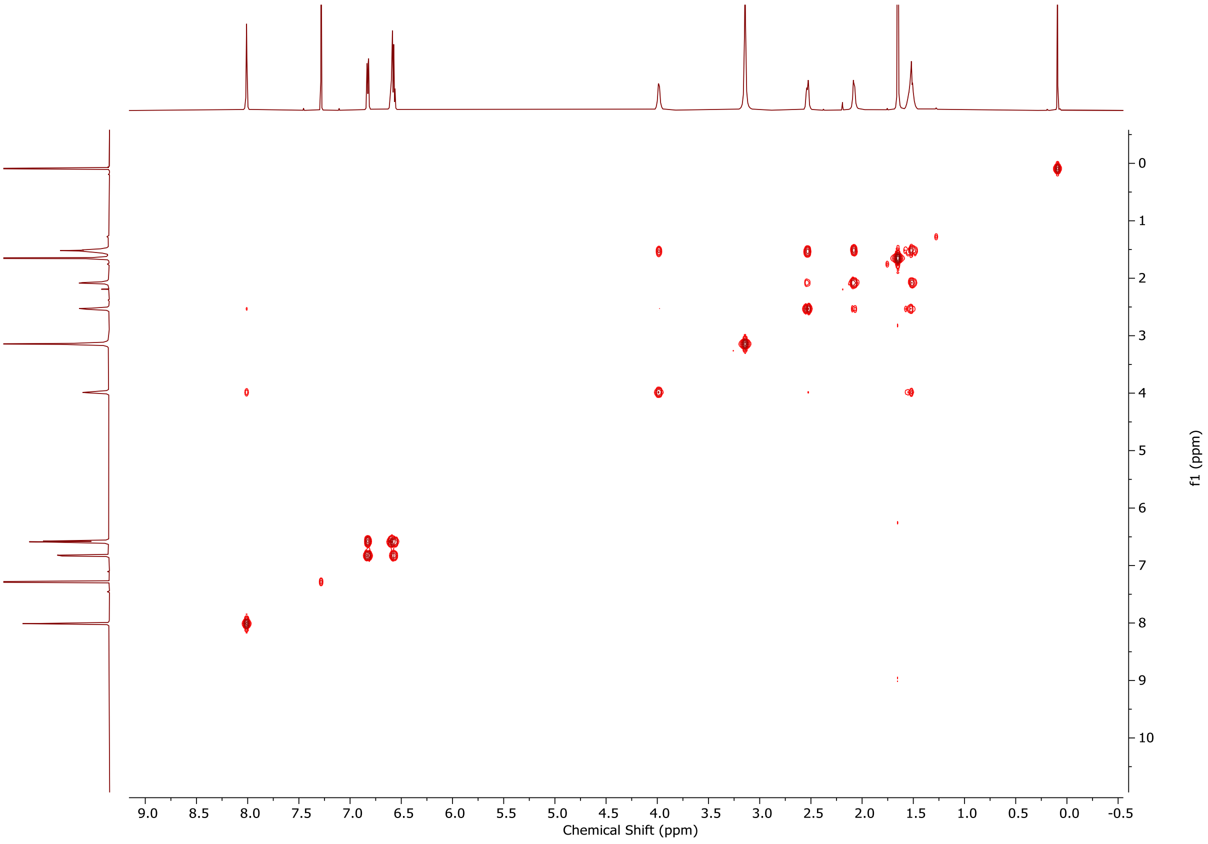

Figure S12. ^{1}H COSY NMR Spectrum of complex **2** ($CDCl_3$, 298 K, 400 MHz)

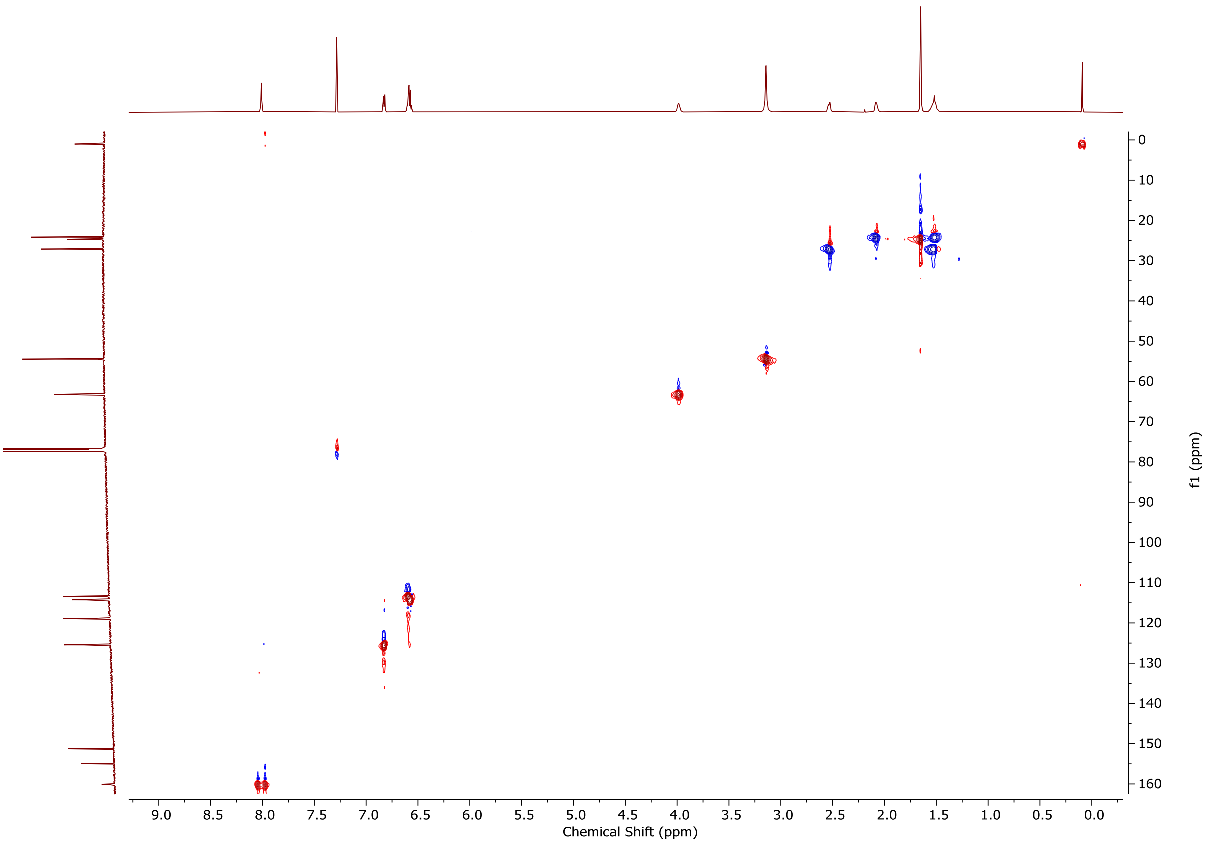

Figure S13. ^{1}H-^{13}C HSQC NMR Spectrum of complex **2** ($CDCl_3$, 298 K).

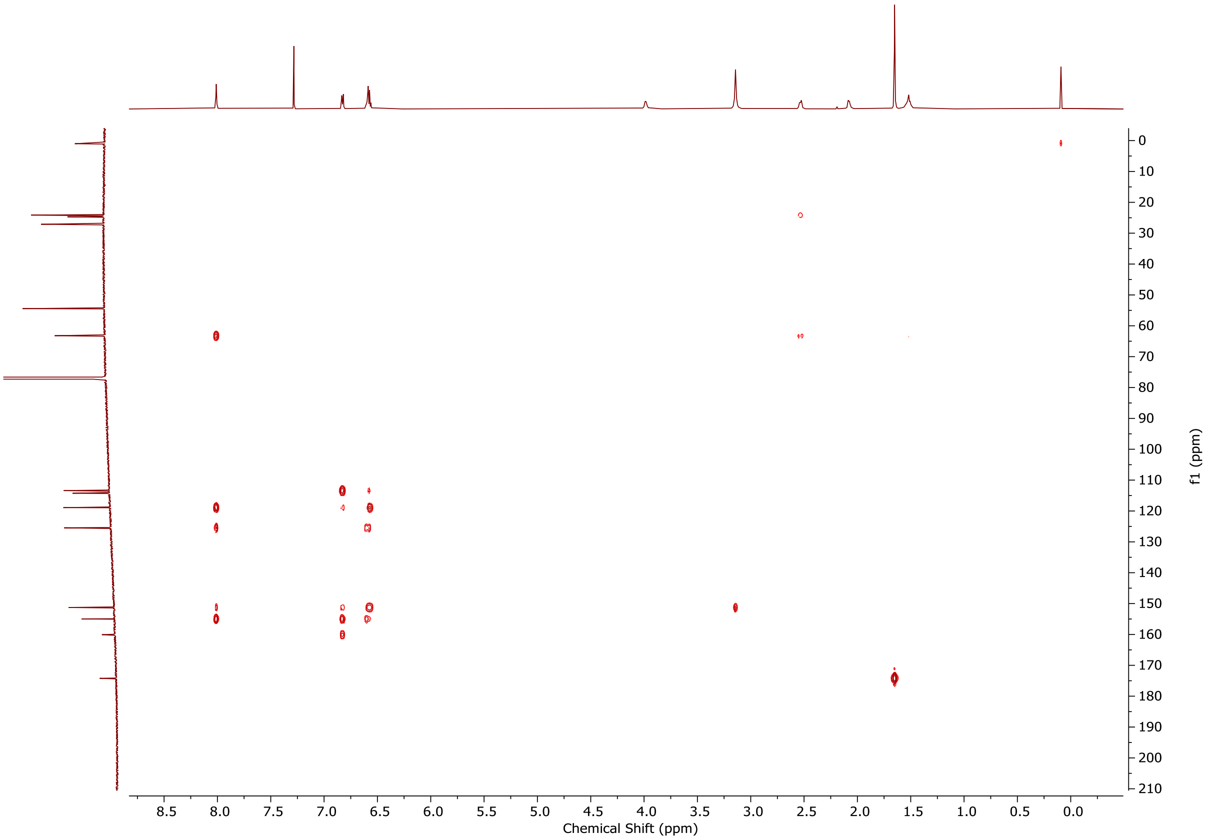

Figure S14. ^{1}H-^{13}C HMBC NMR Spectrum of complex **2** ($CDCl_3$, 298 K)

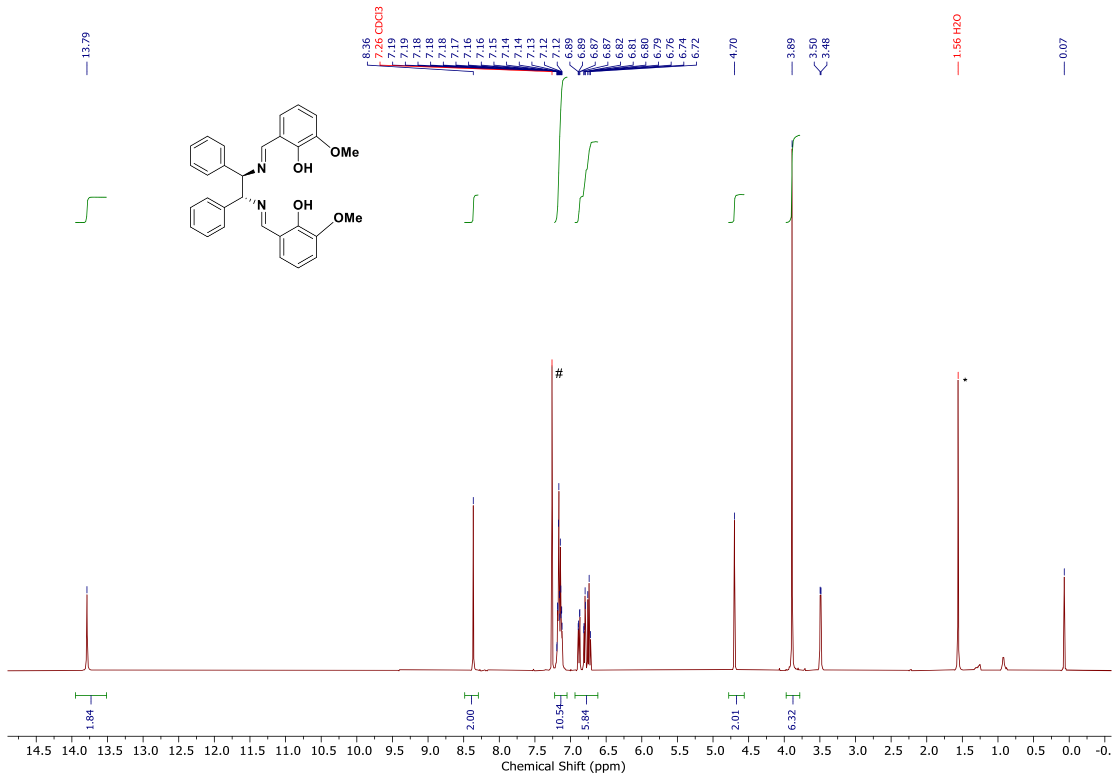

Figure S15. ^{1}H NMR Spectrum of $LC_{C2Ph2}H_2$ ($CDCl_3$(#), 298 K, 400 MHz) *: impurities from water and grease.

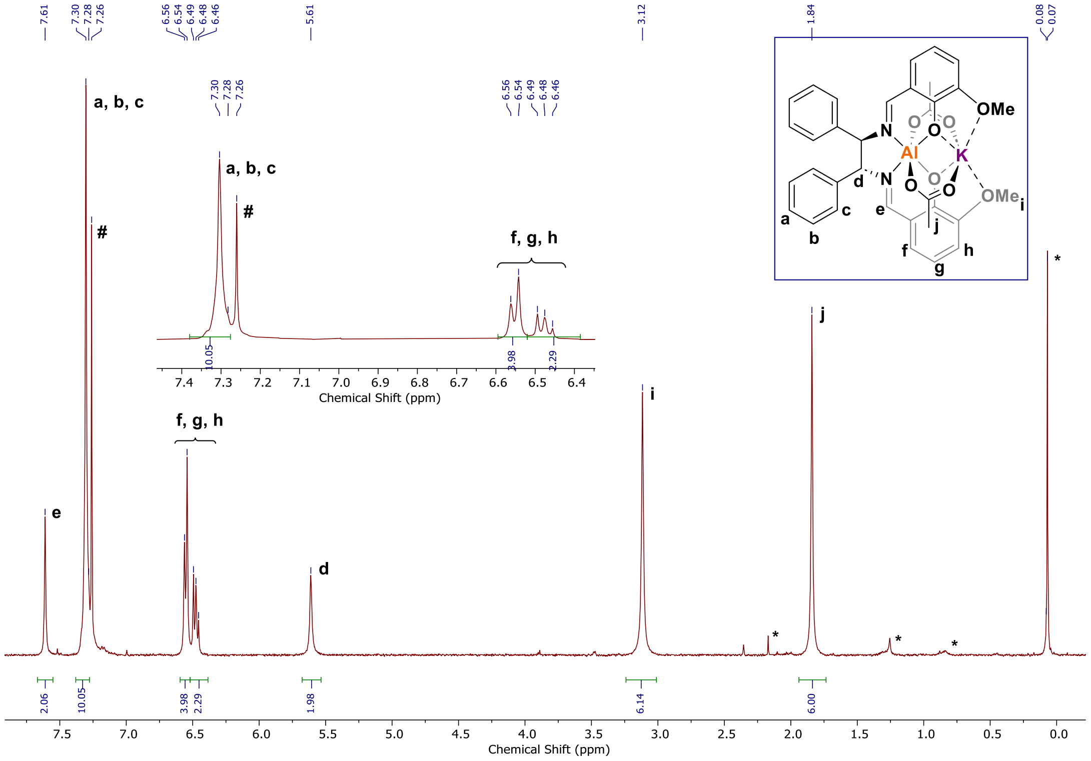

Figure S16. ^{1}H NMR spectrum of complex **3** ($CDCl_3$(#), 298 K, 400 MHz) *: impurities from grease, hexane (glovebox atmosphere) and acetone (from NMR tube).

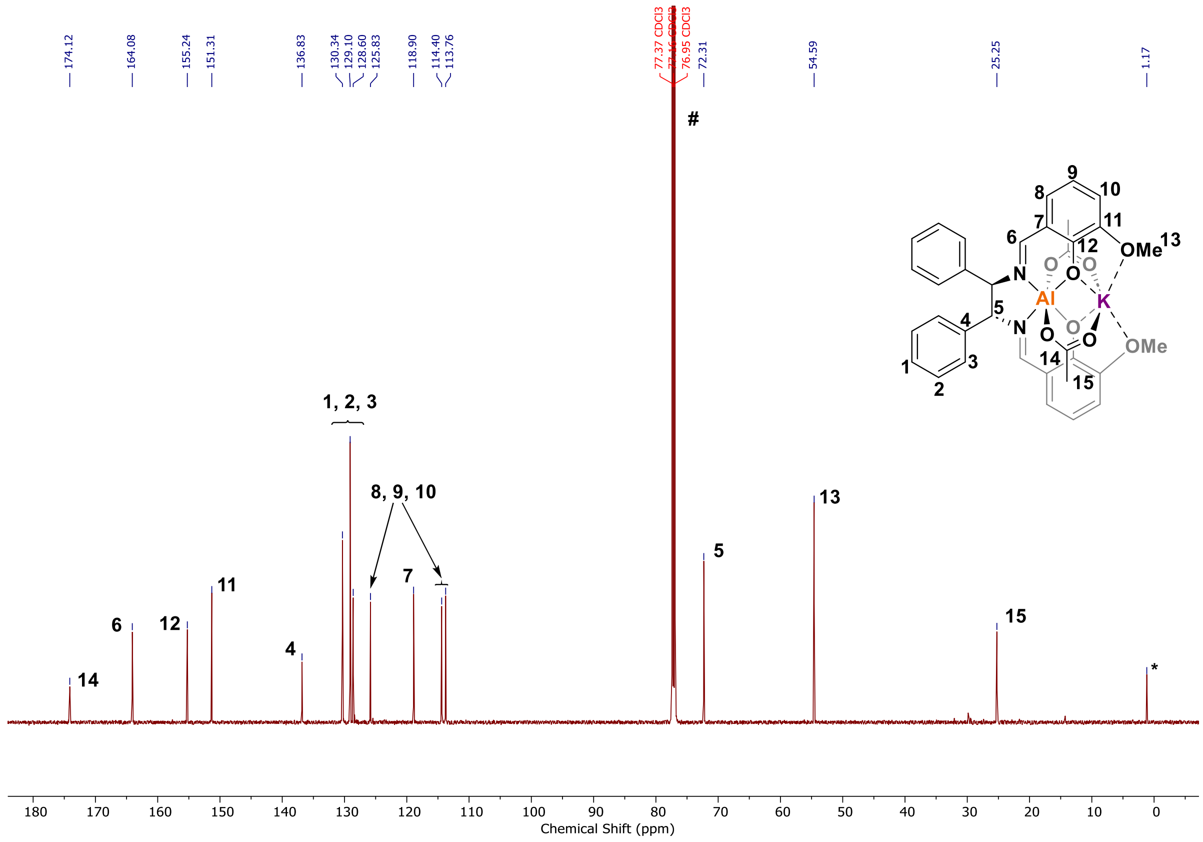

Figure S17. ^{13}C NMR Spectrum of complex **3** ($CDCl_3$(#), 298 K, 151 MHz) *: impurity from grease.

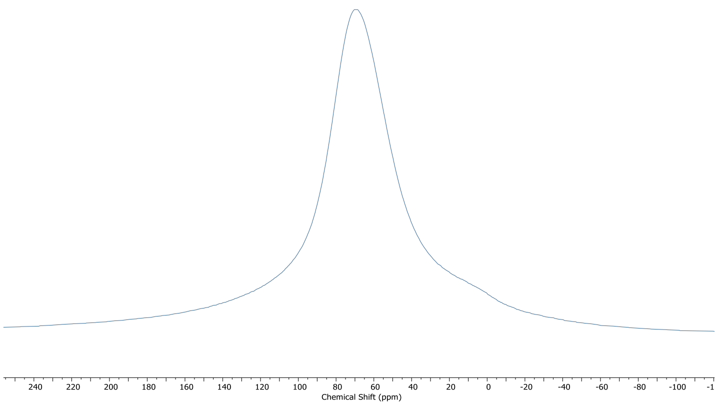

Figure S18. ^{27}Al NMR spectrum of complex **3** ($CDCl_3$, 298 K, 104 MHz).

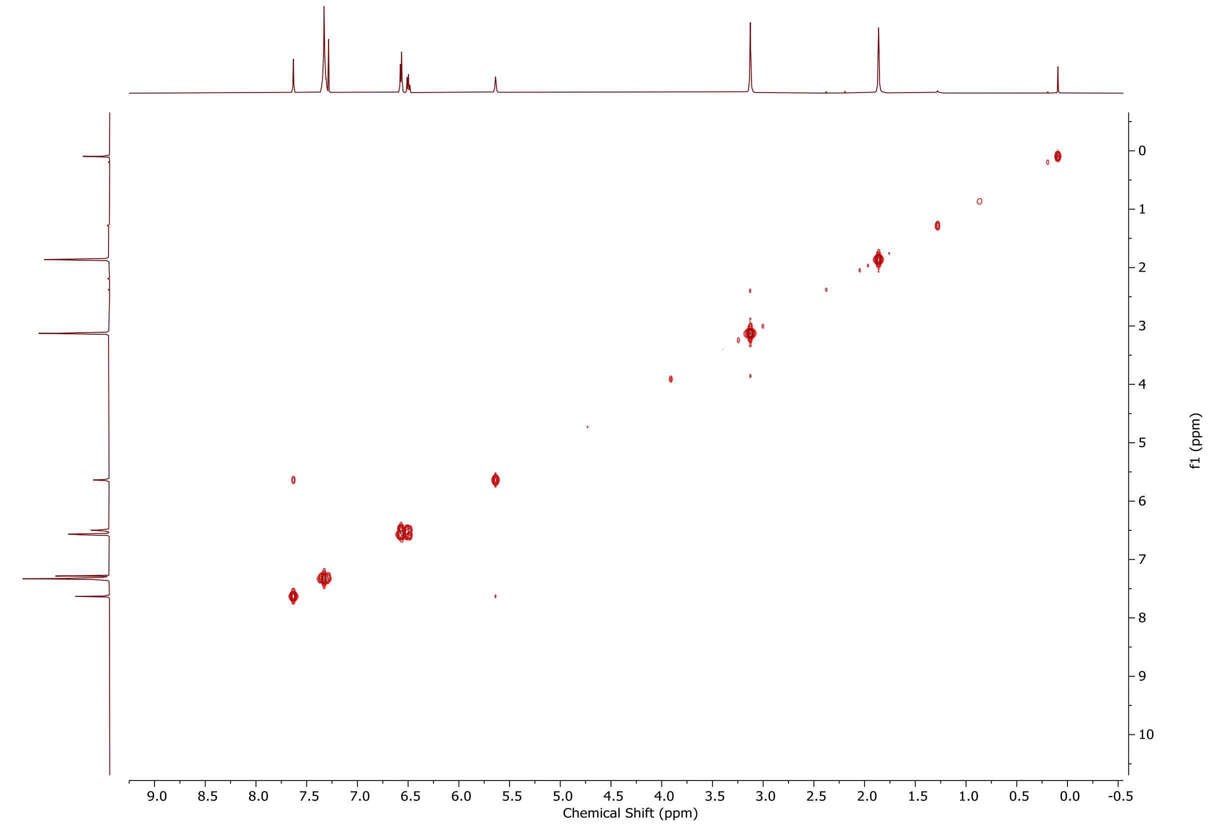

Figure S19. ^{1}H COSY NMR Spectrum of complex **3** ($CDCl_3$, 298 K, 400 MHz)

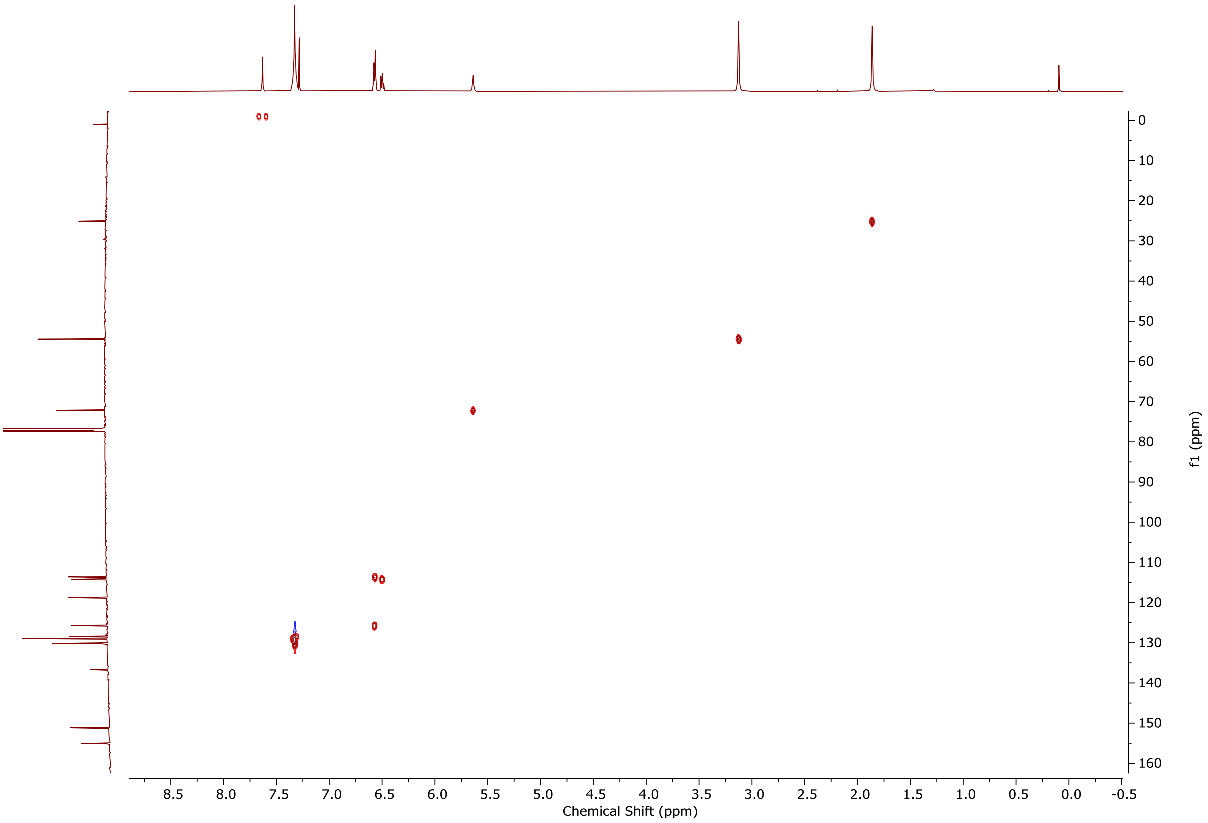

Figure S20. ^{1}H-^{13}C HSQC NMR Spectrum of complex **3** ($CDCl_3$, 298 K)

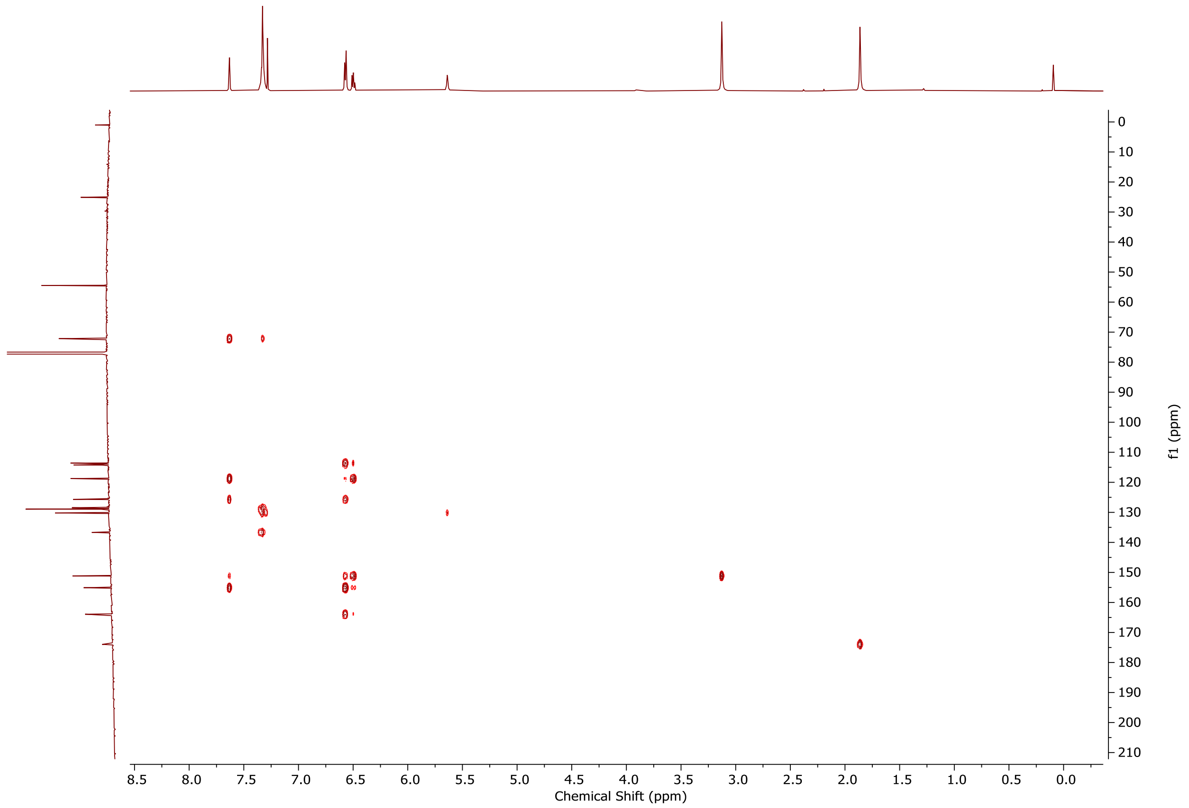

Figure S21. ^{1}H-^{13}C HMBC NMR Spectrum of complex **3** ($CDCl_3$, 298 K)

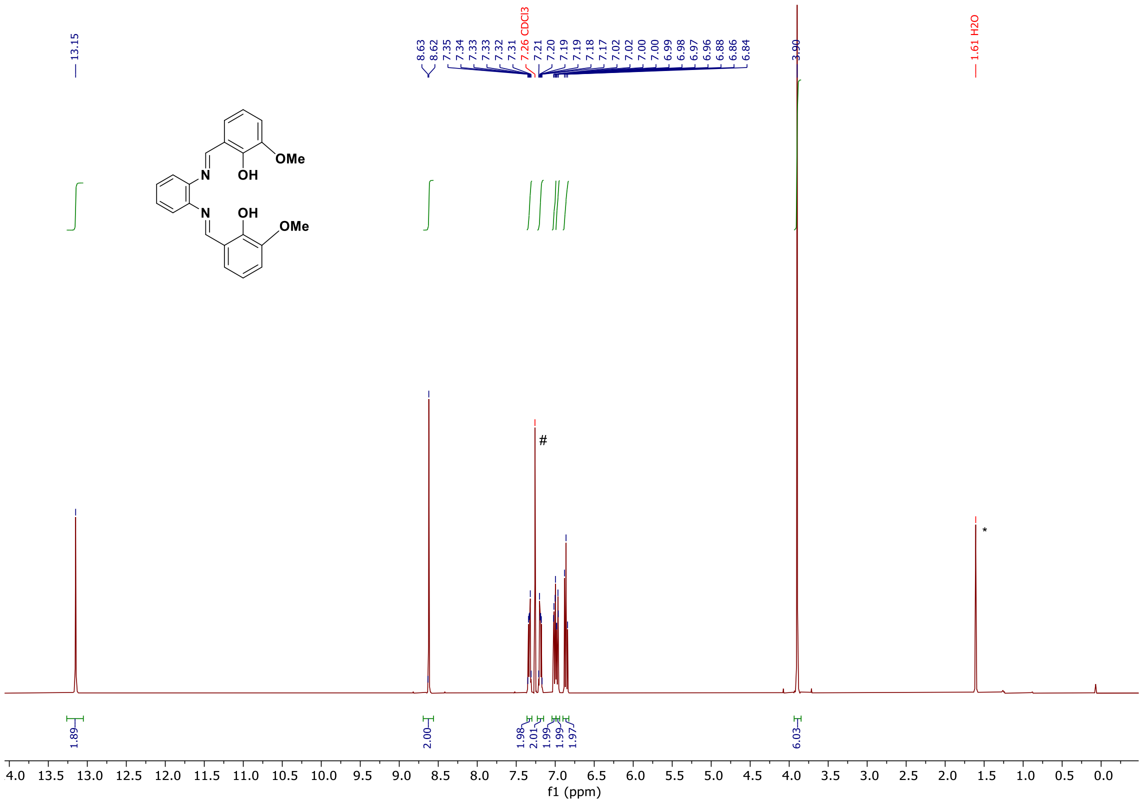

Figure S22. ^{1}H NMR Spectrum of $L_{Phen}H_2$ ($CDCl_3$(#), 298 K, 400 MHz) *: impurity from grease.

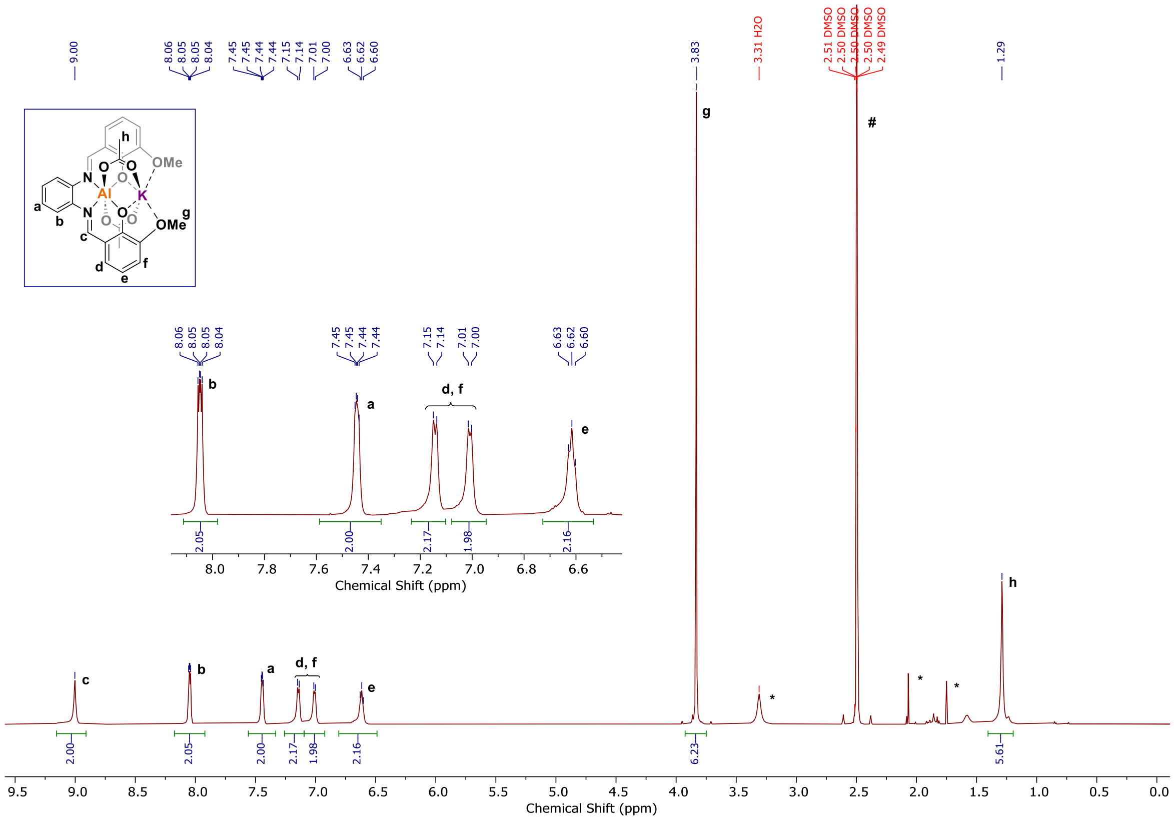

Figure S23. ^{1}H NMR spectrum of complex **4** (DMSO (#), 298 K, 400 MHz) *: impurities from hexane (in glovebox atmosphere) and water (from DMSO).

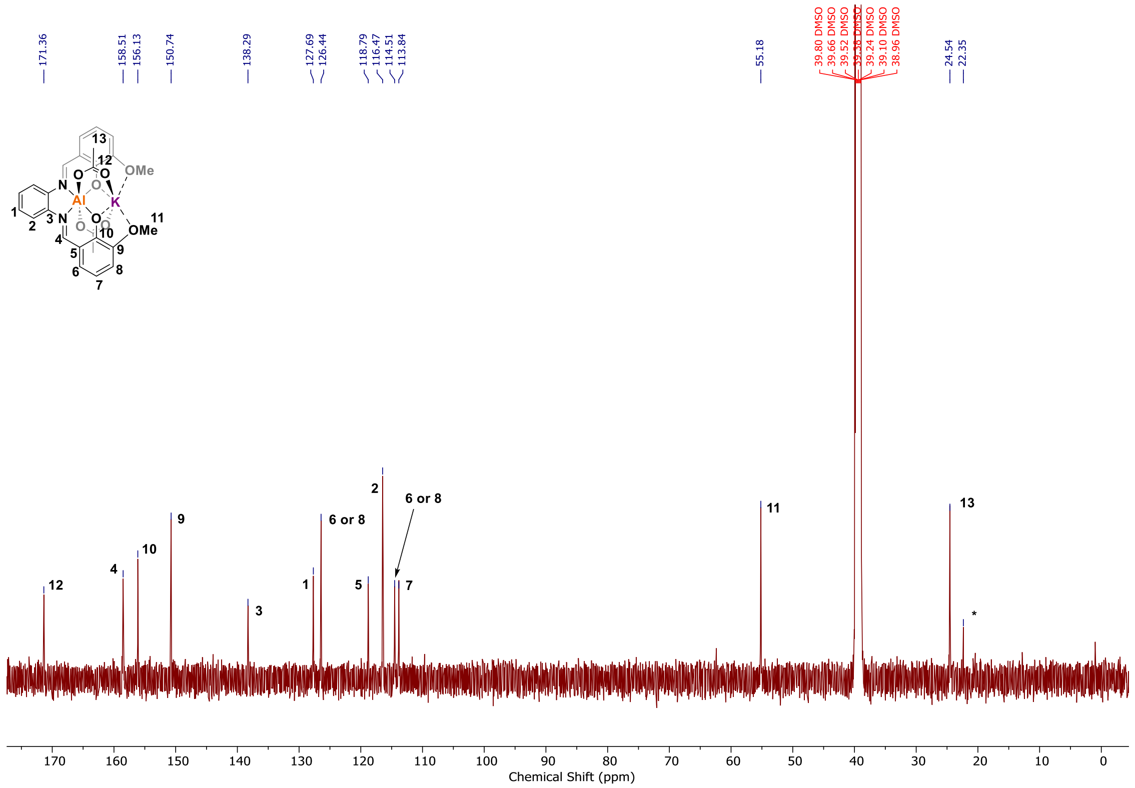

Figure S24. ^{13}C NMR Spectrum of complex **4** (DMSO(#), 298 K, 151 MHz) *: impurity from hexane

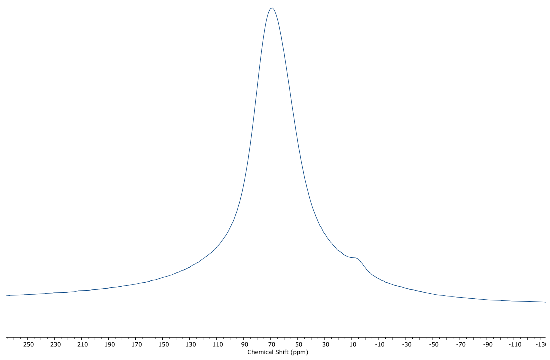

Figure S25. ^{27}Al NMR spectrum of complex **4** (DMSO, 298 K, 104 MHz).

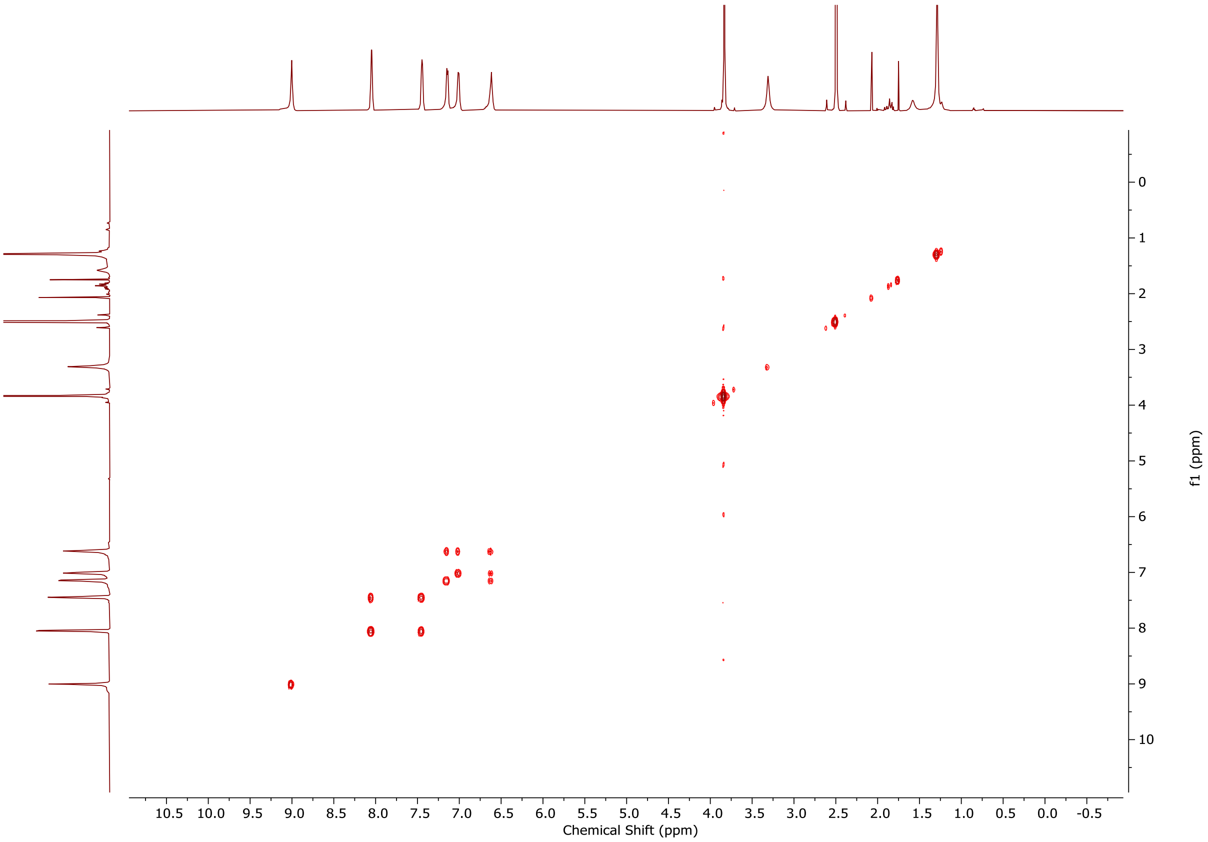

Figure S26. ^{1}H COSY NMR Spectrum of complex **4** (DMSO, 298 K, 400 MHz)

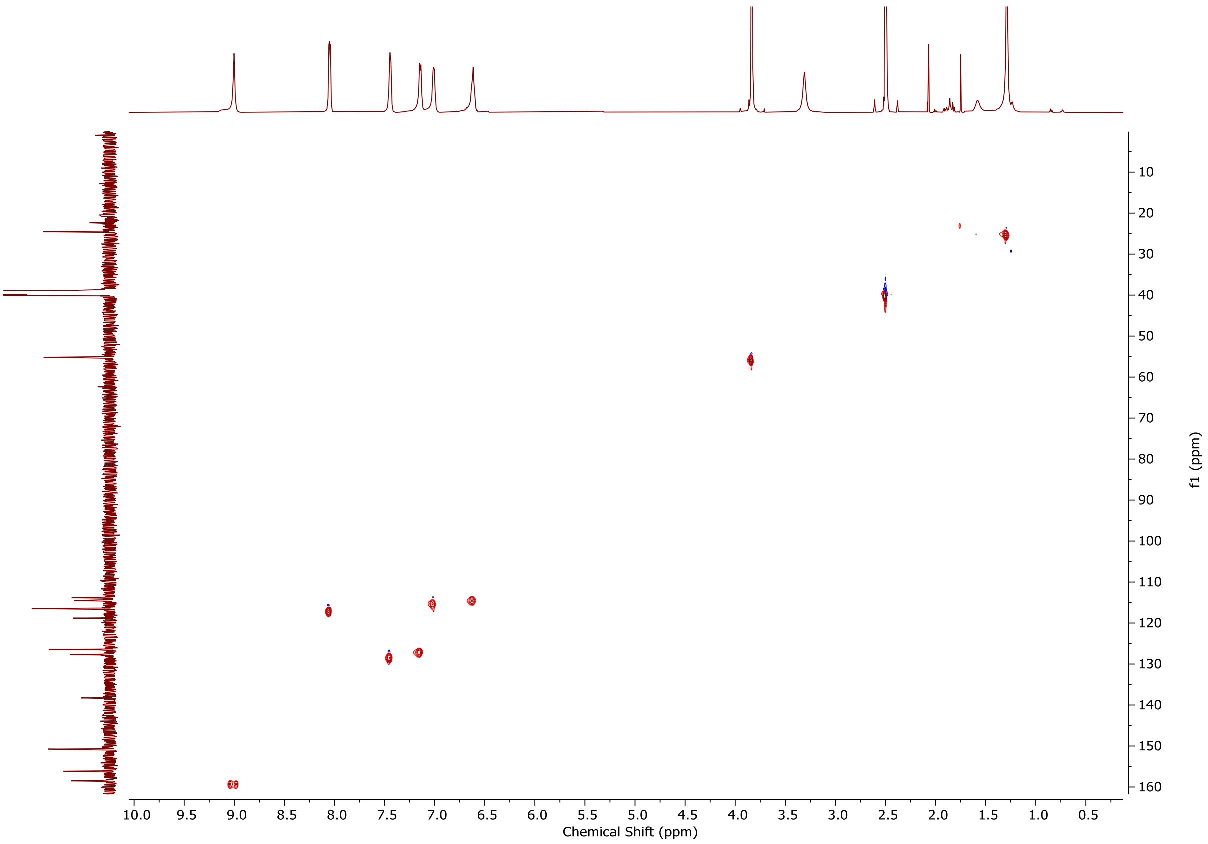

Figure S27. ^{1}H-^{13}C HSQC NMR Spectrum of complex **4** (DMSO, 298 K)

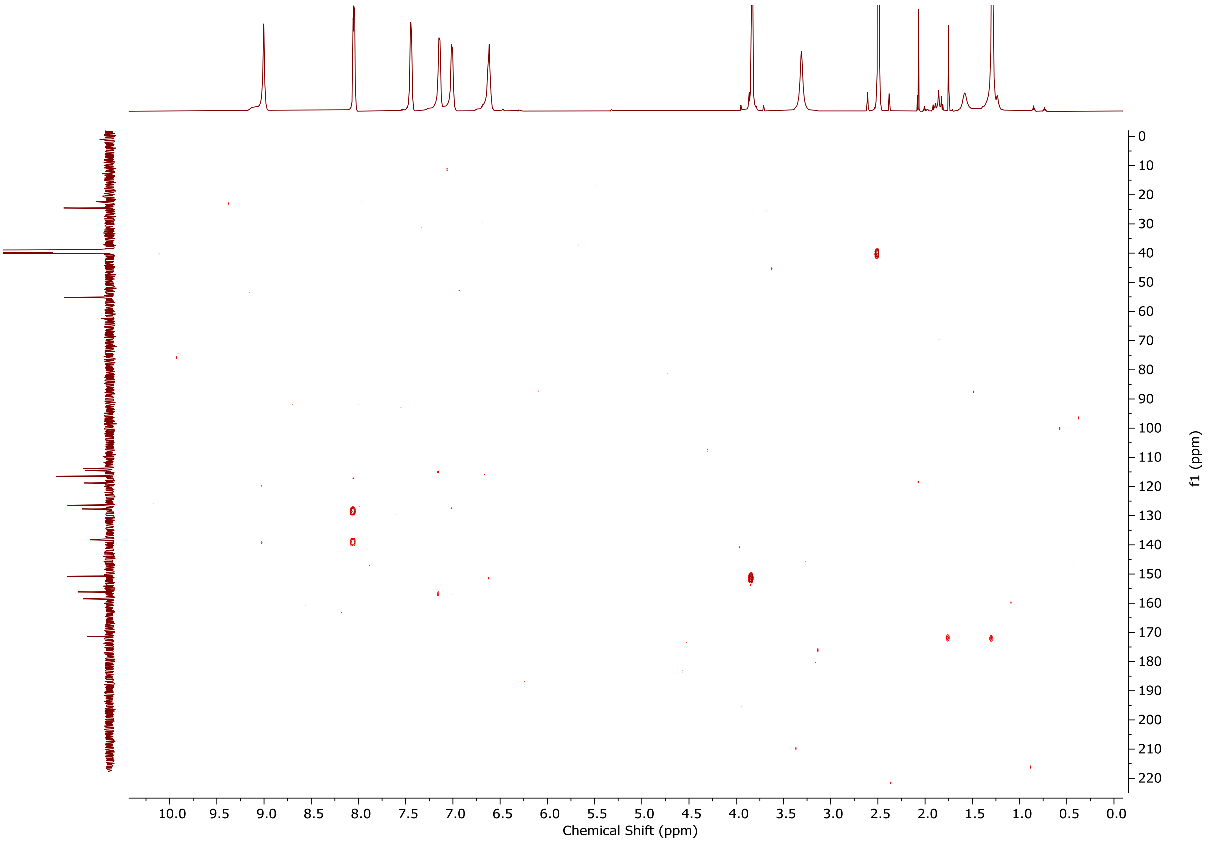

Figure S28. ^{1}H-^{13}C HMBC NMR Spectrum of complex **4** (DMSO, 298 K)

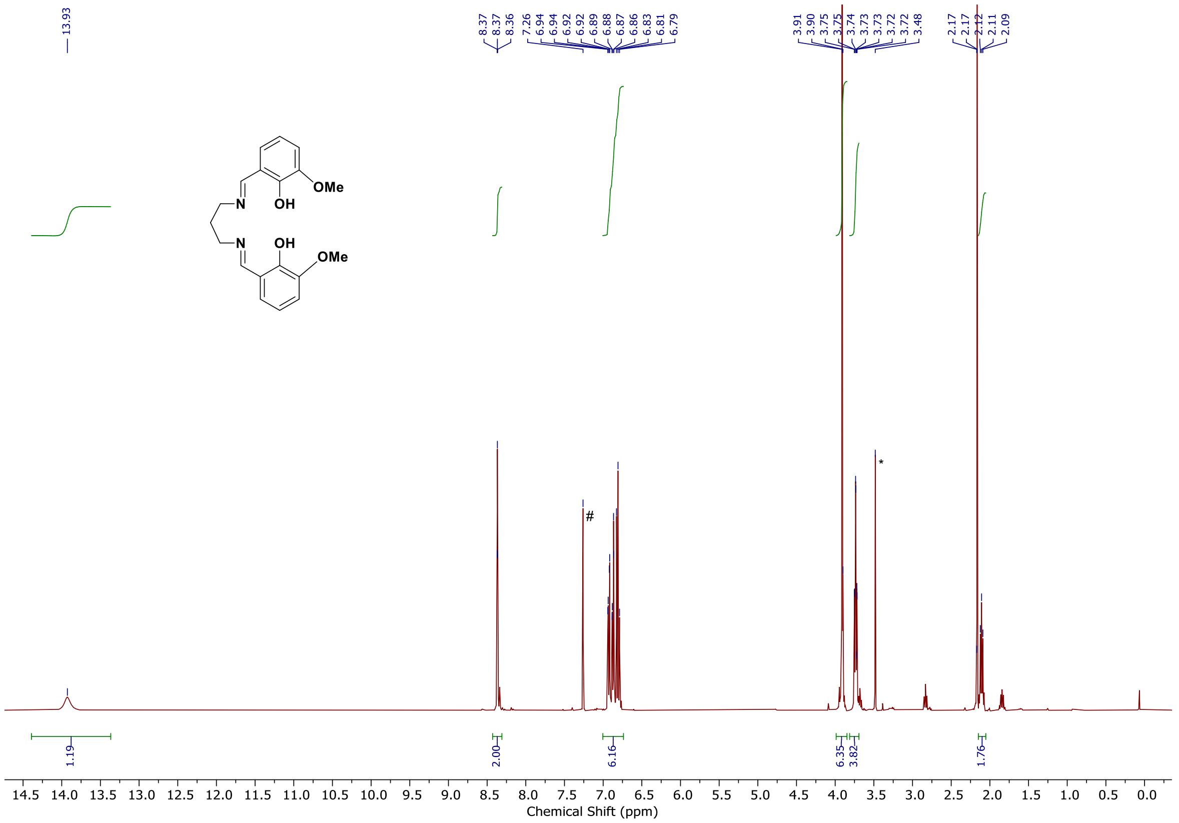

Figure S29. ^{1}H NMR Spectrum of $L_{Pro}H_2$ ($CDCl_3$(#), 298 K, 400 MHz) *: impurity from methanol.

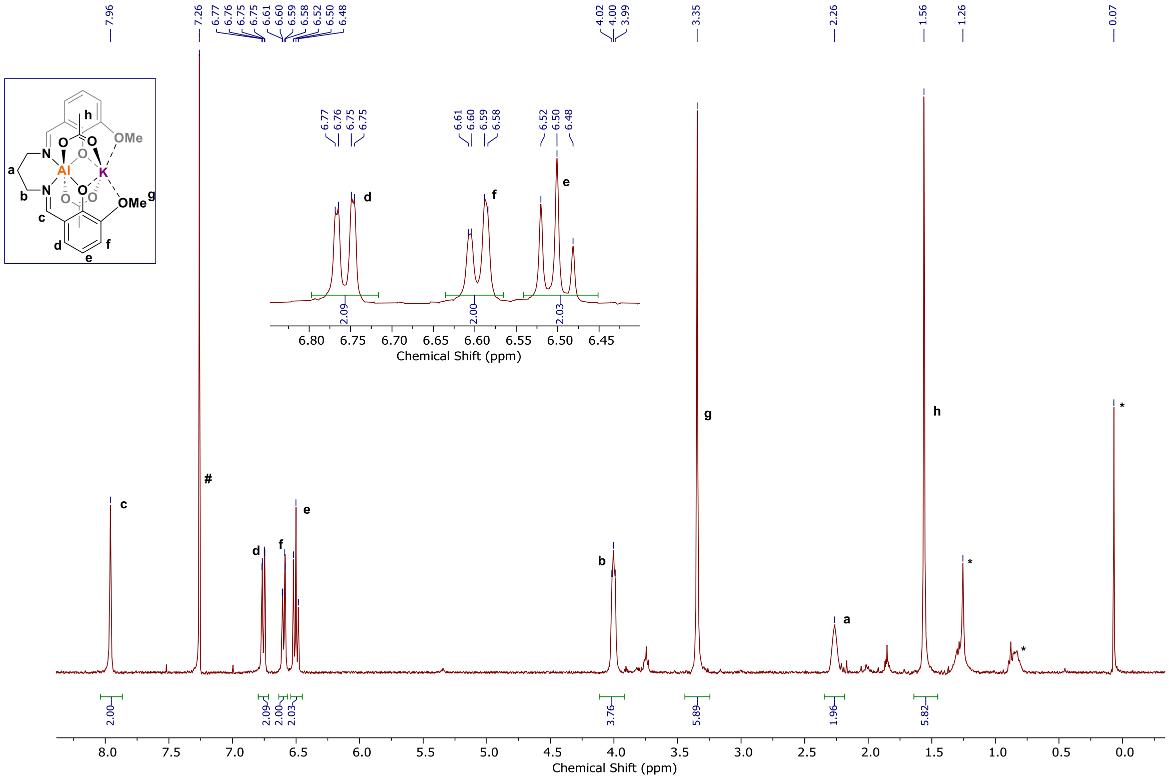

Figure S30. ^{1}H NMR spectrum of complex **5** (CDCl3(#), 298 K, 400 MHz) *: impurities from grease and hexane (in glovebox atmosphere).

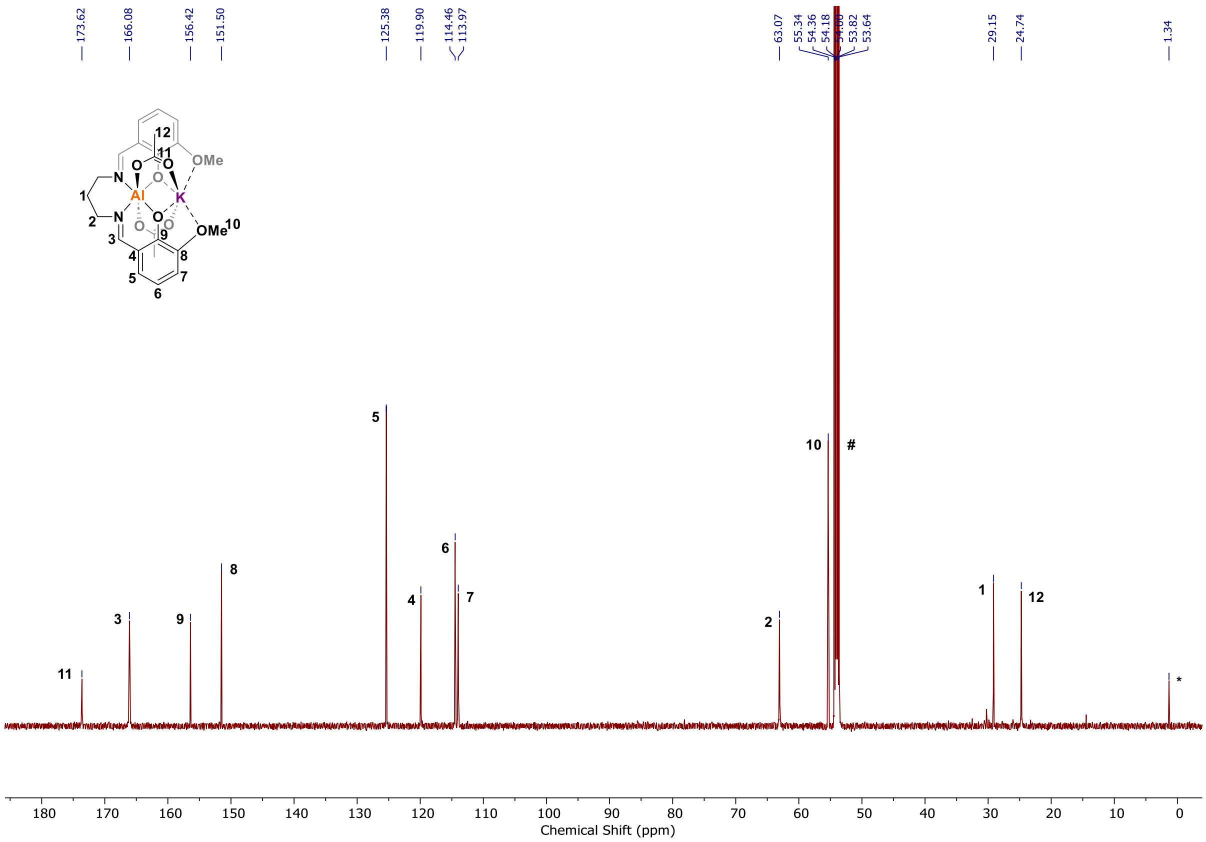

Figure S31. ^{13}C NMR Spectrum of complex **5** (CD_2Cl_2 (#), 298 K, 151 MHz) *: impurity from grease

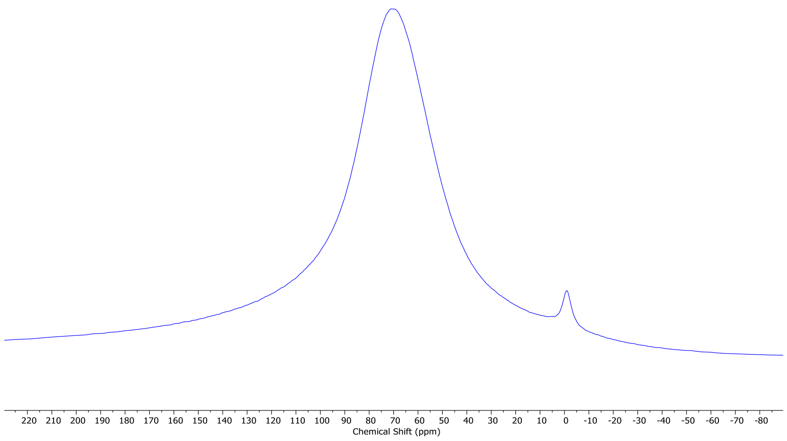

Figure S32. ^{27}Al NMR spectrum of complex **5** ($CDCl_3$, 298 K, 104 MHz).

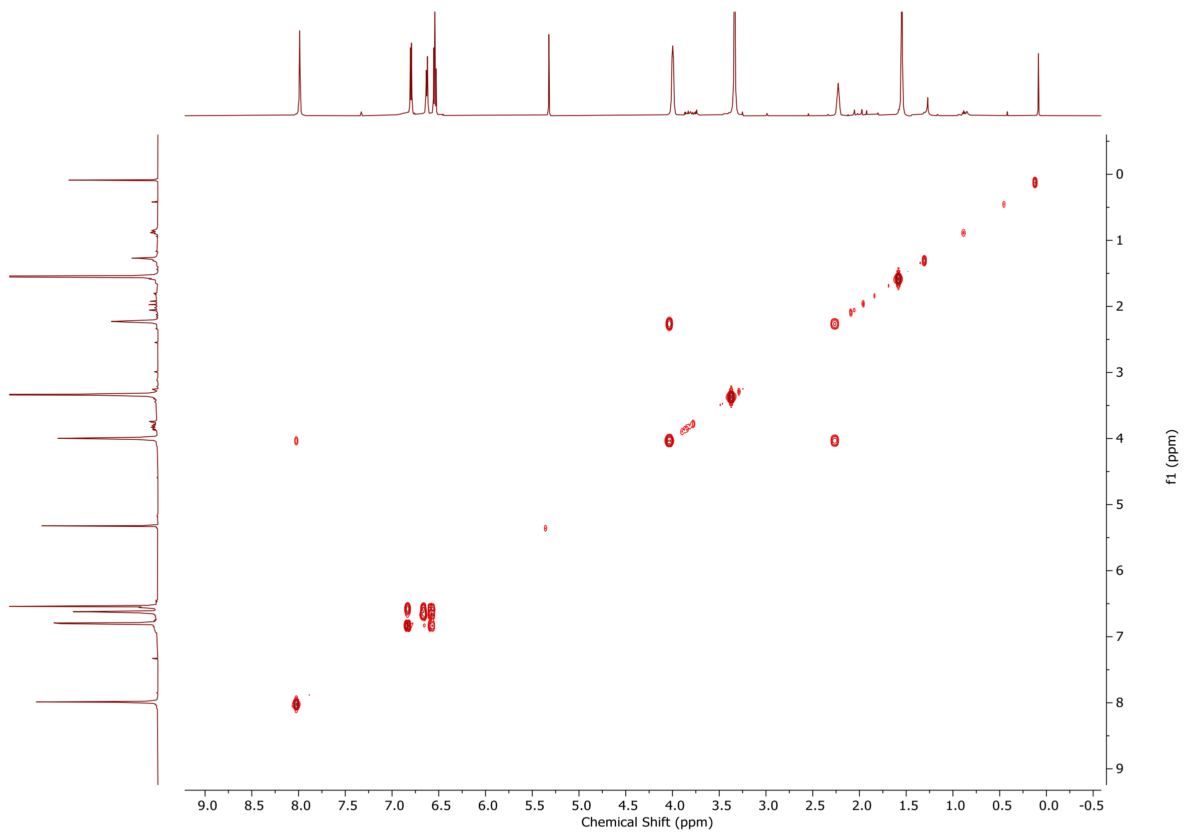

Figure S33. ^{1}H COSY NMR Spectrum of complex **5** (CD_2Cl_2, 298 K, 400 MHz)

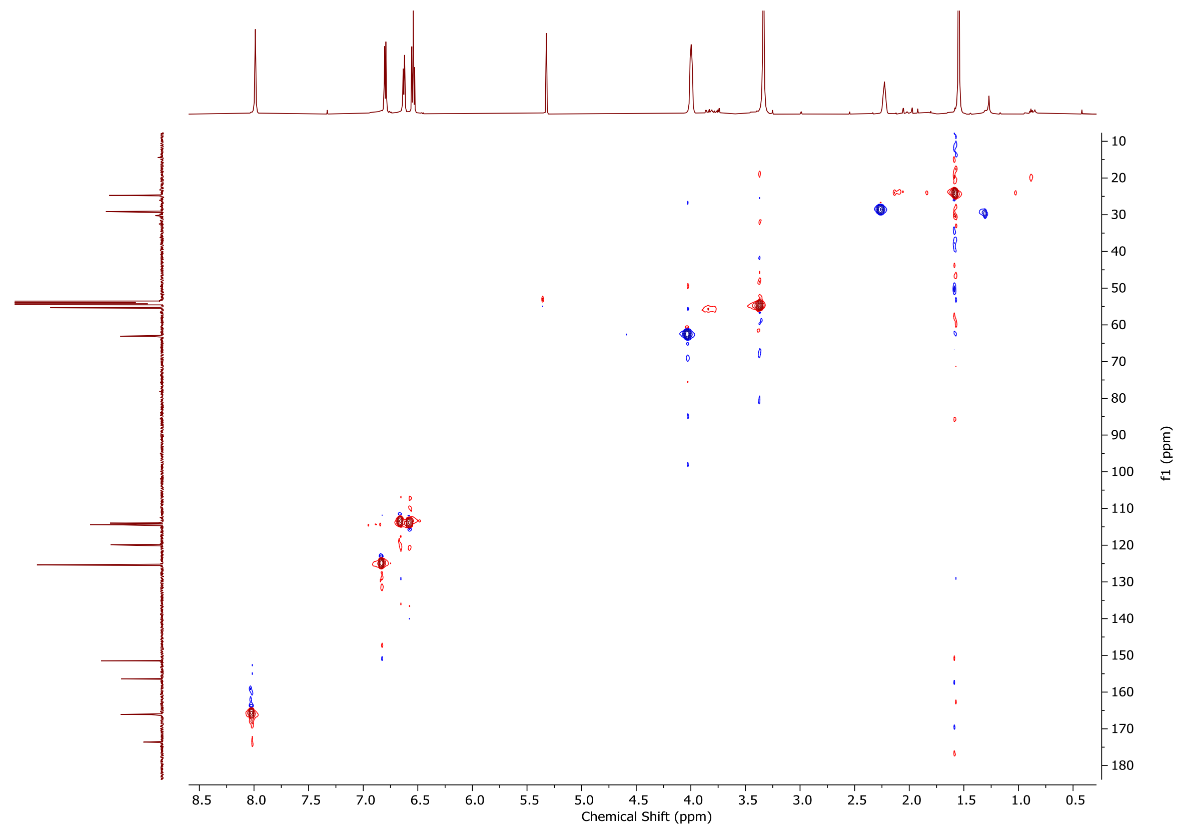

Figure S34. ^{1}H-^{13}C HSQC NMR Spectrum of complex **5** (CD_2Cl_2, 298 K)

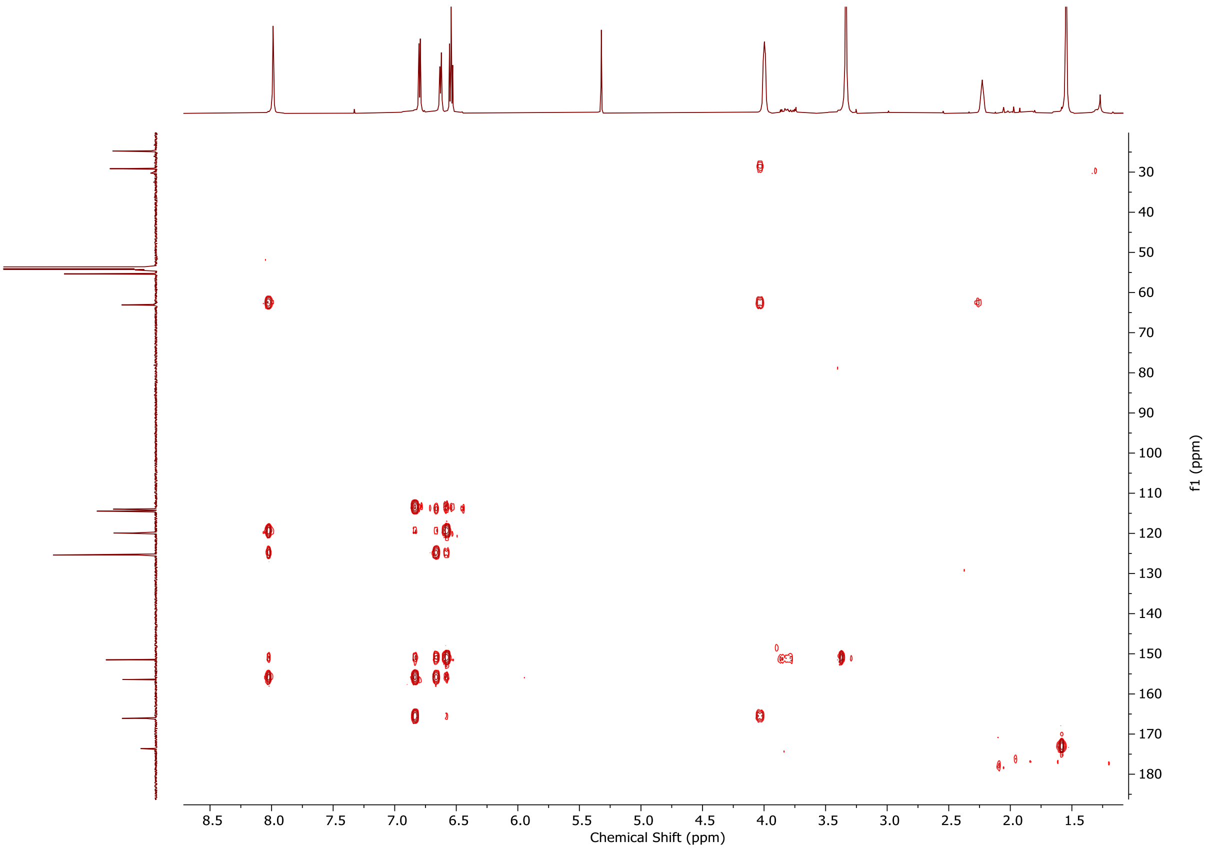

Figure S35. ^{1}H-^{13}C HMBC NMR Spectrum of complex **5** (CD_2Cl_2, 298 K)

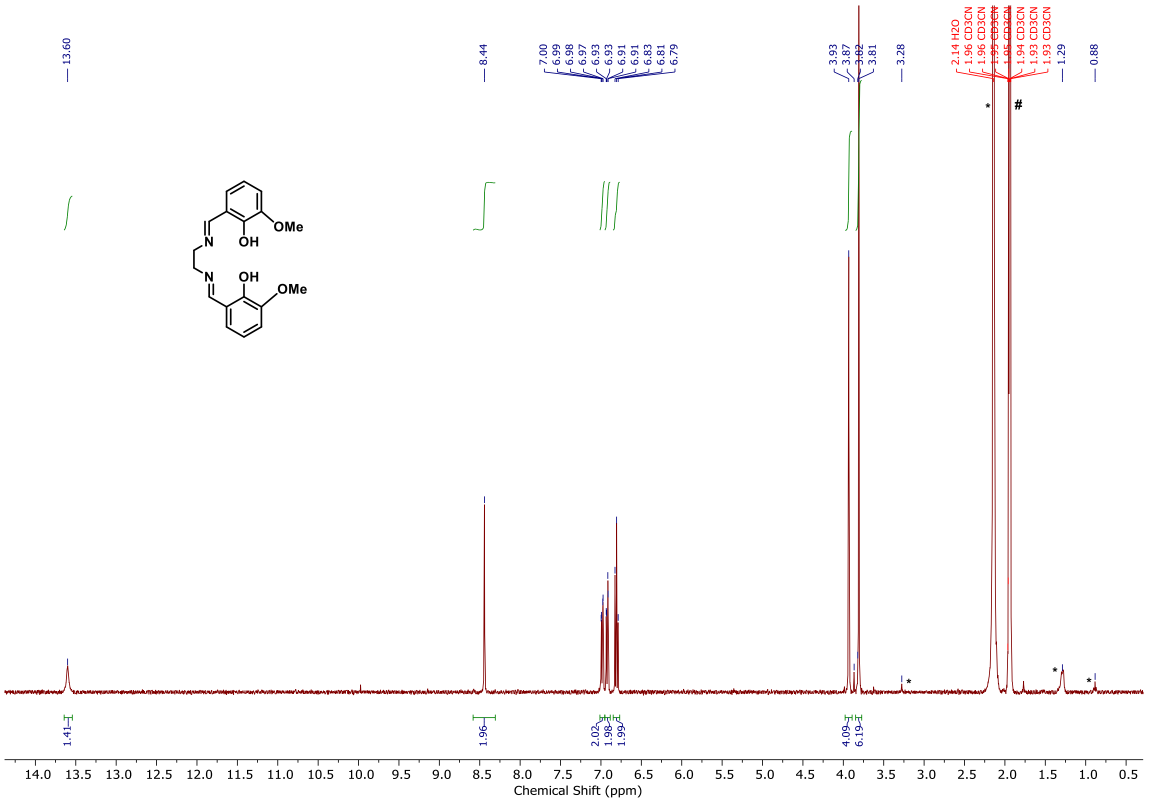

Figure S36. ^{1}H NMR Spectrum of $L_{Et}H_2$ (CD_3CN(#), 298 K, 400 MHz) *: impurity from water.

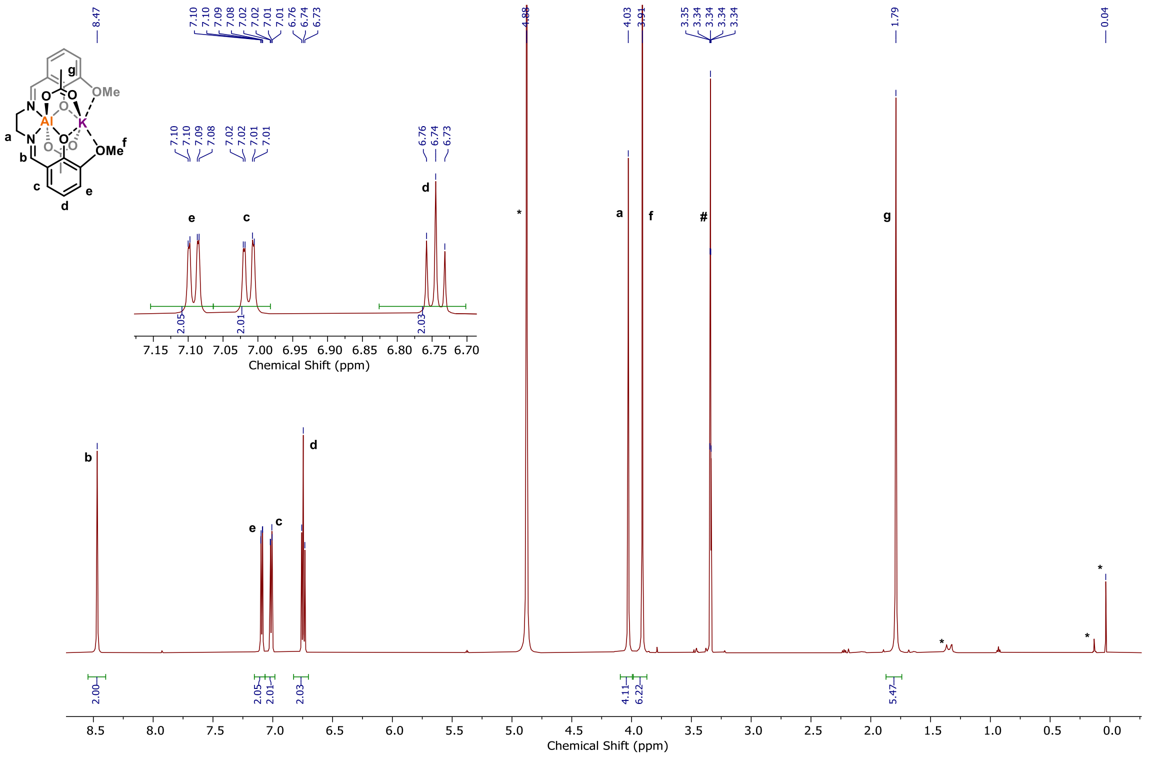

Figure S37. ^{1}H NMR spectrum of complex **6** (MeOD-d[4](#), 298 K, 400 MHz) *: impurities from grease, hexane and water.

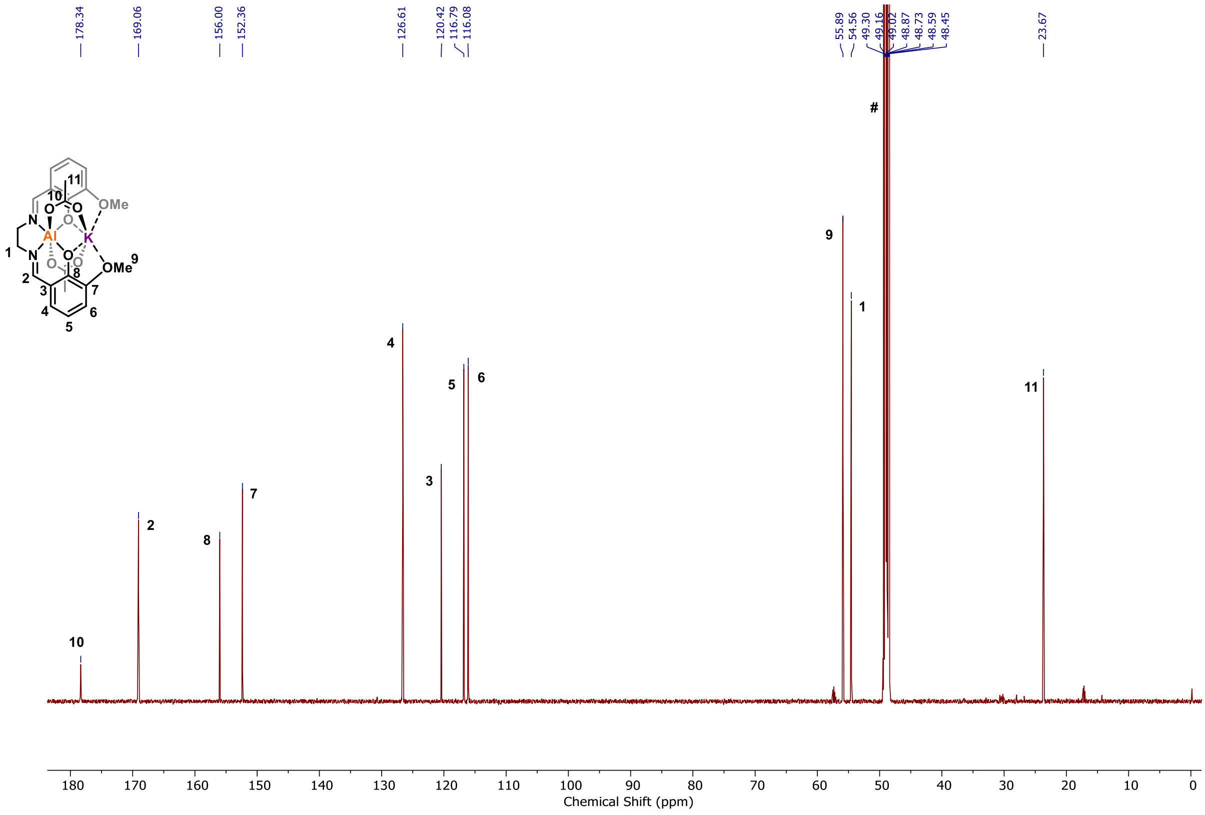

Figure S38. ^{13}C NMR Spectrum of complex **6** (MeOD-d[4](#), 298 K, 151 MHz)

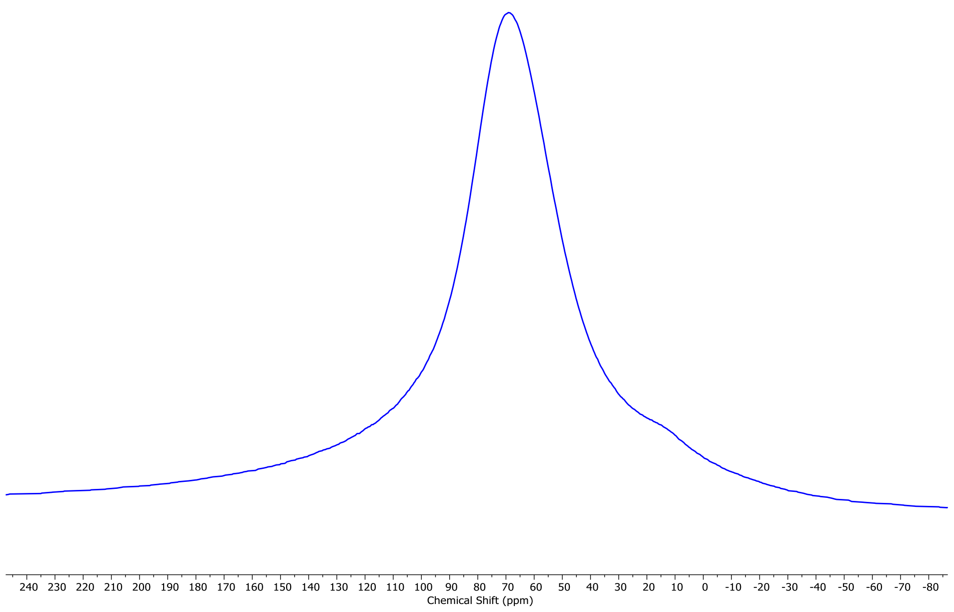

Figure S39. ^{27}Al NMR spectrum of complex **6** (MeOD-d^4, 298 K, 104 MHz).

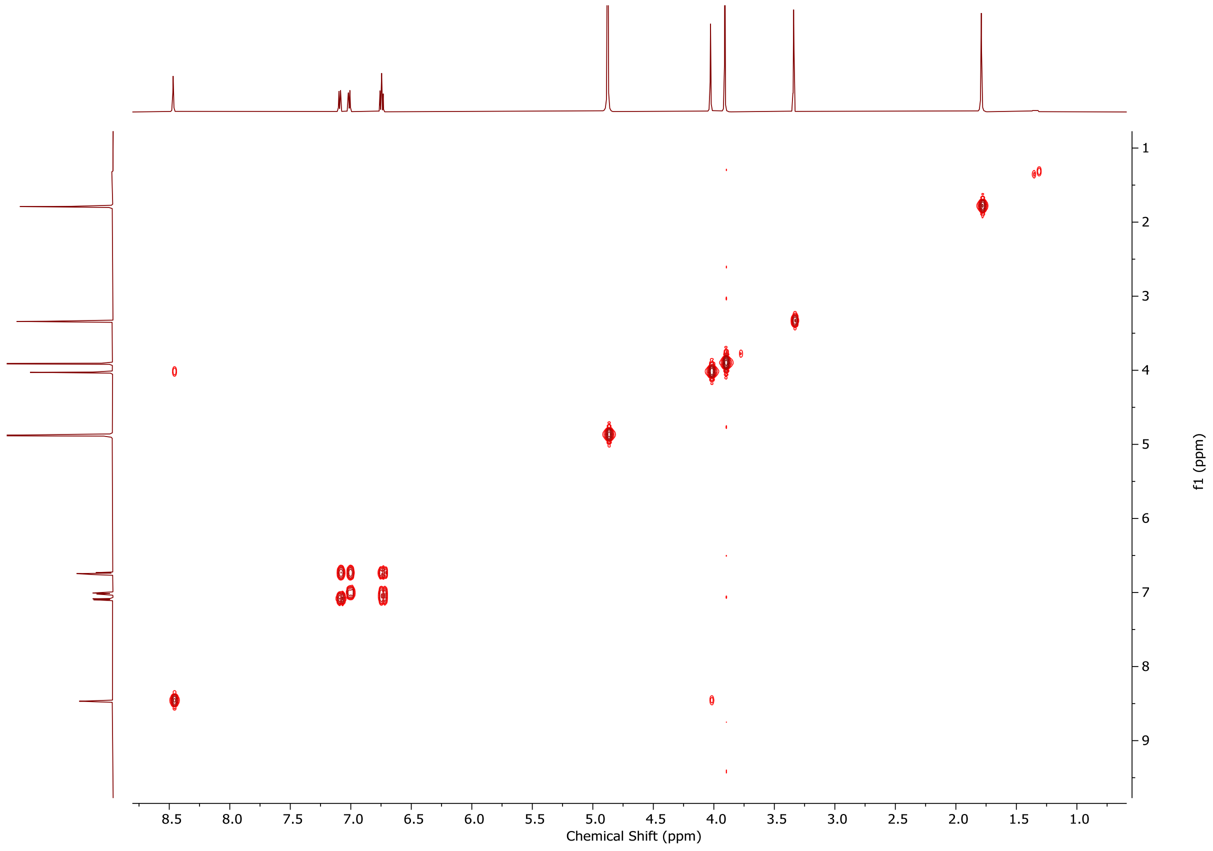

Figure S40. ^{1}H COSY NMR Spectrum of complex **6** (MeOD-d^4, 298 K, 400 MHz)

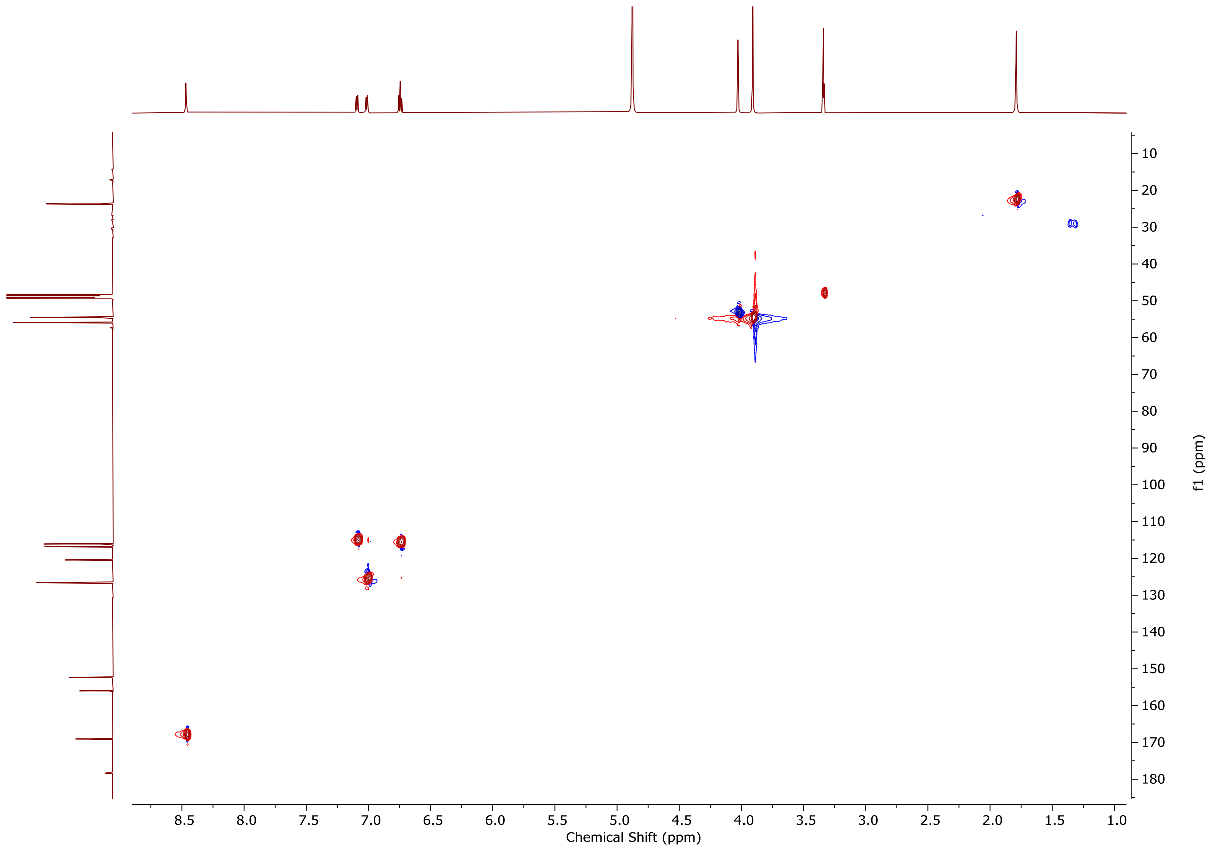

Figure S41. ^{1}H-^{13}C HSQC NMR Spectrum of complex **6** (MeOD-$d^{4}{}_{2}$, 298 K)

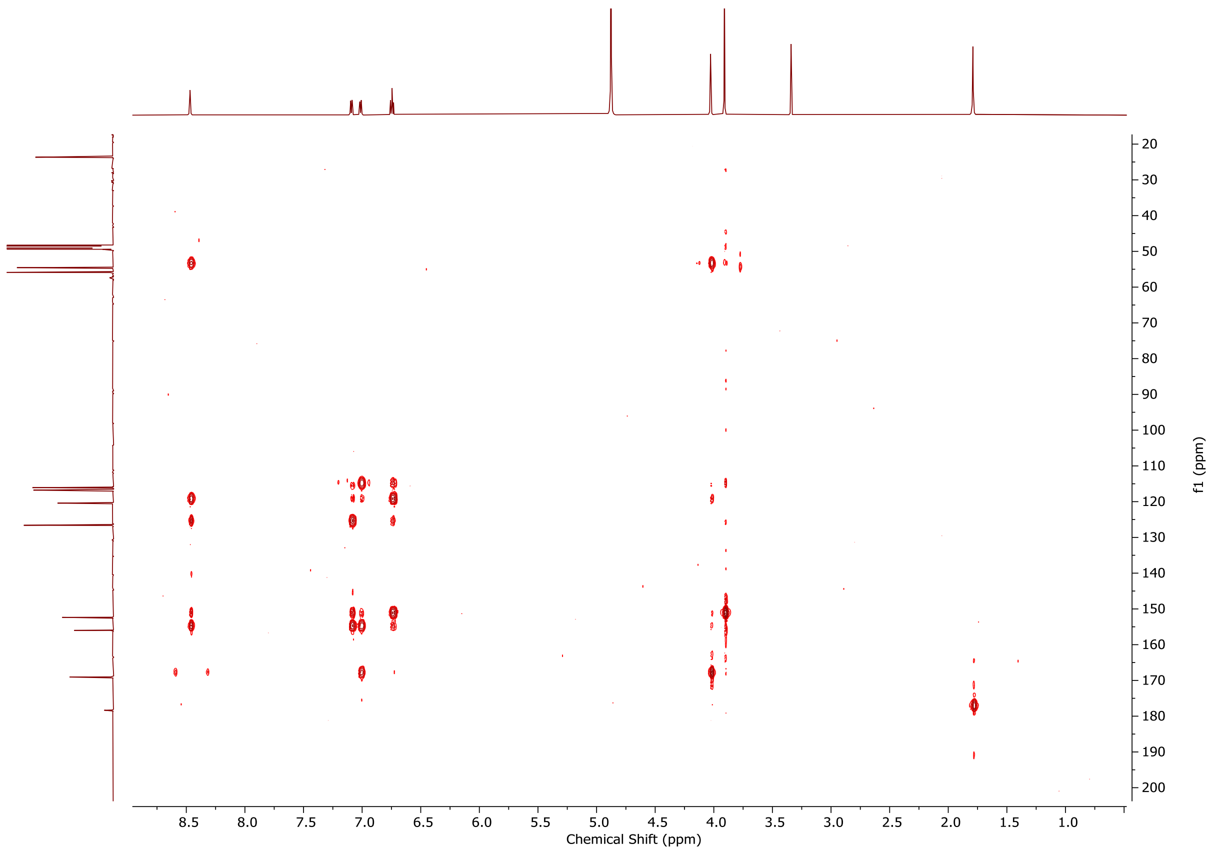

Figure S42. ^{1}H-^{13}C HMBC NMR Spectrum of complex **6** (MeOD-d^{4}, 298 K)

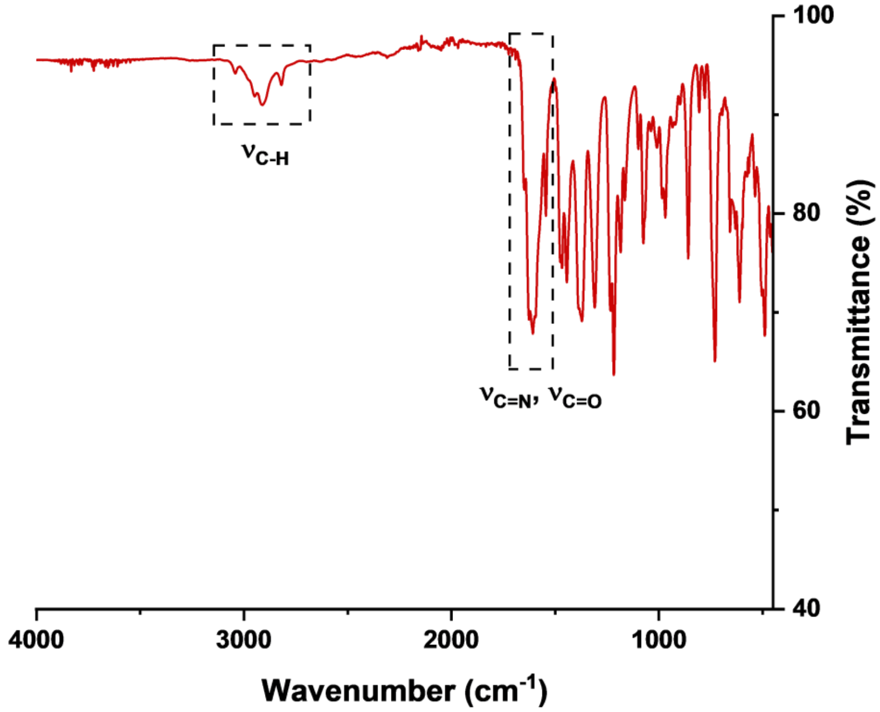

Figure S43. IR spectrum of complex **1**.

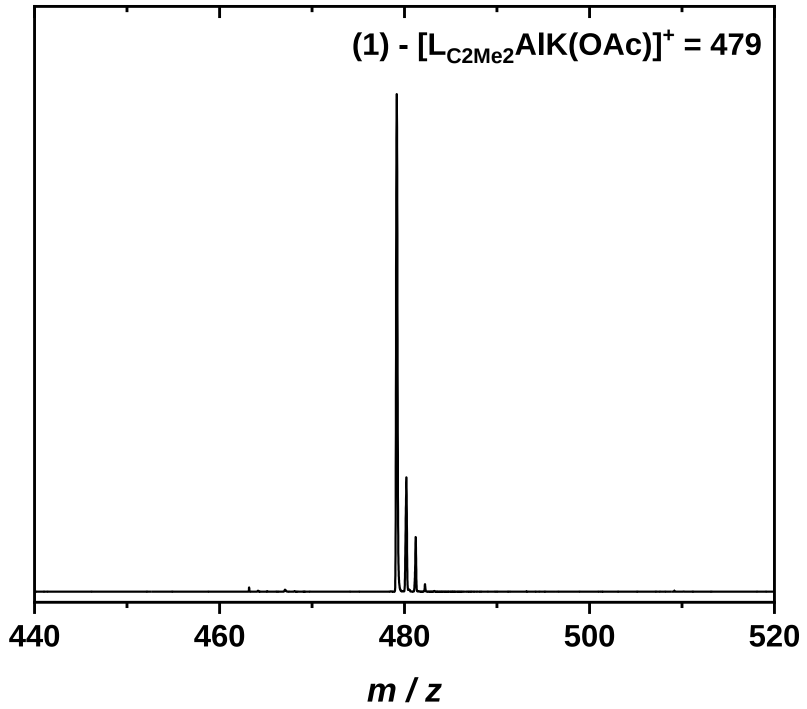

Figure S44. MALDI-TOF spectrum of complex **1**.

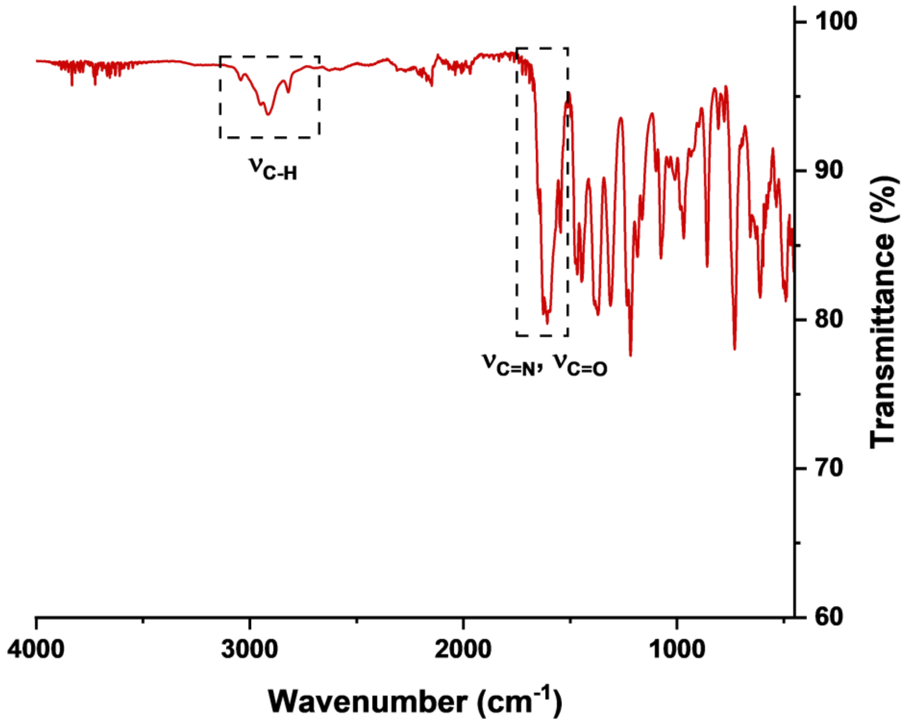

Figure S45. IR spectrum of complex **2**.

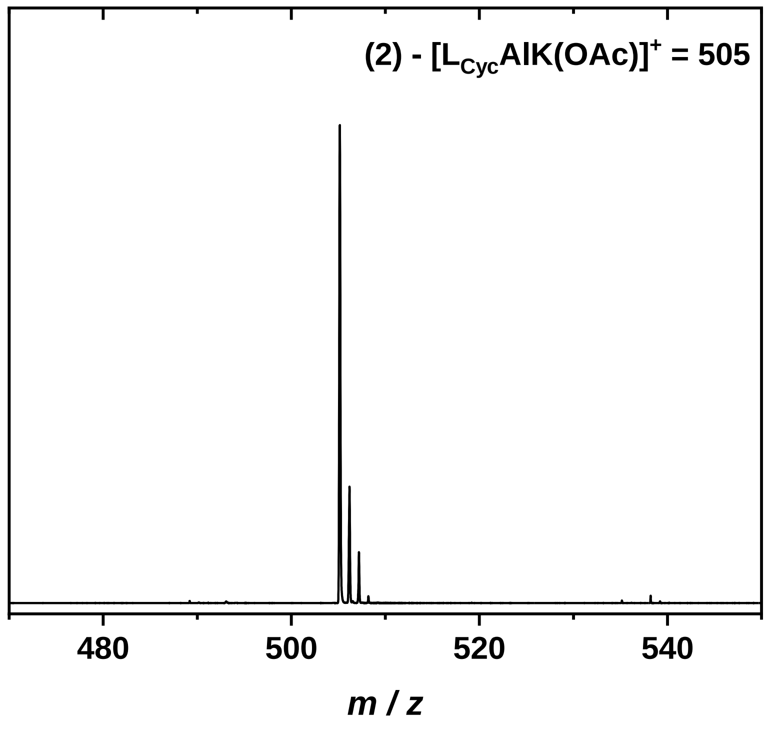

Figure S46. MALDI-ToF spectrum of complex **2**.

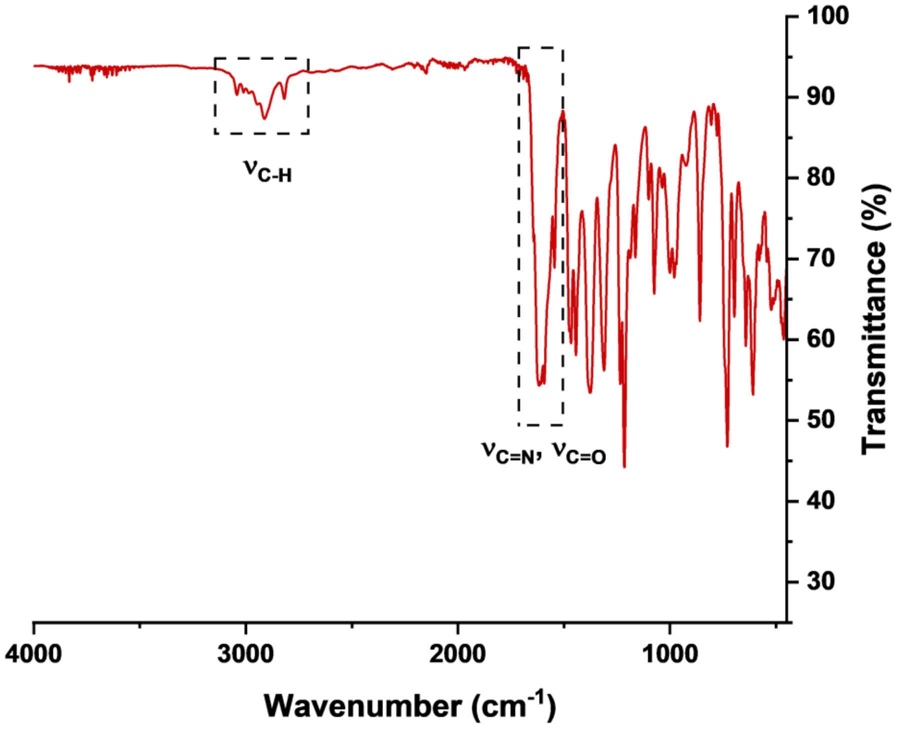

Figure S47. IR spectrum of complex **3**.

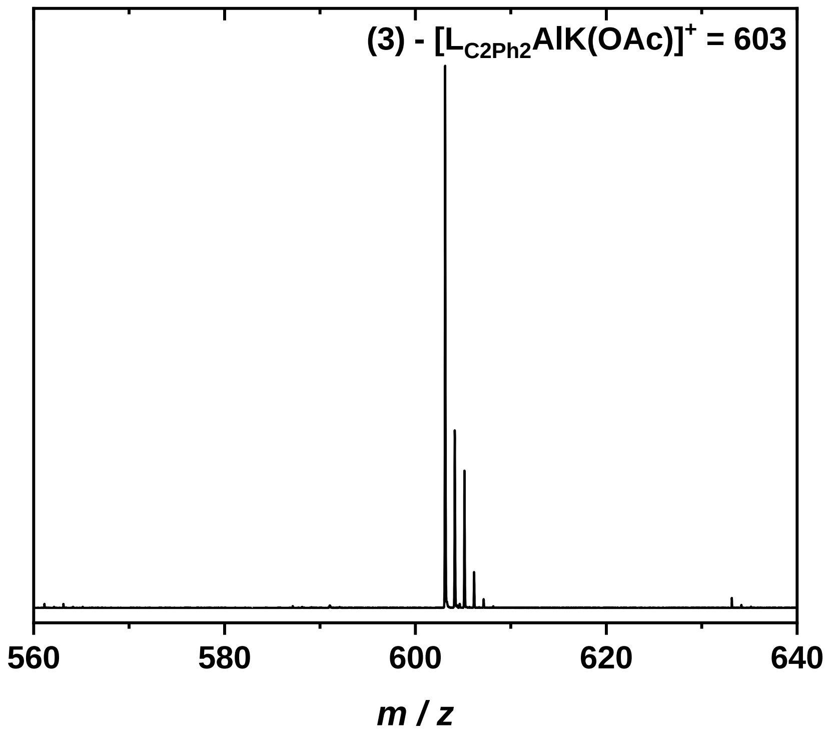

Figure S48. MALDI-ToF spectrum of complex **3**.

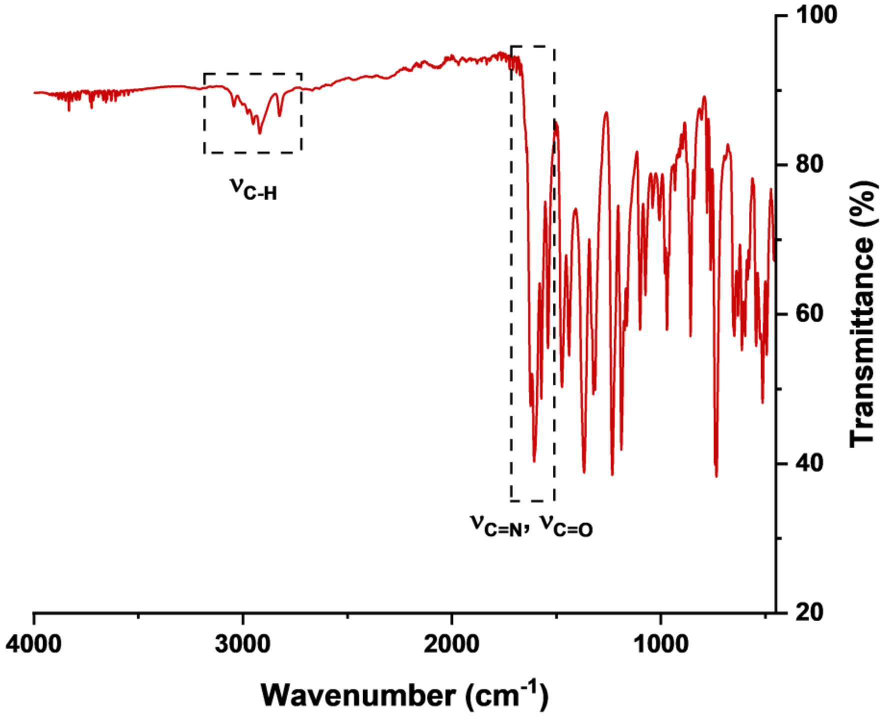

Figure S49. IR spectrum of complex **4**.

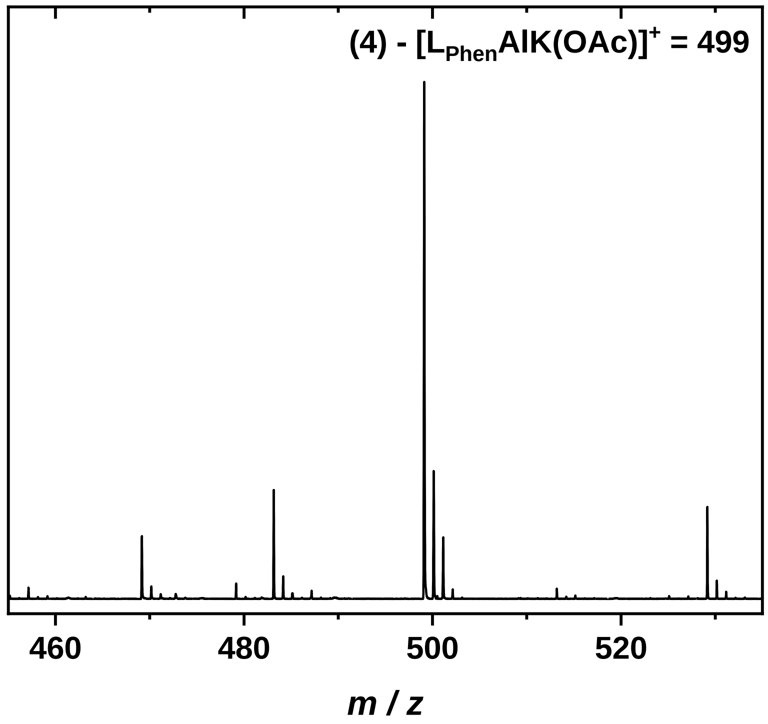

Figure S50. MALDI-ToF spectrum of complex **4**. Sample did not fly well possibly due to low sample loading as a consequence of poor solubility.

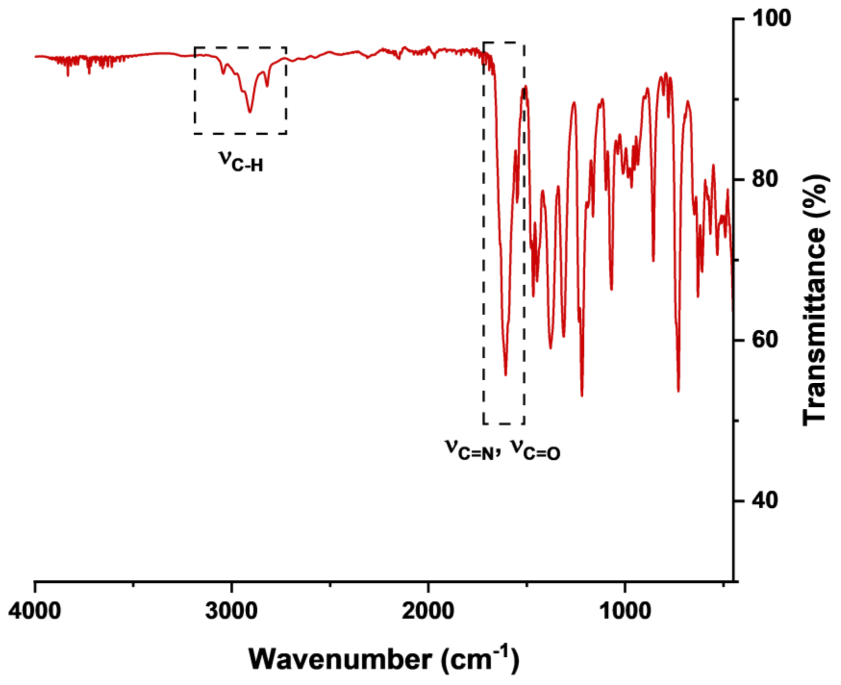

Figure S51. IR spectrum of complex **5**.

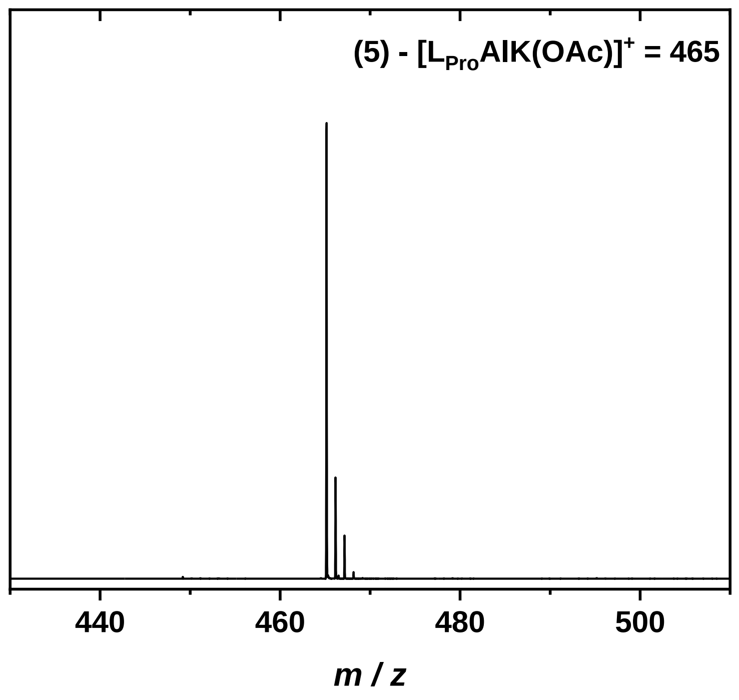

Figure S52. MALDI-ToF spectrum of complex **5**.

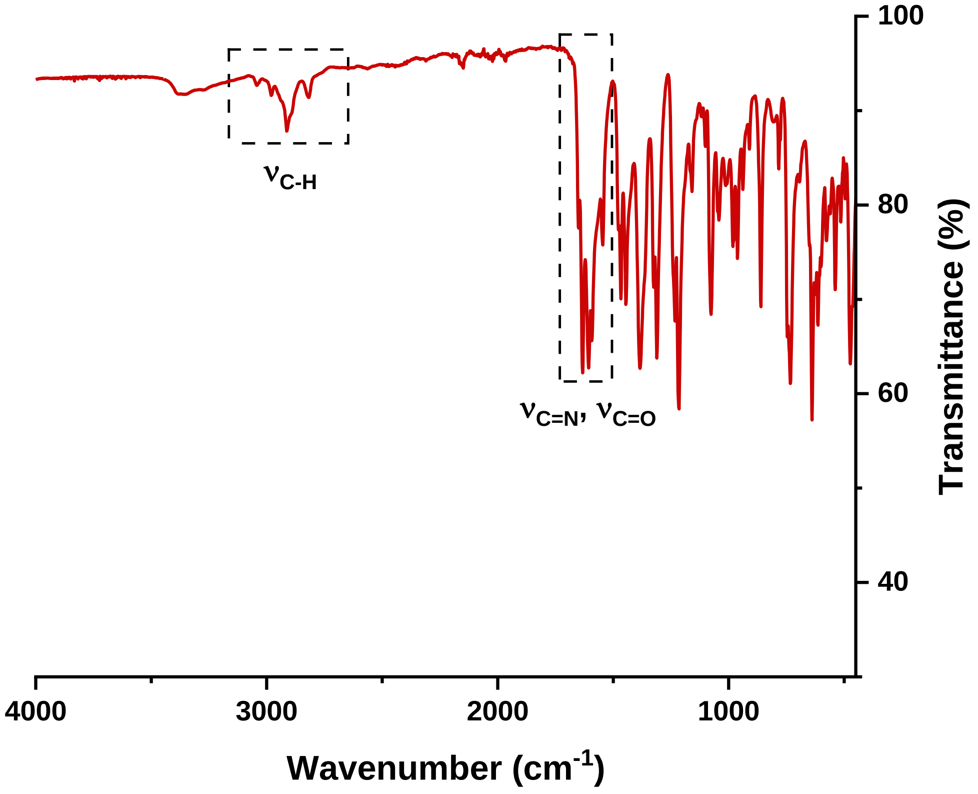

Figure S53. IR spectrum of complex **6**.

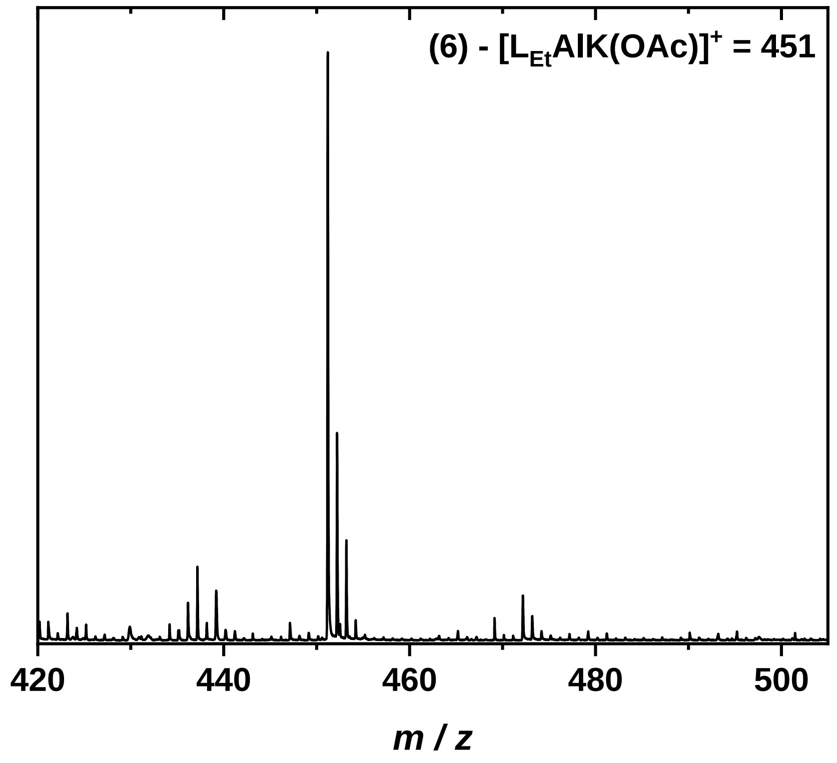

Figure S54. MALDI-ToF spectrum of complex **6**.

Table S1. X-ray crystallography data for $L_{C2Me2}AlK(OAc)_2$ (**1**)

Bond	Bond Length (Å)
K(1)-Al(1)	3.5789(8)
K(1)-O(2)	2.6641(18)
K(1)-O(4)	2.9294(18)
Al(1)-O(1)	1.9174(17)
Al(1)-O(3)	1.8915(15)
O(1)-C(1)	1.271 (3)
O(2)-C(1)	1.226(3)
O(3)-C(3)	1.273(2)
O(4)-C(3)	1.229(3)

Local Code<br>CCDC Deposition Number	008ff22<br>2280611
Crystal Data	
Chemical formula	$C_{24}H_{28}AlKN_2O_8$
M_r	538.56
Crystal system, space group	Triclinic, $P\bar{1}$
a, b, c (Å)	9.8713(4)<br>11.2080(4)<br>11.8060(4)
α, β, γ, (°)	84.516(3)<br>78.103(3)<br>81.516(3)
V (Å^3)	1261.28(8)
Z	2
Radiation type	Cu *Kα*
μ (mm^{-1})	1.54184
Crystal size (mm)	0.14 x 0.12 x 0.10
Data collection	
Absorption correction	CrysAlisPro 1.171.41.117a (Rigaku Oxford Diffraction, 2021)<br>Empirical absorption correction using spherical harmonics, implemented in SCALE3 ABSPACK scaling algorithm.
T_{min}, T_{max}	0.94043, 1.00000
No. of measured, independent and observed [$I > 2\sigma(I)$] reflections	18706, 5199, 4292
R_{int}	0.0264
$(\sin\theta/\lambda)_{max}$ (Å^{-1})	0.630
Refinement	
$R[F^2 > 2\sigma(F^2)]$, $wR(F^2)$, S	0.044, 0.124, 1.04
No of reflections	18706
No. of parameters	370
No. of restraints	0
$\Delta\rangle_{max}$, $\Delta\rangle_{min}$ (e Å^{-3})	0.44, -0.36

Table S2. ^{1}H Chemical Shifts for the reactant and products of CHO/PA copolymerization in $CDCl_3$.

Component	PA	PA in PCHPE	MES internal standard	CHO in ester linkage	CHO in ether linkage
Chemical shift (ppm)	8.06-8.00<br>7.94-7.88	7.63-7.52<br>7.44-7.34	6.78-6.81	5.22-5.04	3.80-3.20

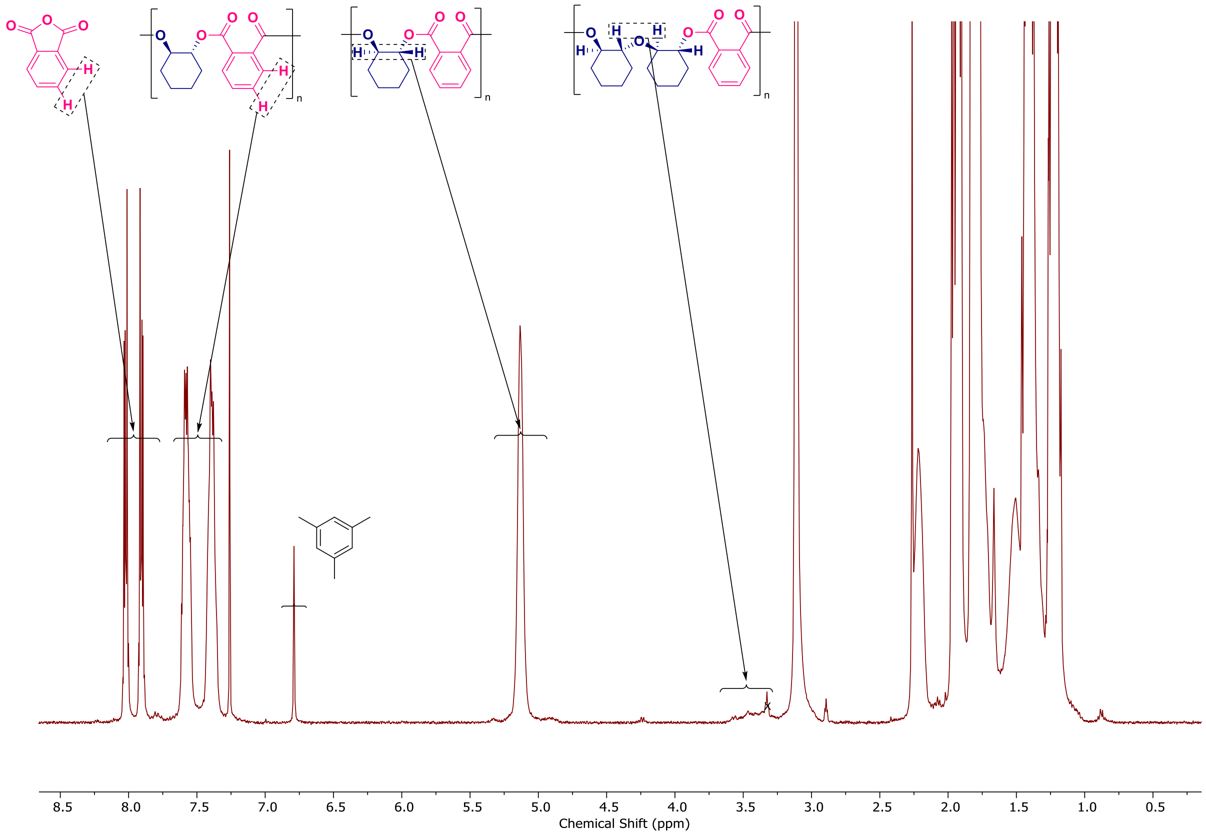

Figure S55. Excerpt of ^{1}H NMR spectrum (400 MHz, $CDCl_3$, 298 K) of reactant and products of CHO/PA copolymerization.

Table S3. Rates for CHO/PA ROCOP by complex **4**.

Entry	Conditions		Polymerization Results				
	[cat] : [PA] : [CHO]	t (h)	Conversion of PA (%)[b]	TOF for PA (h^{-1})[c]	Polyester Selectivity (%)[d]	$M_{n,GPC}$ (g mol^{-1}) [*Đ*][e]	$M_{n,Theory}$ (g mol^{-1})[f]
a	1:100:500	1.5	69	46	59	3,500 [1.57]	8,500
b	1:0:500	2.5	<1	0	-	-	-
c[a]	1:1000:5000	9.8	90	92	36	23,000 [1.66]	110,000

[a] Scale of reaction doubled, v(CHO) = 2 mL. [b] Determined by ^{1}H NMR (298 K, 400 MHz, $CDCl_3$) spectroscopy; comparison of PA monomer peaks at 8.06-8.00 and 7.94- 7.88 ppm to polymer peaks at 7.63-7.52 and 7.44-7.34 ppm. [c] Turnover frequency = TON/time(h). TON determined *via* Conversion*[monomer] (where [] is the equivalence). [d] Selectivity for polyester over polyether formation. Determined by ^{1}H NMR spectroscopy (298 K, 400 MHz, CDCl3); polyester 5.22-5.04 ppm vs. ether linkages 3.8-3.2 ppm. [e] Determined by GPC in THF, 30 °C, calibrated using PS standards. [f] Determined using (TON*M_n(repeatunit))/[Catalyst].

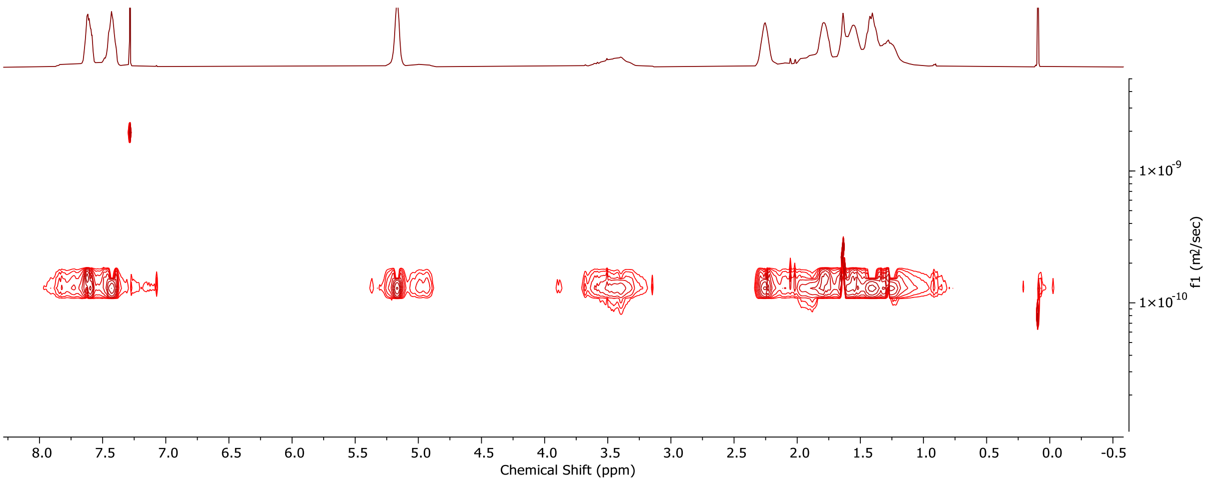

Figure S56. DOSY ^{1}H NMR spectrum ($CDCl_3$) of polymer formed by PA/CHO ROCOP using catalyst **4**. A single diffusion coefficient is observed for the polymer indicating that the ether and ester linkages are both within the same polymer.

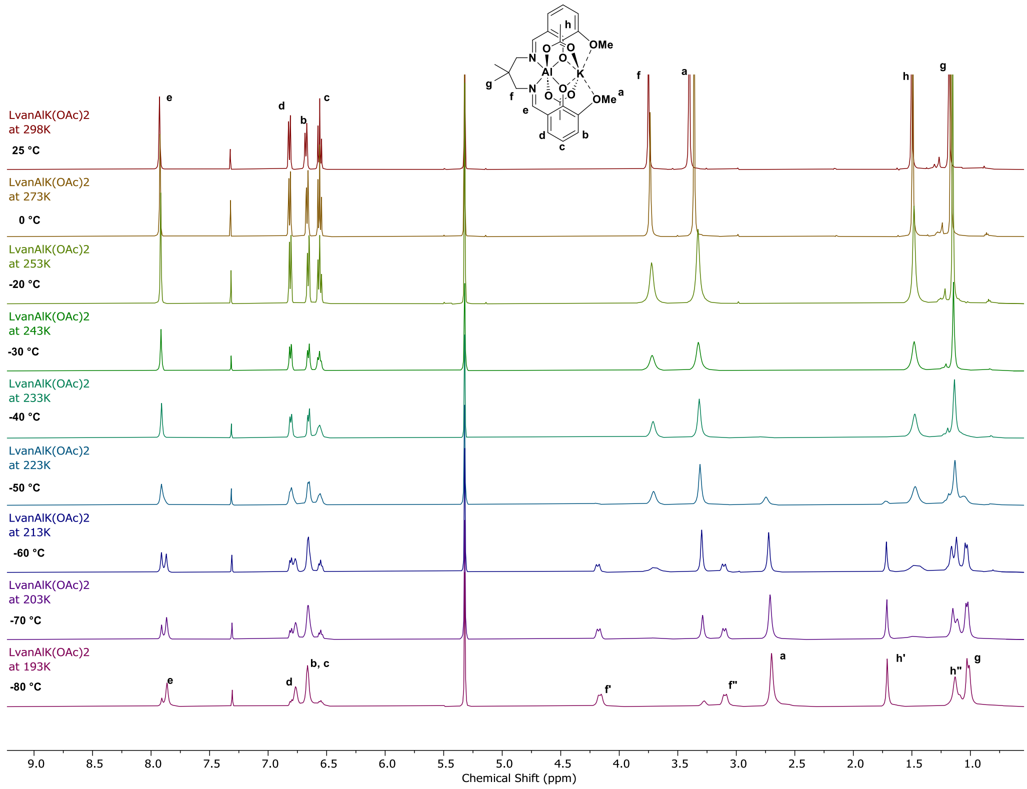

Figure S57. VT ^{1}H NMR spectra (400 MHz, CD_2Cl_2) of **1.1**. This shows a possible phase transition beginning at -40 °C but no indication of freezing out of fluxionality of the 6m metallocycle even at -80 °C.

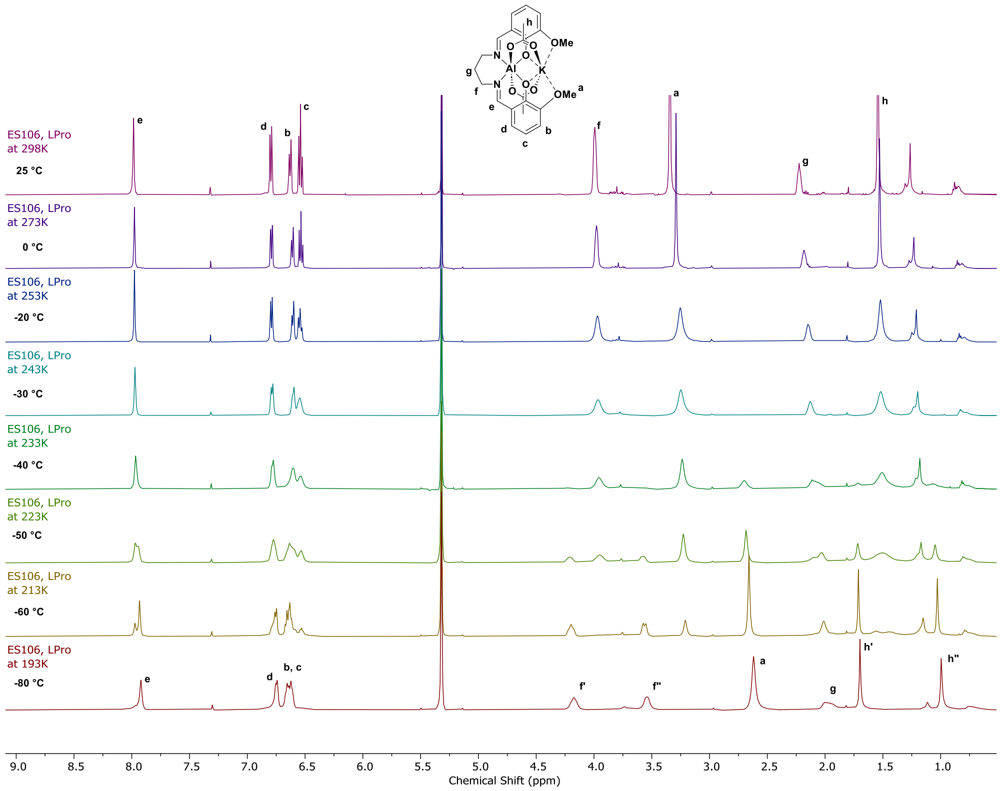

Figure S58. VT ^{1}H NMR spectra (400 MHz, CD_2Cl_2) of **5**. This shows a possible phase transition beginning at -40 °C but no indication of freezing out of fluxionality of the 6m metallocycle even at -80 °C.

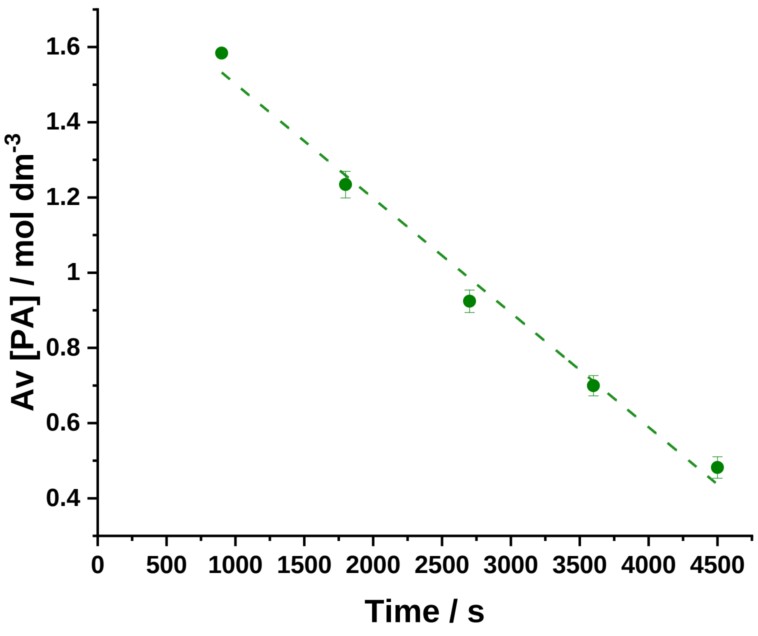

Figure S59. Graph of [PA] against time for CHO/PA ROCOP catalysed by complex **2**.

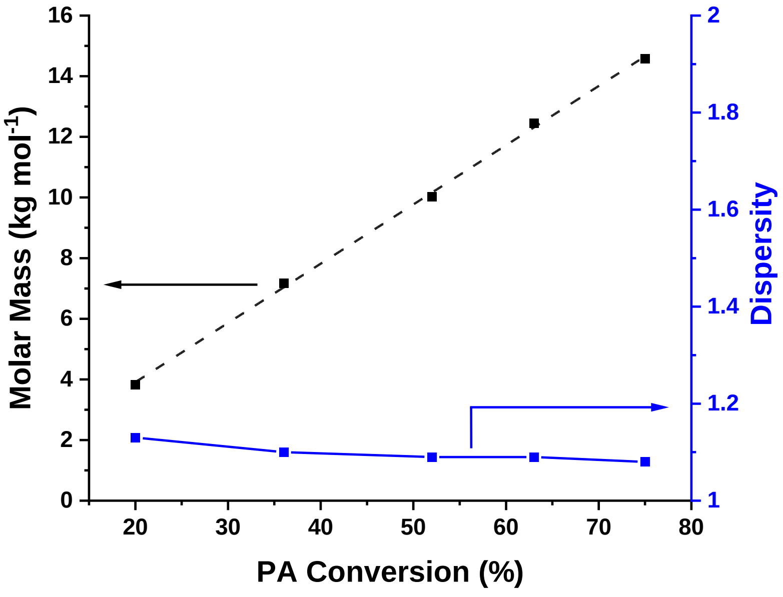

Figure S60. Plot of change in polymer molar mass and dispersity with increasing PA conversion for complex **2**.

Table S4. Rates for the ROCOP of CHO/PA using complex **1** with different CHD loadings.

Entry	Conditions		Polymerisation Data			
	CHD equiv. (#)	[CHD] / mol dm^{-3}	TOF $(h^{-1})^a$	k_{obs} (mol dm^{-3} s^{-1} x 10^{-3})[b]	Relative Rate	PE select. (%)[c]
a	0	0	309	0.425 (± 0.009)	1	> 99
b	10	0.0494	510	0.70 (± 0.07)	1.7	> 99
c	20	0.0988	714	0.98 (± 0.07)	2.3	> 99
d	50	0.247	814	1.12 (± 0.04)	2.6	> 99
e	100	0.494	1020	1.41 (± 0.08)	3.3	> 99
f	200	0.988	1330	1.83 (± 0.07)	4.3	> 99
g	400	1.98	1330	1.83 (± 0.07)	4.3	> 99

[**1**]: [CHD] : [anhydride]: = 1:#:400, 1 mL CHO such that [**1**] = 4.9 mmol, 100 °C [a] Turnover frequency from the gradient of TON vs time (h). TON = (conversion (%) / 100 (%)) * ([anhydride] / [**1**]). Conversion determined by ^{1}H NMR spectroscopy (298 K, 400 MHz, $CDCl_3$) by comparison of the normalised integrals of PA peaks, at 8.06-8.00 and 7.94- 7.88 ppm, vs. equivalent polymer peaks, 7.63-7.52 and 7.44-7.34 ppm. [b] Determined as the gradient of fits to plots of [PA] (mol dm^{-3}) vs time (s). Errors from the error in fit of the linear gradient of [PA] (mol dm^{-3}) vs t (s). [c] Selectivity for polyester over ether linkage formation. Determined by ^{1}H NMR spectroscopy (298 K, 400 MHz, $CDCl_3$) by comparison of polyester peaks (5.22-5.04 ppm) vs. any ether linkages (3.8-3.2 ppm).

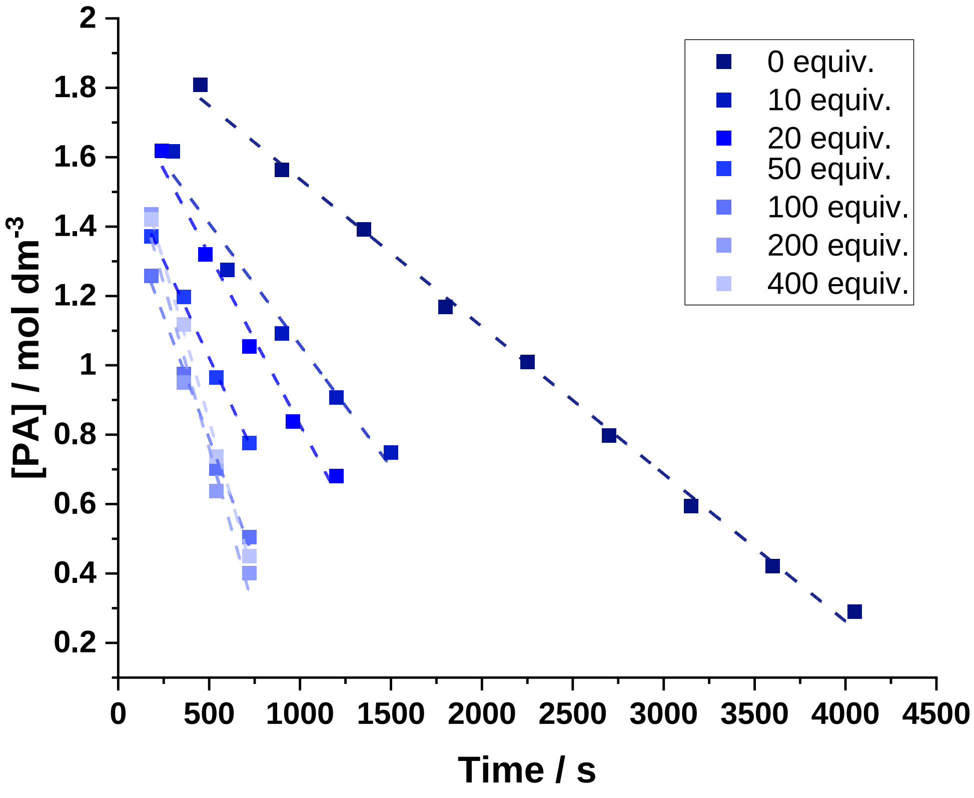

Figure S61. Graph showing the linear correlation between [PA] (mol dm^{-3}) and time (s) for all different [CTA]. [**1**]: [CHD] : [anhydride]: = 1:#:400, 1 mL CHO such that [**1**] = 4.9 mmol, 100 °C.

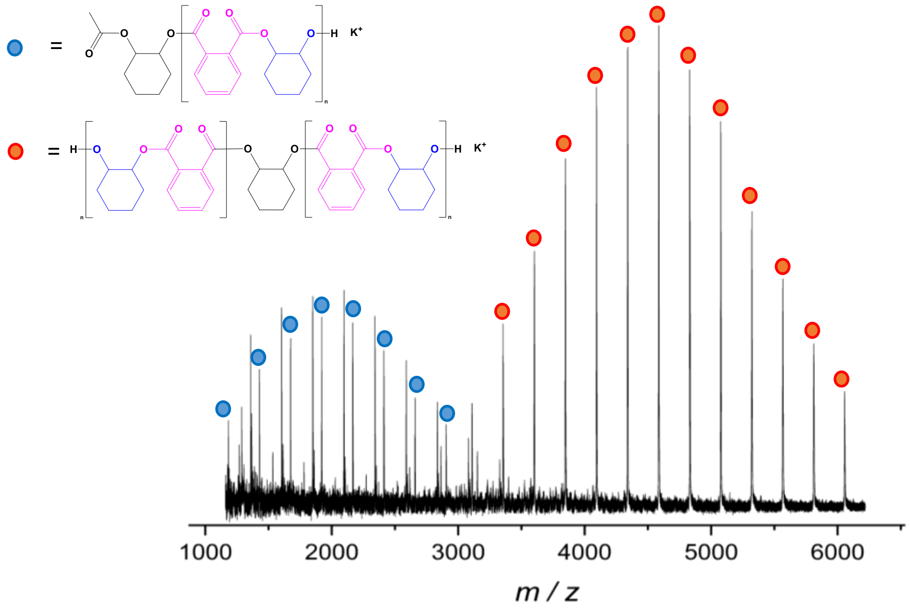

Figure S62. MALDI-ToF of PCHPE obtained with complex **1**. Conditions: [1] : [CHD] : [PA] : [CHO] = 1 : 20 : 400 : 2000, 45 min, T = 100 °C. $M_{n,calc}$(repeat unit) = 245.98 g mol^{-1}, $M_{n,theoretical}$(repeat unit) = 246.26 g mol^{-1}. $M_{n,calc}$(end group = blue) = 197.71 g mol^{-1}, $M_{n,theoretical}$(end group = AcOH + K^+) = 197.06 g mol^{-1}. Red distribution attributed to CHD initiating species.

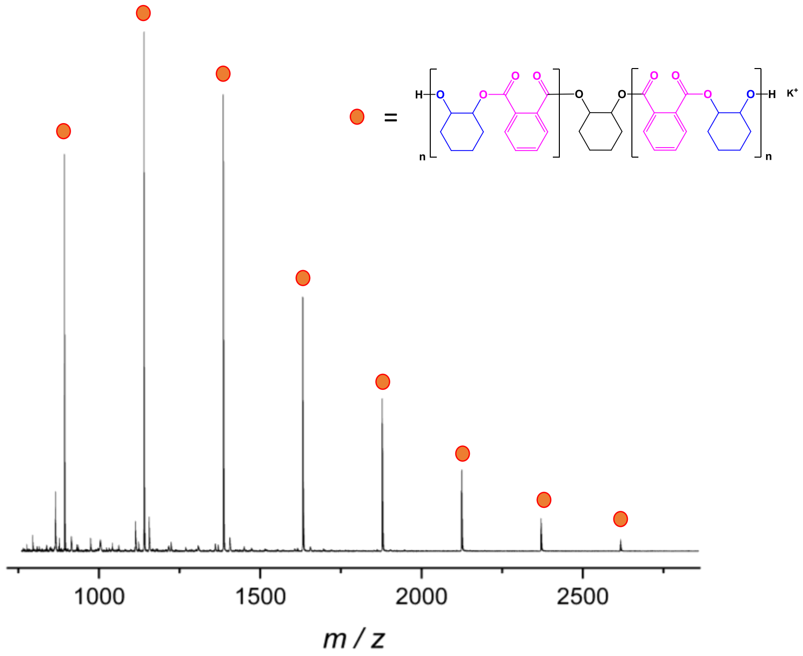

Figure S63. MALDI-ToF of PCHPE obtained with complex **1**. Conditions: [1] : [CHD] : [PA] : [CHO] = 1 : 100 : 400 : 2000, 26 min, T = 100 °C. $M_{n,calc}$(repeat unit) = 246.07 g mol^{-1}, $M_{n,theoretical}$(repeat unit) = 246.26 g mol^{-1}. $M_{n,calc}$(end group = red) = 155.08 g mol^{-1}, $M_{n,theoretical}$(end group = CHD + K^+) = 155.05 g mol^{-1}.

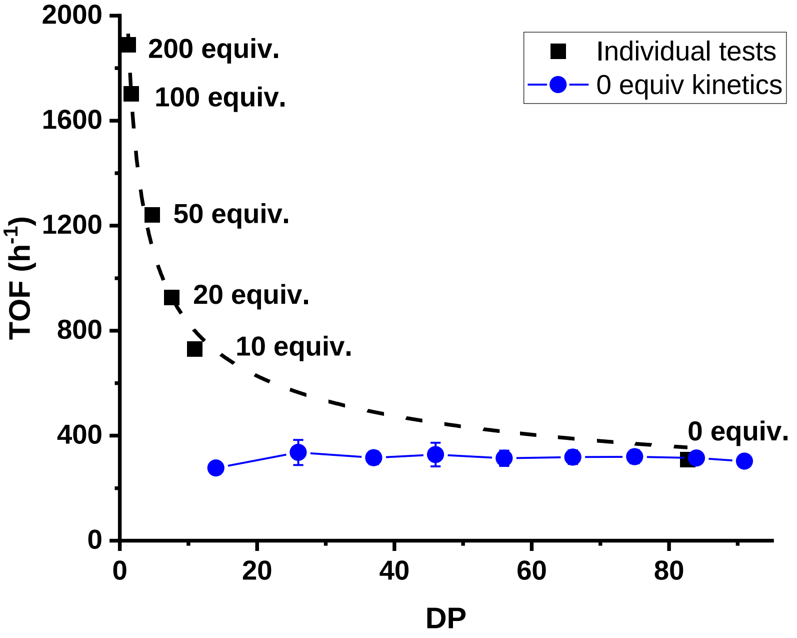

Figure S64. Graph of TOF against Degree of Polymerisation (DP). The points represent individual point TOFs and the corresponding DP for that aliquot. The black squares are individual tests at different CHD loadings. The blue circles come from aliquots from the kinetic study of 0 equiv CHD loading. This shows that TOF remains constant across a range of DPs with a given CHD loading (blue), contrary to the trend observed with different CHD loadings (black).

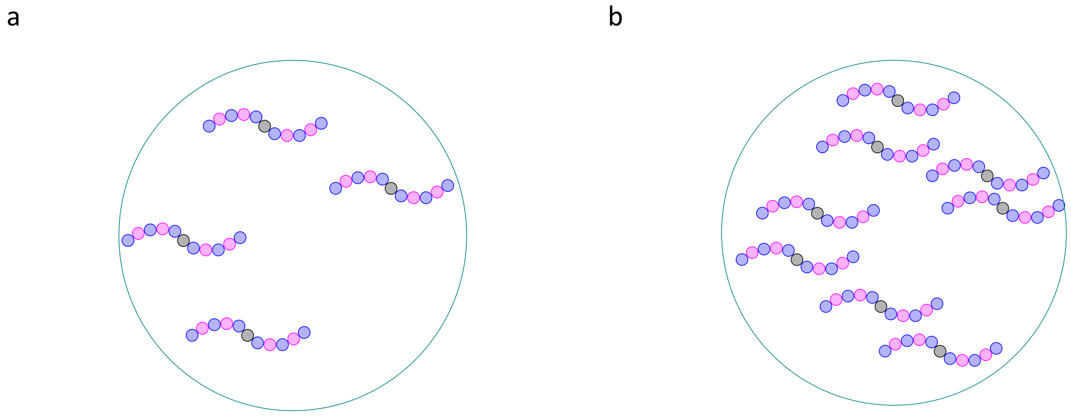

Figure S65. A pictorial representation of DP = 10 at two different CTA concentrations, showing how, at the same DP, the higher concentration of CTA very logically leads to a higher concentration of polymer chains and thus presumably a higher viscosity.

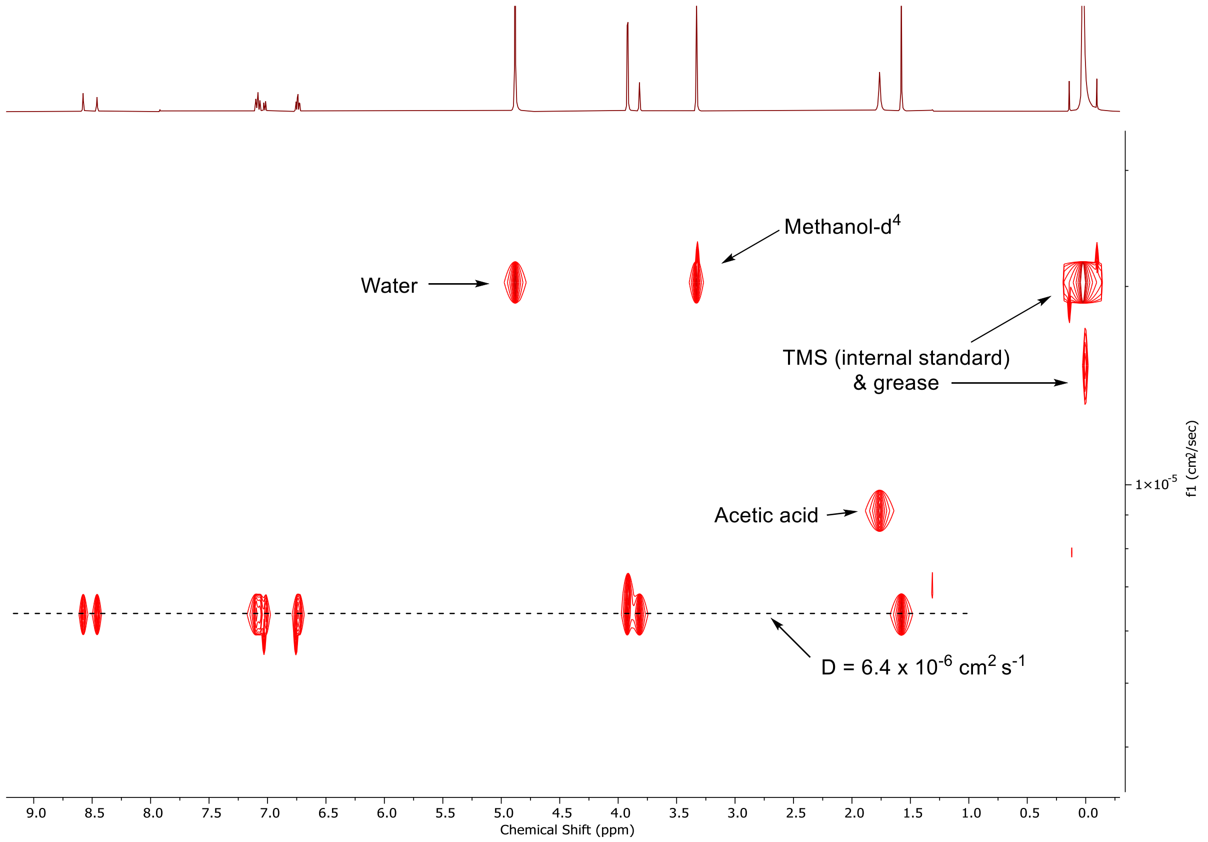

Figure S66. DOSY NMR spectrum (500 MHz, MeOD-d^4, 298 K) of complex **1**. The D value obtained from this experiment and the Stokes-Einstein equation yields a solvation radius of 6.2 Å. This is close to the radius of the dimeric unit cell 6.7 Å.

Table S5. Rates for the ROCOP of CHO/PA using complex **1.1** with different CHD loadings.

Entry	CHD equiv. (#)	Time (min)	TON[a]	TOF (h^{-1})[b]	Relative Rate	PE Select. (%)[c]	$M_{n, GPC}$ (g mol^{-1}) [$Đ_M$] at full conversion[d]	$M_{n, Theory}$ (g mol^{-1})[e]
a[11]**	0	15	268	1072	1	>99	-	49,000
b	20	5	111	1336	1.25	>99	3,500 [1.14]	4,600
c	100	5	145	1744	1.63	>99	900 [1.14]	1,100
d	200	5	155	1860	1.74	>99	540 [1.00]	600
e	400	5	103	1239	1.16	>99	-	360

[**1.1**]: [CHD] : [anhydride]: = 1:#:400, 1 mL CHO such that [**1.1**] = 4.9 mmol, 100 °C [a] TON = (conversion (%) / 100 (%)) * ([anhydride] / [**1**]). Conversion (%) determined by ^{1}H NMR (298 K, 400 MHz, $CDCl_3$) spectroscopy; comparison of PA monomer peaks at 8.06-8.00 and 7.94- 7.88 ppm to polymer peaks at 7.63-7.52 and 7.44-7.34 ppm. [b] Turnover frequency = TON/time(h). TON determined *via* Conversion*[Anhydride]. [c] Selectivity for polyester linkages by analysis of the ^{1}H NMR spectrum (298 K, 400 MHz, CDCl3); polyester 5.22-5.04 ppm vs. ether linkages 3.8-3.2 ppm. [d] Determined by GPC in THF, 30 °C, calibrated using PS standards. [e] Determined using (TON*$M_{n,repeatunit}$)/([Catalyst] + [CHD]) + $M_{n, CHD}$.

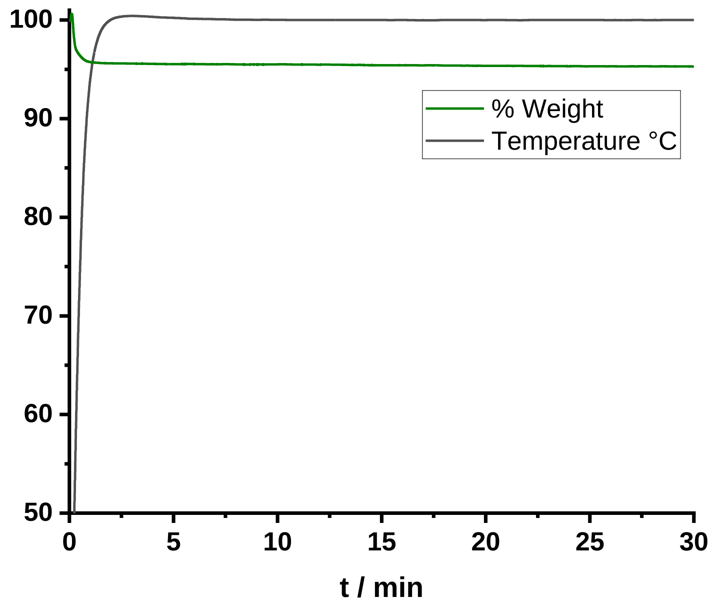

Figure S67. TGA of 100 °C isotherm of **1**. Initial mass drop upon heating attributed to solvent.

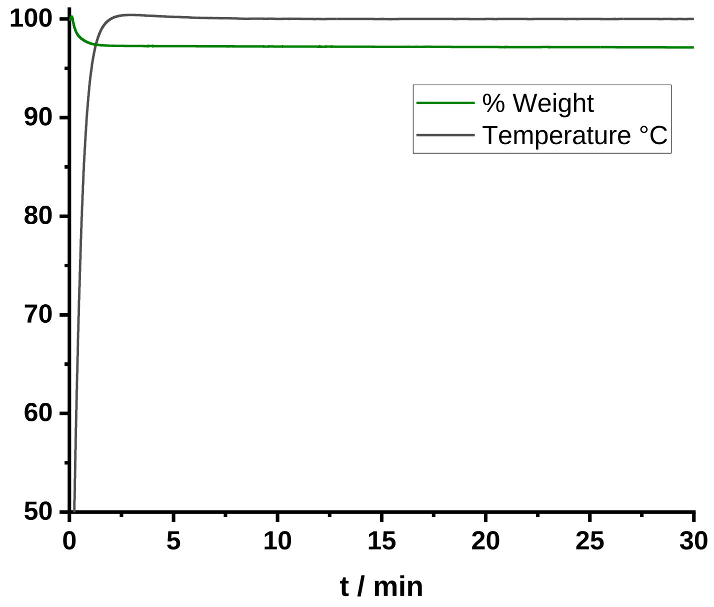

Figure S68. TGA of 100 °C isotherm of **1** + glycerol ethoxylate (GEO).

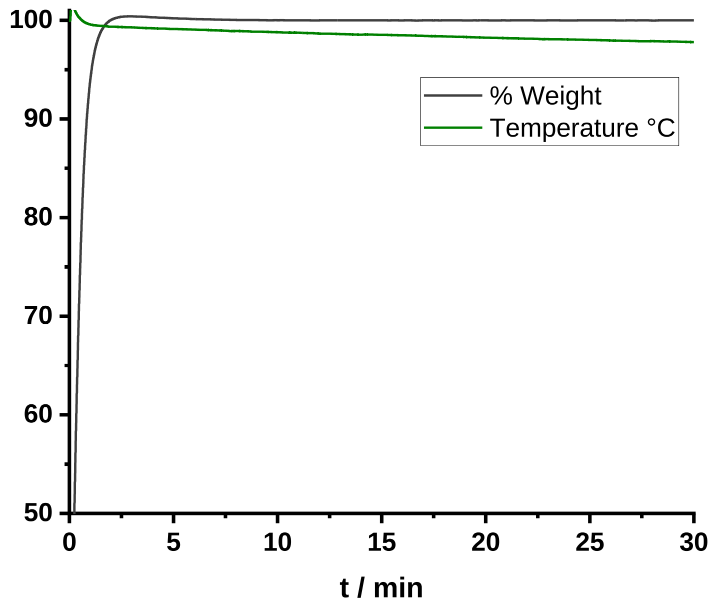

Figure S69. TGA of 100 °C isotherm of **1.1**.

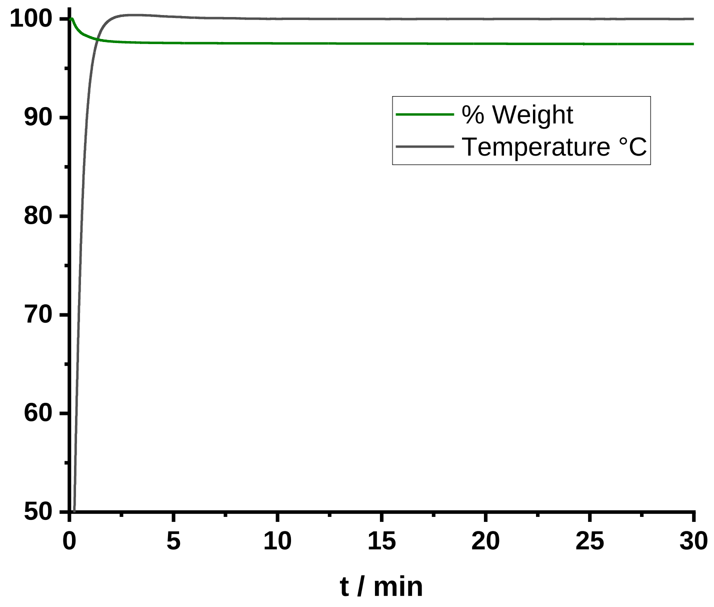

Figure S70. TGA of 100 °C isotherm of **1.1** + glycerol ethoxylate (GEO).

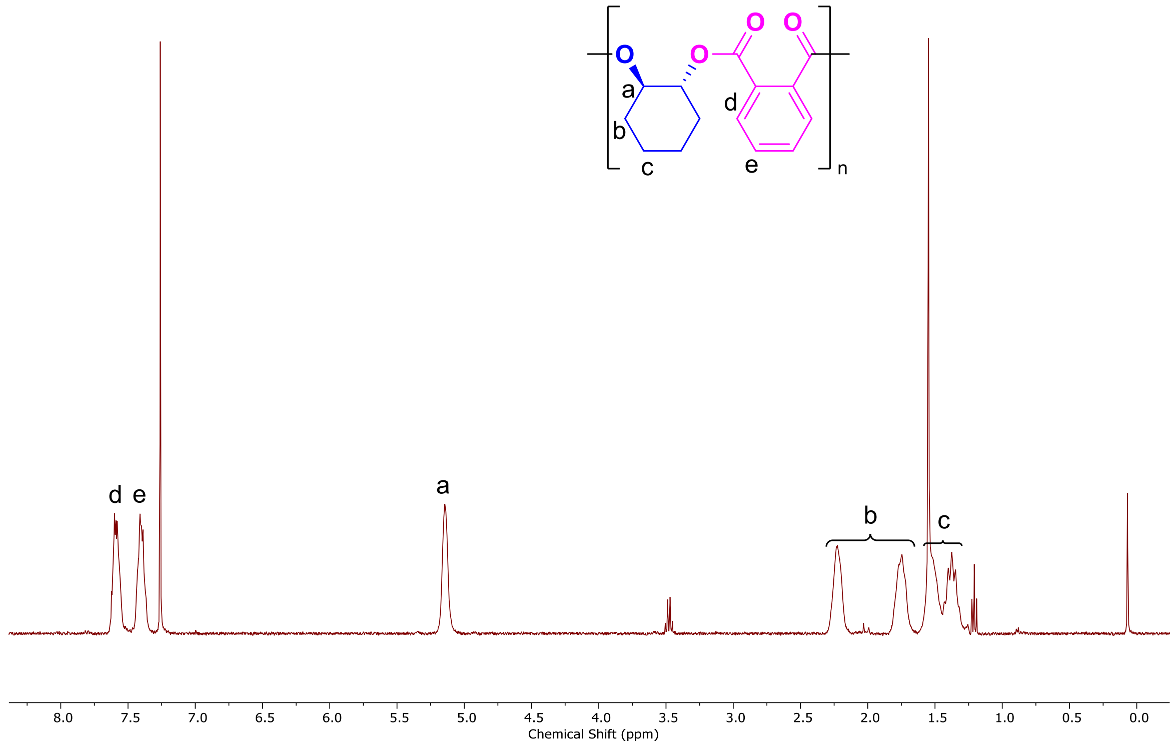

Figure S71. ^{1}H NMR spectrum (400 MHz, $CDCl_3$, 298 K) of isolated polymer from the polymerisation of CHO/PA.

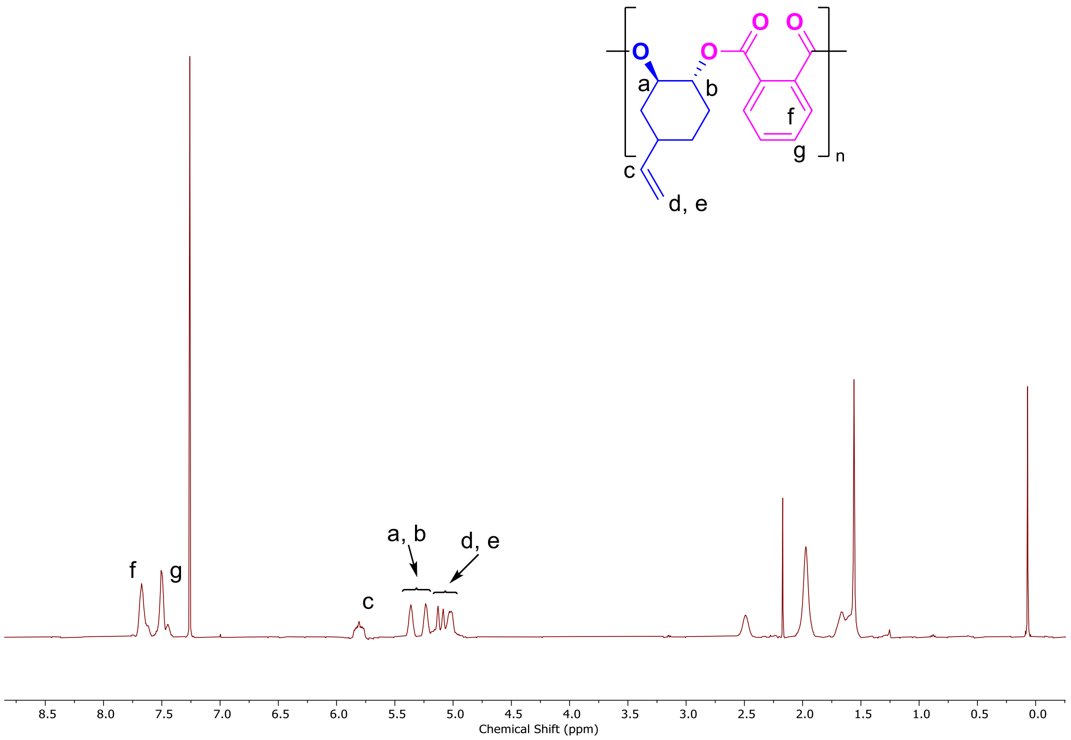

Figure S72. ^{1}H NMR spectrum (400 MHz, $CDCl_3$, 298 K) of isolated polymer from the polymerisation of vCHO/PA.

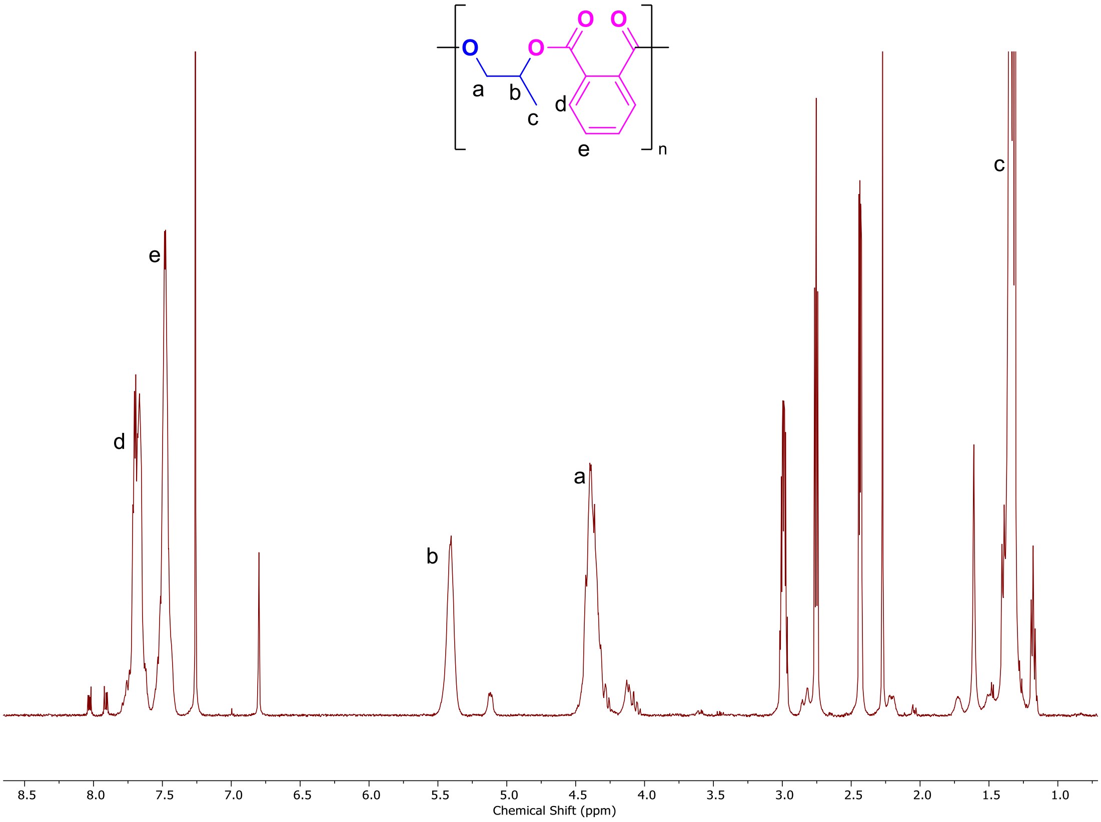

Figure S73. ^{1}H NMR spectrum (400 MHz, $CDCl_3$, 298 K) of PO/PA polymerisation reaction mixture (at 99 % conversion).

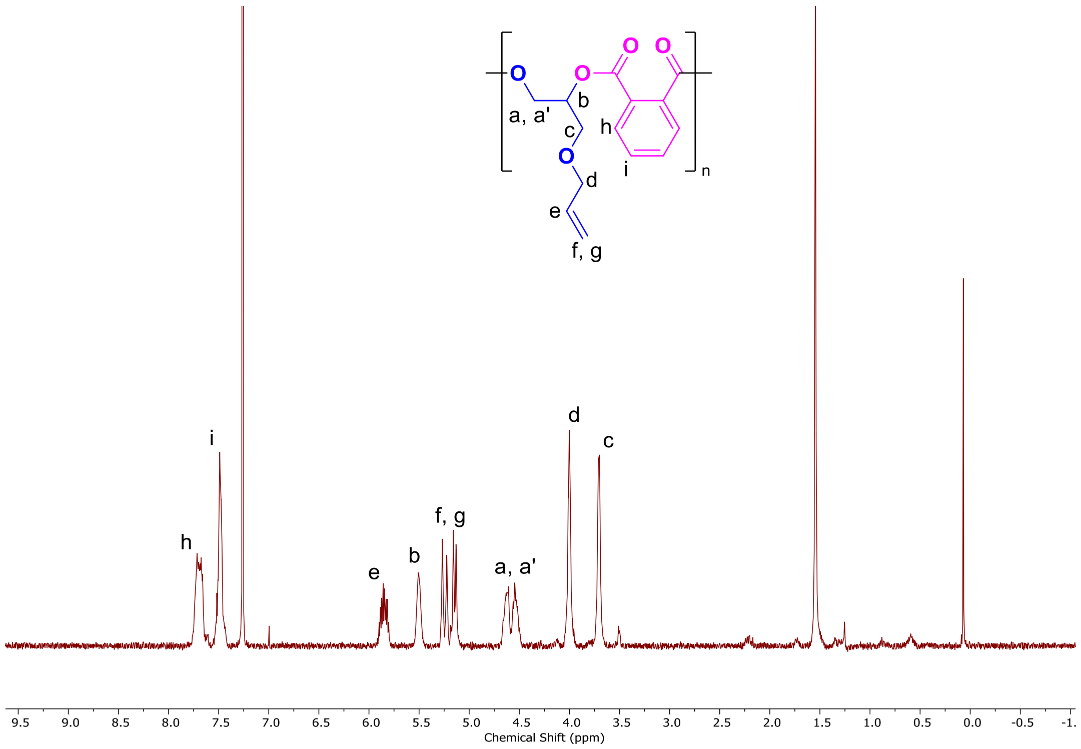

Figure S74. ^{1}H NMR spectrum (400 MHz, $CDCl_3$, 298 K) of isolated polymer from the polymerisation of AGE/PA.

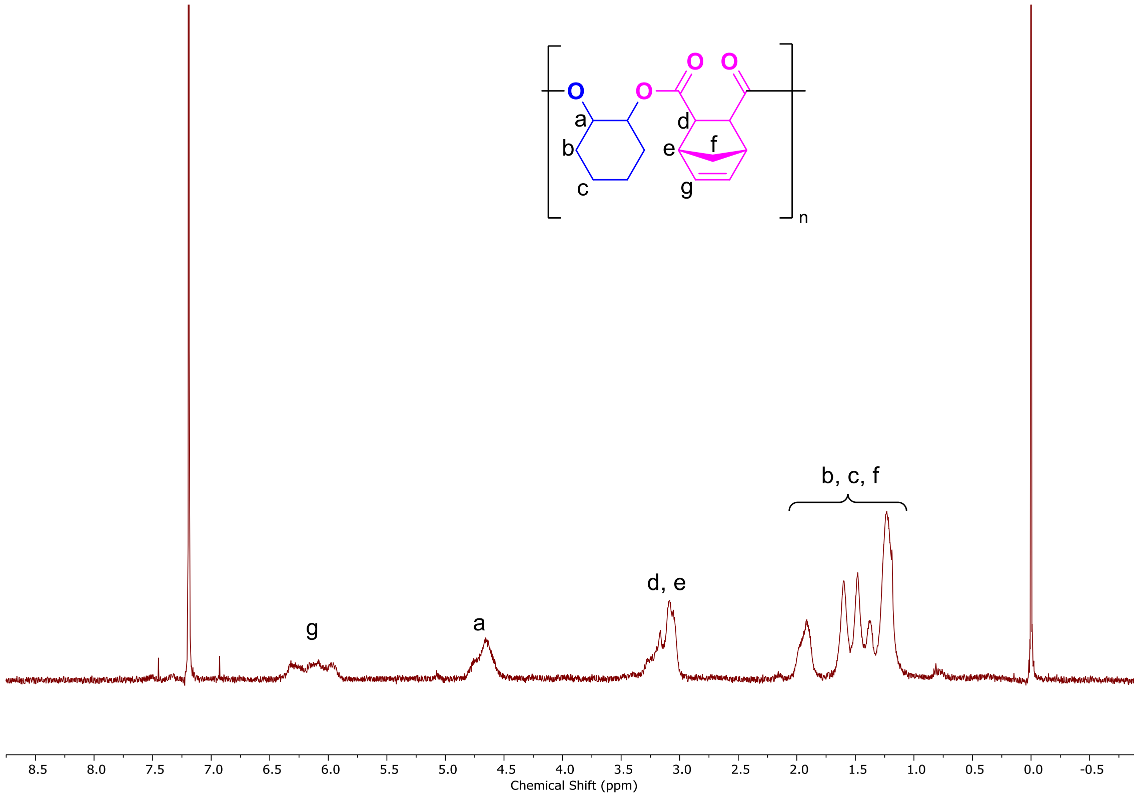

Figure S75. ^{1}H NMR spectrum (400 MHz, $CDCl_3$, 298 K) of isolated polymer from the polymerisation of CHO/NBA.

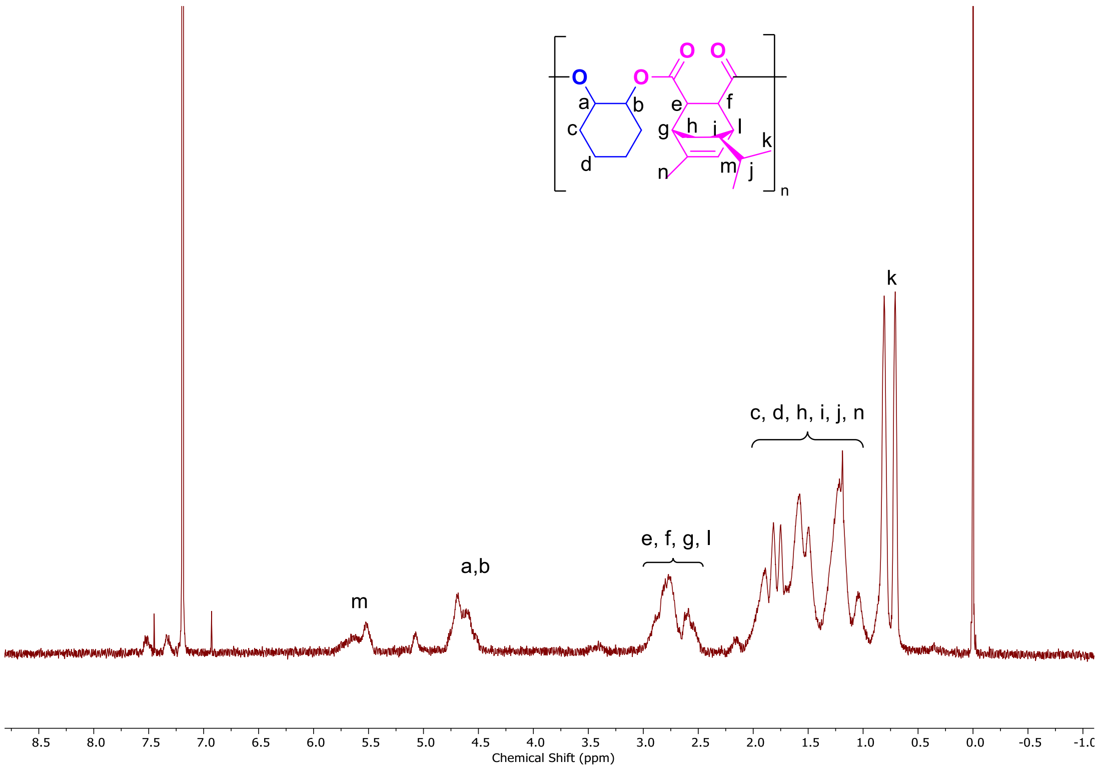

Figure S76. ^{1}H NMR spectrum (400 MHz, $CDCl_3$, 298 K) of isolated polymer from the polymerisation of CHO/TGA.

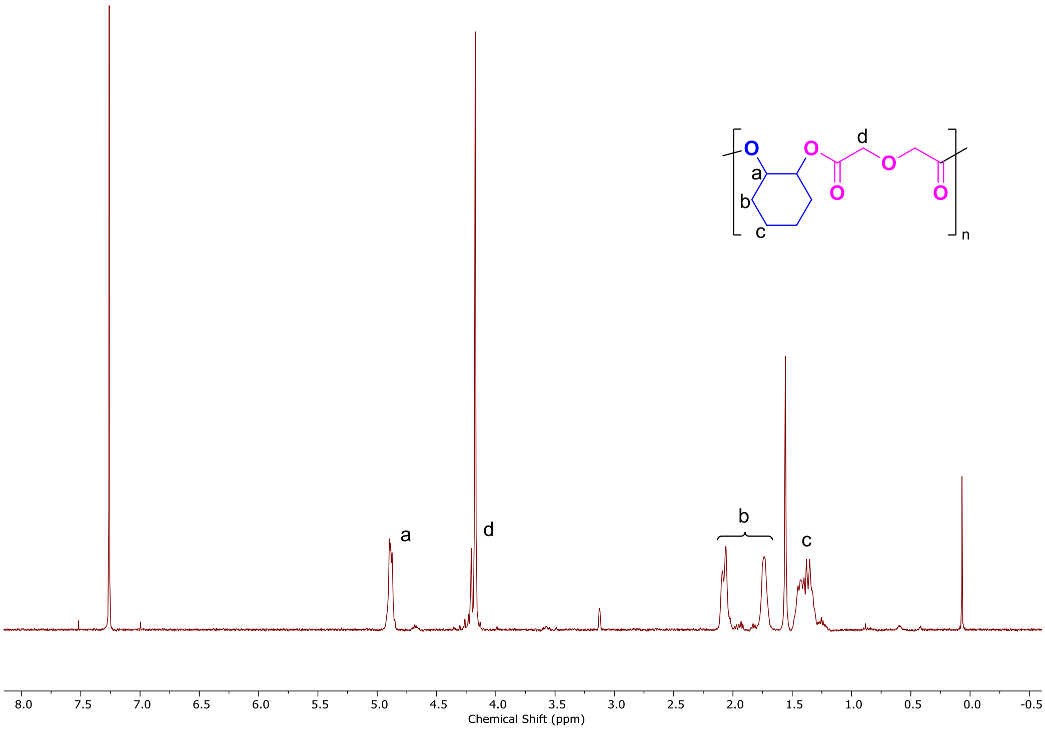

Figure S77. ^{1}H NMR spectrum (400 MHz, $CDCl_3$, 298 K) of isolated polymer from the polymerisation of CHO/DGA.

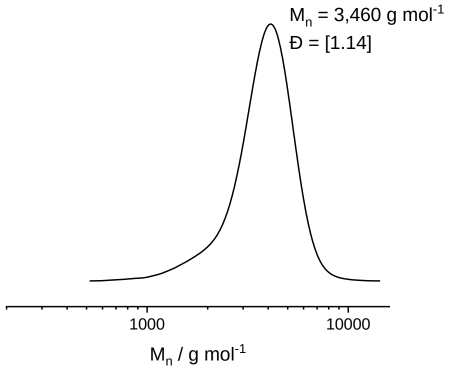

Figure S78. GPC plot of CHO/PA polymerisation at full conversion.

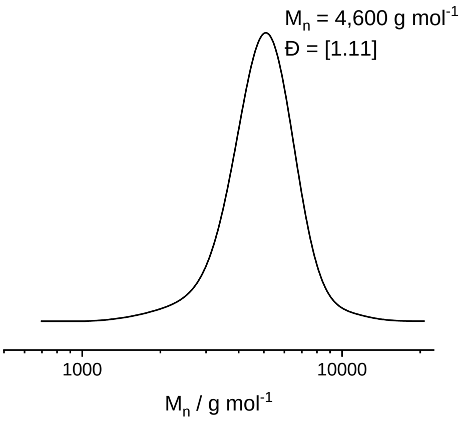

Figure S79. GPC plot of vCHO/PA polymerisation at full conversion.

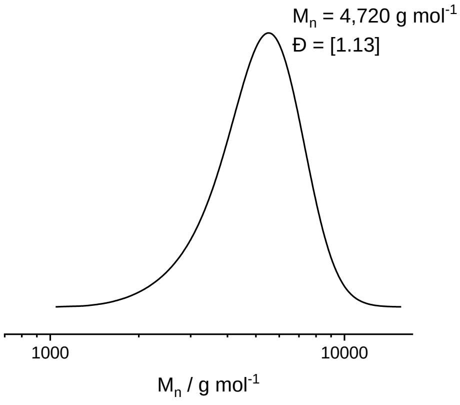

Figure S80. GPC plot of PO/PA polymerisation at full conversion.

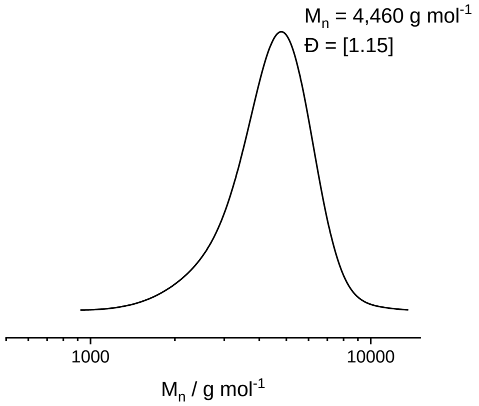

Figure S81. GPC plot of AGE/PA polymerisation at full conversion.

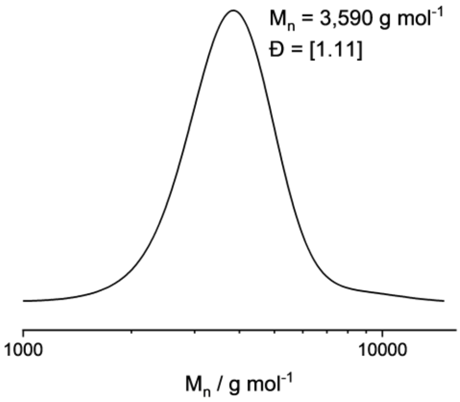

Figure S82. GPC plot of CHO/NBA polymerisation at full conversion.

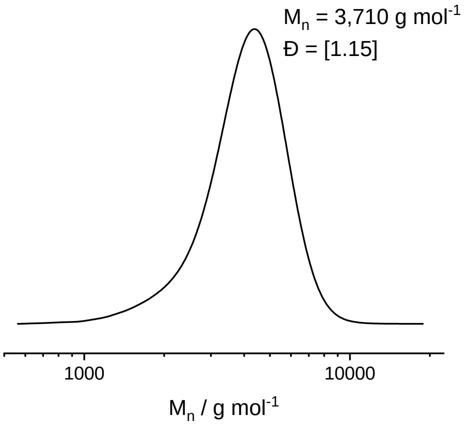

Figure S83. GPC plot of CHO/TCA polymerisation at full conversion.

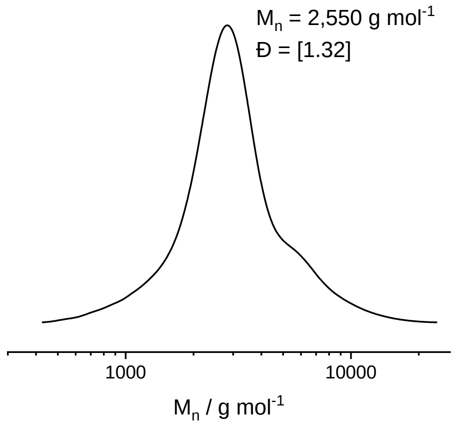

Figure S84. GPC plot of CHO/DGA polymerisation at full conversion.

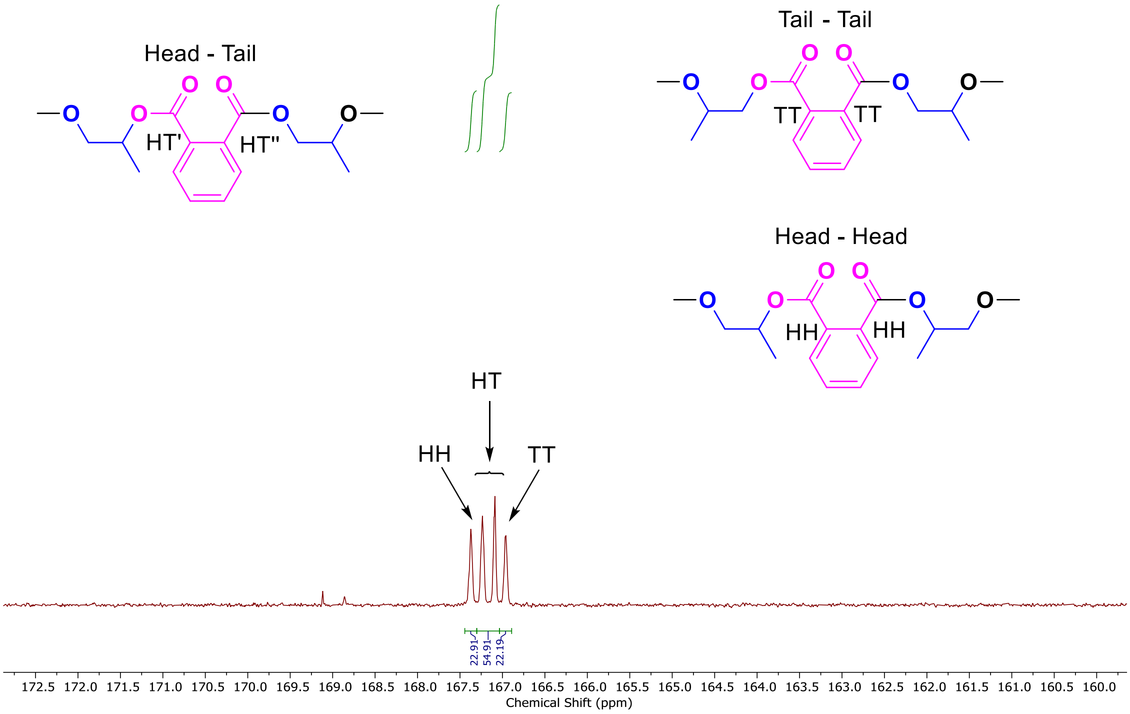

Figure S85. Quantitative $^{13}C\{^1H\}$ NMR spectrum (100 MHz, $CDCl_3$, 298 K) of isolated PO/PA polymer to find head-tail regioselectivity.

Table S6. Rates for the ROCOP of epoxide/PA using complex **1** with different CHD loadings.

Entry	Conditions		Polymerisation Data			
	Monomers	CHD equiv.	Time (min)	TON[b]	TOF (h^{-1})[c]	PE Select. (%)[d]
a	vCHO/PA	0	100	296	178	>99
b	vCHO/PA	20	15	225	901	>99
c[a]	PO/PA	0	1,020	336	20	>99
d[a]	PO/PA	20	420	198	28	>99
e[a]	PO/PA	100	300	200	40	>99

[a] Reaction run at 60 °C. [b] Calculated using Conversion*[monomer] (where [] is the equivalence). Conversion determined by ^{1}H NMR (298 K, 400 MHz, CDCl3) spectroscopy; comparison of PA monomer peaks at 8.06-8.00 and 7.94- 7.88 ppm to polymer peaks at 7.63-7.52 and 7.44-7.34 ppm. [c] Turnover frequency = TON/time(h). [d] Selectivity for polyester over polyether formation. Determined by ^{1}H NMR spectroscopy (298 K, 400 MHz, CDCl3); polyester 5.22-5.04 ppm vs. ether linkages 3.8-3.2 ppm.

6. References

(1) Dakshinamoorthy, D.; Weinstock, A. K.; Damodaran, K.; Iwig, D. F.; Mathers, R. T. Diglycerol-Based Polyesters: Melt Polymerization with Hydrophobic Anhydrides. *ChemSusChem* **2014**, *7* (10), 2923-2929. DOI: 10.1002/cssc.201402249.

(2) Cosier, J.; Glazer, A. M. A nitrogen-gas-stream cryostat for general X-ray diffraction studies. *J. Appl. Crystallogr.* **1986**, *19* (2), 105-107. DOI: 10.1107/s0021889886089835.

(3) *CrysAlisPRO*; Oxford Diffraction / Agilent Technologies UK Ltd, Yarnton, England.

(4) a) Sheldrick, G. M. Crystal structure refinement withSHELXL. *Acta. Cryst.* **2015**, *C71* (1), 3-8. DOI: 10.1107/s2053229614024218. b) SHELXTL v5.1, Bruker AXS, Madison, WI, 1998. c) Sheldrick, G. M. SHELXT– Integrated space-group and crystal-structure determination. *Acta. Cryst.* **2015**, *A71* (1), 3-8. DOI: 10.1107/s2053273314026370. d) Dolomanov, O. V.; Bourhis, L. J.; Gildea, R. J.; Howard, J. A. K.; Puschmann, H. OLEX2: a complete structure solution, refinement and analysis program. *J. Appl. Crystallogr.* **2009**, *42* (2), 339-341. DOI: 10.1107/s0021889808042726.

(5) Aguiari, A.; Bullita, E.; Casellato, U.; Guerriero, P.; Tamburini, S.; Vigato, P. A. Macrocyclic and macroacyclic compartmental Schiff bases: synthesis, characterization, X-ray structure and interaction with metal ions. *Inorg. Chim. Acta* **1992**, *202* (2), 157-171. DOI: 10.1016/s0020-1693(00)86831-0.

(6) Thevenon, A.; Garden, J. A.; White, A. J.; Williams, C. K. Dinuclear Zinc Salen Catalysts for the Ring Opening Copolymerization of Epoxides and Carbon Dioxide or Anhydrides. *Inorg. Chem.* **2015**, *54* (24), 11906-11915. DOI: 10.1021/acs.inorgchem.5b02233.

(7) Bermejo, M. R.; Fernández, M. I.; Gómez-Fórneas, E.; González-Noya, A.; Maneiro, M.; Pedrido, R.; Rodríguez, M. J. Self-Assembly of Dimeric MnIII–Schiff-Base Complexes Tuned by Perchlorate Anions. *Eur. J. Inorg. Chem.* **2007**, *2007* (24), 3789-3797. DOI: 10.1002/ejic.200700199.

(8) Jiang, Y.; Gong, L.; Feng, X.; Hu, W.; Pan, W.; Li, Z.; Mi, A. Salen-Ti(OR)4 complex catalysed trimethylsilylcyanation of aldehydes. *Tetrahedron* **1997**, *53* (42), 14327-14338. DOI: 10.1016/s0040-4020(97)00984-8.

(9) Routaray, A.; Nath, N.; Mantri, S.; Maharana, T.; Sutar, A. K. Synthesis and structural studies of copper(II) complex supported by –ONNO– tetradentate ligand: Efficient catalyst for the ring-opening polymerization of lactide. *Chin. J. Catal.* **2015**, *36* (5), 764-770. DOI: 10.1016/s1872-2067(14)60293-x.

(10) Diment, W. T.; Gregory, G. L.; Kerr, R. W. F.; Phanopoulos, A.; Buchard, A.; Williams, C. K. Catalytic Synergy Using Al(III) and Group 1 Metals to Accelerate Epoxide and Anhydride Ring-Opening Copolymerizations. *ACS Catal.* **2021**, *11* (20), 12532-12542. DOI: 10.1021/acscatal.1c04020.
